# Domino reactions of 2*H*-azirines with acylketenes from furan-2,3-diones: Competition between the formation of *ortho*-fused and bridged heterocyclic systems

**DOI:** 10.3762/bjoc.10.74

**Published:** 2014-04-04

**Authors:** Alexander F Khlebnikov, Mikhail S Novikov, Viktoriia V Pakalnis, Roman O Iakovenko, Dmitry S Yufit

**Affiliations:** 1 Department of Chemistry, Saint-Petersburg State University, Universitetskii pr. 26, 198504 St. Petersburg, Russia; 2 Department of Chemistry, University of Durham, Durham, South Rd., DH1 3LE, UK

**Keywords:** acylketene, azirine, domino reaction, furandione

## Abstract

3-Aryl-2*H*-azirines react with acylketenes, generated by thermolysis of 5-arylfuran-2,3-diones, to give bridged 5,7-dioxa-1-azabicyclo[4.4.1]undeca-3,8-diene-2,10-diones and/or *ortho*-fused 6,6a,12,12a-tetrahydrobis[1,3]oxazino[3,2-*a*:3′,2′-*d*]pyrazine-4,10-diones. The latter compounds, with a new heterocyclic skeleton, are the result of the coupling of two molecules of azirine and two molecules of acylketene and can be prepared only from 3-aryl-2*H*-azirines having no electron-withdrawing groups in the aryl substituent. Calculations at the DFT B3LYP/6-31G(d) level for the various routes of bis[1,3]oxazino[3,2-*a*:3′,2′-*d*]pyrazine skeleton formation revealed a new domino reaction of 3-aryl-2*H*-azirines occurring in the presence of furandiones: acid-catalyzed dimerization to dihydropyrazine followed by consecutive cycloaddition of the latter to two molecules of acylketenes.

## Introduction

2*H*-Azirines, the most strained nitrogen unsaturated heterocyclic systems, are versatile building blocks for the construction of various heterocyclic nitrogen-containing compounds. Because 2*H*-azirines contain an activated C=N double bond and a lone pair of electrons on the nitrogen atom they are extremely reactive towards both electrophiles and nucleophiles. Though the three-membered ring can be preserved in some reactions, 2*H*-azirines mostly undergo ring cleavage to relieve the strain [1–21].

2*H*-Azirines can react with ketenes both with cleavage and preservation of the three-membered ring [22–26]. It was found that acylketenes, which are generated in situ from diazo ketones, undergo cycloaddition with 3-mono- and 2,3-disubstituted-2*H*-azirines to afford 2:1 or 1:1 adducts: 5,7-dioxa-1-azabicyclo[4.4.1]undeca-3,8-diene or 5-oxa-1-azabicyclo[4.1.0]hept-3-ene derivatives. From the results of DFT B3LYP/6-31G(d) computations a step-wise mechanism appears likely for the formation of [4 + 2]-monoadducts [22]. The main limitation for the synthetic application of the reaction is the nonselective mode of the Wolff rearrangement of the unsymmetrical diazo compounds. This generates a mixture of isomeric oxoketenes [27–29] and, as a result, a complex mixture of products is formed [22]. Moreover not all diazo compounds give oxoketenes easily [27–29]. In particular, unsubstituted acylketenes, the reactivity of which towards azirines is until now unknown, cannot be generated from diazo compounds. An alternative source of acylketenes can be furan-2,3-diones, which have been used in reactions with nucleophiles and various cycloadditions [30–32]. Aiming to broaden the scope of the reaction of acylketenes with 2*H*-azirines we tried to use furan-2,3-diones instead of diazo compounds as the source of ketenes.

## Results and Discussion

**Scheme 1:**
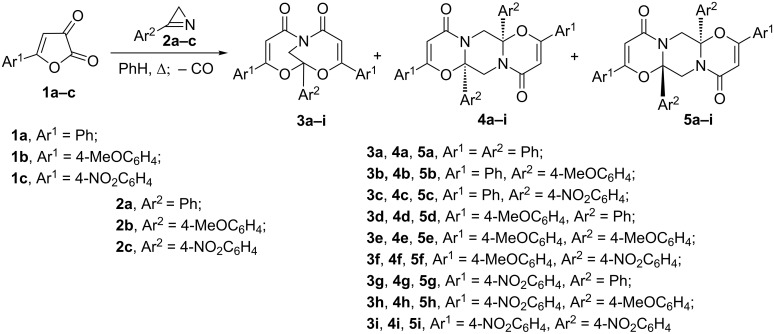
Reactions of furan-2,3-diones **1** and azirines **2**.

Unexpectedly, with a new source of acylketenes in addition to predictable products (derivatives of 5,7-dioxa-1-azabicyclo[4.4.1]undeca-3,8-diene) derivatives of 4,11-dioxa-1,8-diazatricyclo[8.4.0.0^3,8^]tetradeca-5,12-diene, a new heterocyclic system, were formed. Boiling a benzene solution of furan-2,3-dione **1a** and azirine **2a** (1:1) for 0.5 h gave a mixture of compounds **3a–5a**, which were isolated by column chromatography (Scheme 1).

To find the optimal reaction conditions a series of experiments was performed with furan-2,3-dione **1a** and azirine **2a** in different solvents (benzene, toluene, cyclohexane, THF, nitromethane) monitoring the reaction by ^1^H NMR using 1-methylnaphthalene as internal standard. ^1^H NMR spectra of the new compounds **4a** and **5a** have clearly distinguishable signals for the methylene protons. Thus, in *cis*-diastereomer **4а** the chemical shifts of the doublet signals for the protons of the CH_2_-groups differ by more than 2 ppm (3.26, 5.62 ppm), whereas in *trans*-diastereomer (**5а**) they represent an AB-system (4.56, 4.68 ppm). Attempts to initiate the reaction by UV-irradiation (at 20 or 50 °C) or catalysis by compounds of transition metals (Cu(acac)_2_, Fe(acac)_3_, Pd(bzac)_2_, Rh_2_(AcO)_4_, Cu(OTf)_2_, Pd/C) at 20 or 40 °C failed. Benzene was found to be a solvent of choice, and a 1:1 molar ratio of reagents results in the highest yields of the products (Table 1).

**Table 1:** Yields of products of the reaction of furan-2,3-dione **1a** and azirine **2a** in boiling benzene solution for 0.5 h according to ^1^H NMR.

Ratio **2a**:**1a**	Conversion of **2a** (%)	Yields^a^ of **3a, 4a, 5a**, %	Overall yields^a^ of **3a–5a**, %

1:2	100	2, 16, 9	27
1:1.5	89	5, 22, 17	44
1:1	42	19, 37, 15	71
1.5:1	57	2, 19, 11	32
2:1	43	4, 20, 13	37

^a^Yield based on consumed azirine **2a**.

**Figure 1:**
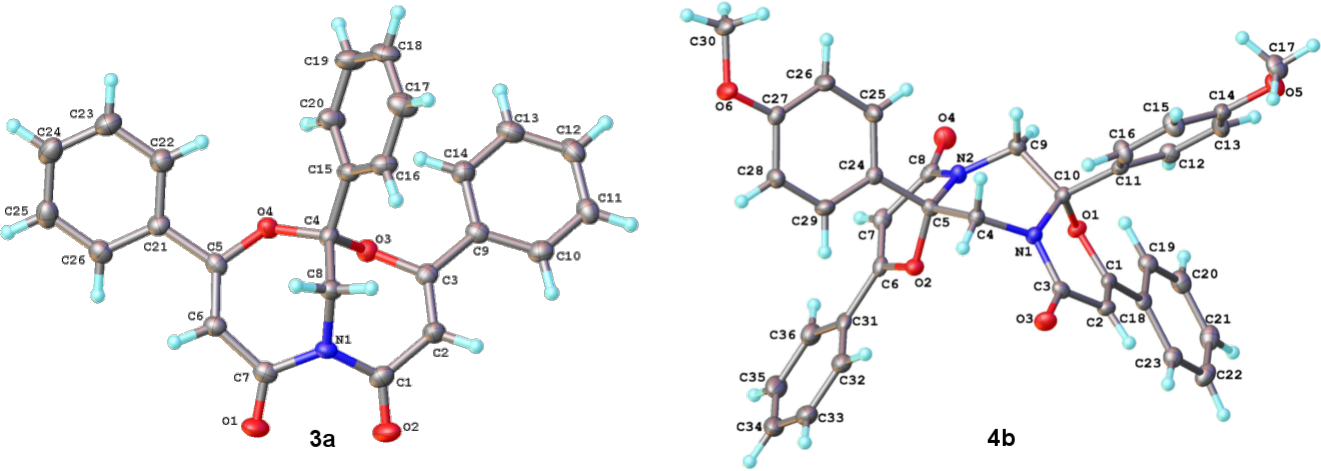
Molecular structures of compounds **3а, 4b**.

Reactions of azirines **2a–c** and furandiones **1a–c**, containing electron-donating and electron-withdrawing groups in the aryl rings, were studied to determine an influence of substituents with different electronic effects on the product distribution. The analytical and isolated yields of the reaction products are listed in Table 2. Compounds **3–5** were fully characterized using standard spectral methods. The structures of compounds **3а, 4b** were confirmed by X-ray analysis (Figure 1).

**Table 2:** Products of the reactions of azirines **2a–c** and furandiones **1a–c**.

Ar^1^	Ar^2^	Conversion of **2**, %	Analytical yields^a^ of **3**, **4**, **5**, %			Yields^a^ of isolated **3**, **4**, **5**, %		

Ph	Ph	**2a**, 71	**3a**, 19	**4a**, 37	**5a**, 15	**3a**, 15	**4a**, 34	**5a**, 13
Ph	4-MeOC_6_H_4_	**2b**, 74	**3b**, 0	**4b**, 45	**5b**, 23	**–**	**4b**, 24	**5b**, 20
Ph	4-NO_2_C_6_H_4_	**2c**, 50	**3c**, 89	**4c**, 0	**5c**, 0	**3c**, 79	**–**	**–**
4-MeOC_6_H_4_	Ph	**2a**, 70	**3d**, 77	**4d**, 14	**5d**, 9	**3d**, 42	**4d** + **5d**, 18	
4-MeOC_6_H_4_	4-MeOC_6_H_4_	**2b**, 72	**3e**, 0	**4e**, 41	**5e**, 23	**–**	**4e** + **5e**, 51	
4-MeOC_6_H_4_	4-NO_2_C_6_H_4_	**2c**, 50	**3f**, 92	**4f**, 0	**5f**, 0	**3f**, 85	**–**	**–**
4-NO_2_C_6_H_4_	Ph	**2a**, 87	**3g**, 0	**4g**, 58	**5g**, 29	**–**	**4g** + **5g**, 80	
4-NO_2_C_6_H_4_	4-MeOC_6_H_4_	**2b**, –	**3h**, 0	**4h**, 0	**5h**, 0	**–**	**–**	**–**
4-NO_2_C_6_H_4_	4-NO_2_C_6_H_4_	**2c**, 50	**3i**, 62	**4i**, 0	**5i**, 0	**3i**, 56	**–**	**–**

^a^Yield based on consumed azirine.

Furandiones **1a–c** react with 3-(4-nitrophenyl)-2*H*-azirine (**2c**) to give only 1:2 adducts **3**. These were easily isolated from the reaction mixtures by crystallization. In reactions of **1a** with **2b**, **1b** with **2b**, and **1c** with **2a** only 2:2 adducts **4** and **5** are formed and were isolated by chromatography. Thermolysis of furandione **1c** in the presence of azirine **2b** led to tarring. Analysis of the data obtained (Table 2) shows that the ratio of the products **3–5** is determined by the electronic effects of the substituents in the benzene rings both in arylazirine **2** and arylfurandione **1**. An increase of the electron-withdrawing effect of substituents in the benzene rings of 3-aryl-2*H*-azirine leads to an increase of yield of 1:2 adduct **3**, and in the case of 3-(4-nitrophenyl)-2*H*-azirine (**2c**) it becomes the only product, while from 3-(4-methoxyphenyl)-2*H*-azirine (**2b**) only 2:2 adducts **4** and **5** were formed. It is also worth noting that in all cases the proportion of *cis*-isomer **4** was larger than that of *trans*-isomer **5**.

**Scheme 2:**
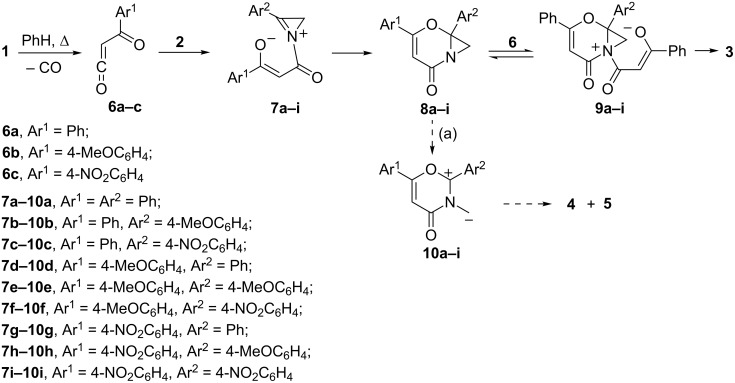
The route of formation of compounds **3** and possible intermediates in route to compounds **4** and **5**.

**Figure 2:**
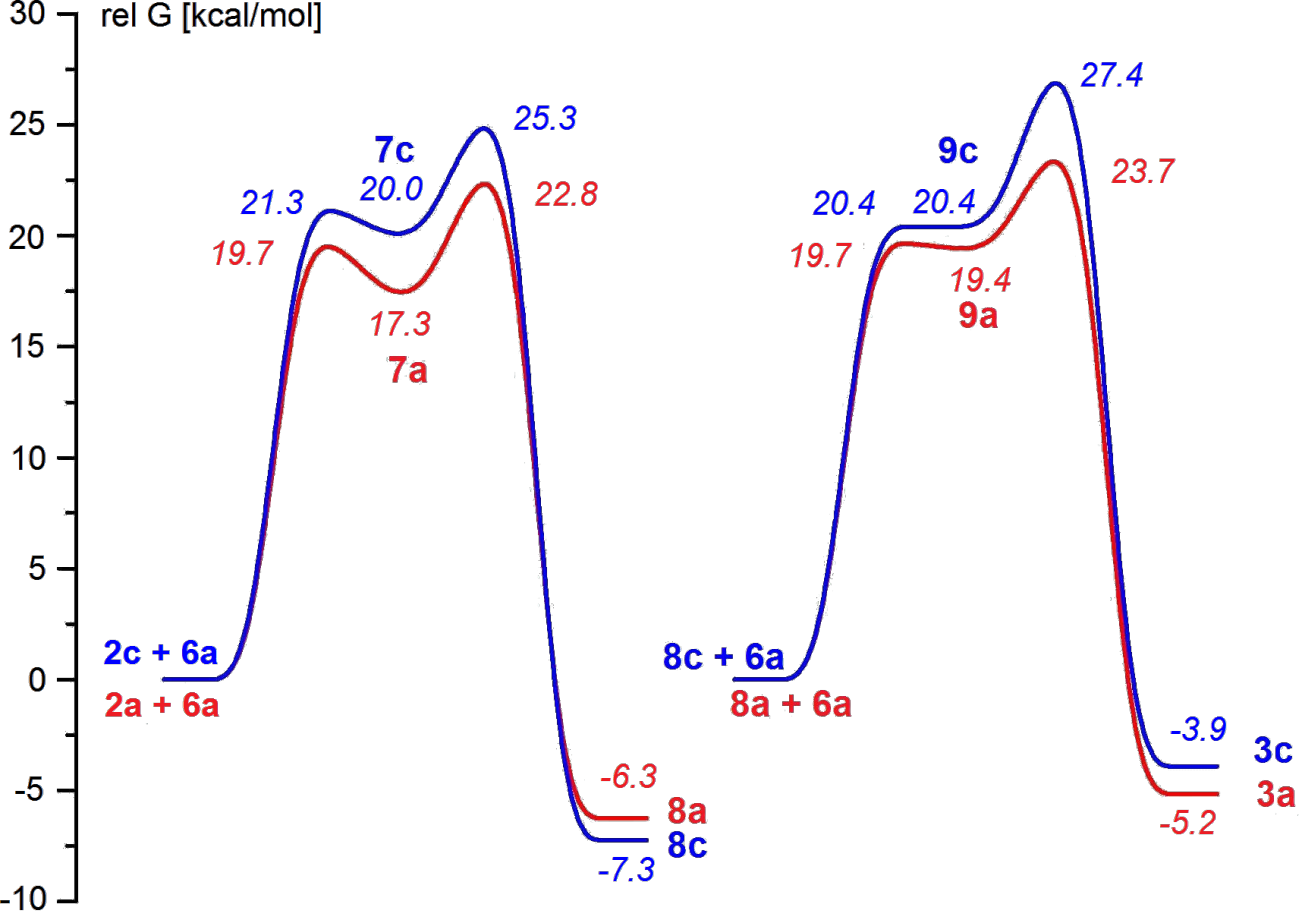
Energy profiles for the reactions of azirines **2a**,**c** and acylketene **6a**, as well as acylketene **6a** with monoadducts **8a**,**c**. Relative free energies [kcal·mol^−1^, 353 K, benzene (PCM)] computed at the DFT B3LYP/6-31G(d) level.

The formation of compounds **3** proceeds in the same way as for similar compounds obtained by reaction of azirines with acylketenes from diazo compounds (Scheme 2) [22]. According to the calculation at the DFT B3LYP/6-31G(d) level with PCM solvation model for benzene (Figure 2) the formation of the monoadducts **8a**,**c** proceeds via the formation of zwitterionic intermediates **7a**,**c** by nucleophilic attack of the azirine nitrogen lone pair on the C=O group of the ketene fragment of intermediate **6a**. Interaction of monoadducts **8a**,**c** with ketene **6a** leads to the formation of the unstable zwitterionic intermediates **9a**,**c** which further cyclize to bisadducts **3a**,**c**. The barriers for addition of the azirine and aziridine nitrogen lone pair of **1a**,**c**, and **8a**,**c** to ketene **6a** increase in passing from compounds **1a**, **8a** to compounds **1c**, **8c**, because of a decrease in the nucleophilicity of the latter due to the electron withdrawing effect of the nitro group.

As for possible routes for the formation of adducts **4** and **5**, the first (Scheme 2, (a)) involves cleavage of the aziridine ring of intermediate **8** to generate azomethine ylide **10**, and further “dimerization” of the latter. Examples of compounds that can be considered as dimers of azomethine ylides have been published, though concerted thermal dimerization of azomethine ylides is a forbidden process [33]. According to our calculations the free energy barriers to formation of the azomethine ylides **10a–c** from compounds **8a–c** are 34.1, 34.7, 32.2 kcal·mol^−1^ (353 K, benzene (PCM)), respectively, that far exceed the barriers to reactions leading to compound **3**. These do not allow the possibility that azomethine ylide **10** can be a probable intermediate in the formation of adducts **4** and **5**.

**Scheme 3:**
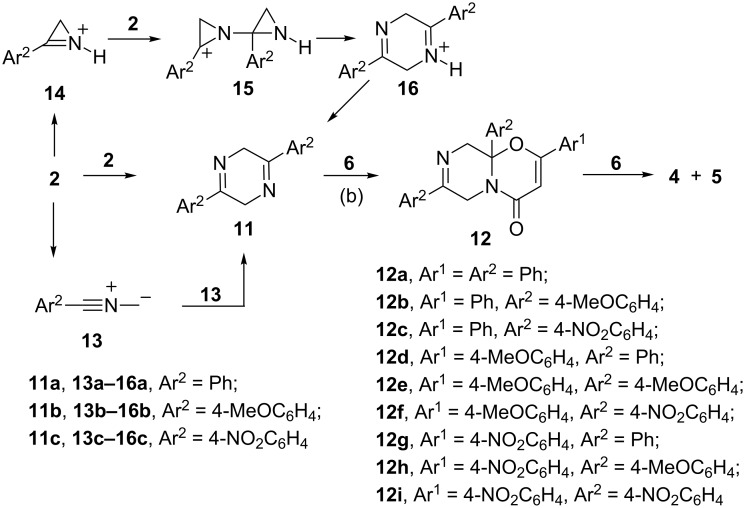
Possible intermediates in routes to compounds **4** and **5**.

It has been known that imines react with acylketenes, generated from furandiones, to give derivatives of 1,3-oxazines [34–36]. Another route to compounds **4** and **5** could, therefore, involve interaction of dihydropyrazine **11** with ketene **6**, leading to the monoadduct **12**, which further reacts with a second molecule of **6** to give 2:2 adducts **4** and **5** (Scheme 3, (b)).

**Figure 3:**
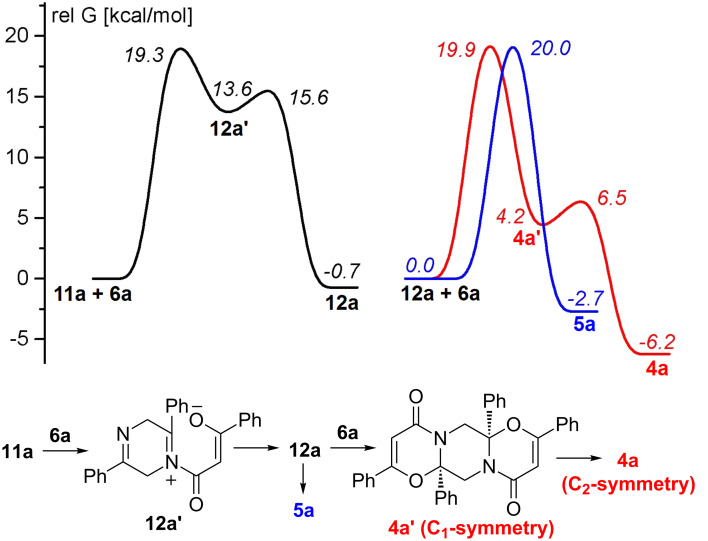
Energy profiles for the reactions of dihydropyrazine **11a** with acylketene **6a**, as well as acylketene **6a** with monoadduct **12a**. Relative free energies [kcal·mol^−1^, 353 K, benzene (PCM)] computed at the DFT B3LYP/6-31G(d) level.

To evaluate the free energy barriers for the interaction of 2,5-dihydropyrazine with acylketenes the calculations of the reaction of dihydropyrazine **11a** with ketene **6a**, leading to adduct **12a**, and the reaction of the latter with ketene **6a**, leading to adducts **4a** and **5a**, were performed at the DFT B3LYP/6-31G(d) level (Figure 3).

According to the calculation (Figure 3) the formation of monoadduct **12a** proceeds via the formation of the zwitterionic intermediate **12a'** by nucleophilic attack of the dihydropyrazine nitrogen lone pair on the C=O group of ketene **6a**. Intermediate **12a'** further easily undergoes cyclization to give monoadduct **12a**. Interaction of the latter with ketene **6a** leads to unsymmetrical *cis*-isomer **4a'** with the piperazine ring in a chair conformation. The isomer **4a'** transforms through a low barrier to a much more stable isomer **4a** of C_2_ symmetry with the piperazine ring in a boat conformation (see Supporting Information File 1). No intermediate structure was located on the way to the most stable conformation of *trans*-isomer **5a** with the piperazine ring in a boat conformation. The free energies of the highest transition states on the pathways from **12a** to *cis*-isomer **4a** and *trans*-isomer **5a** are practically equal, but **4a** is much more stable than **5a**. Therefore, one can consider the experimental **4a**:**5a** isomer ratio of 37:15 to result from the thermodynamic control, since the barrier to the back transformation of **5a** to **12a** + **6a** is as low as 22.7 kcal·mol^−1^. Calculations also show (Figure 2 and Figure 3) that the reaction involving dihydropyrazines **11** on the way to **4** and **5** could be quite competitive with the reaction leading to **3**, provided that a source of dihydropyrazines **11** is available. Formation of ‘dimer azirines’, dihydropyrazines [37–41], or products of their dehydrogenation, pyrazines [37–49] under different conditions is quite common. Moreover, everybody who works with 3-aryl-2*H*-azirines faces the problem of their storage, because these compounds, both with unsubstituted and an electron-donor substituted benzene ring, fast transform into pyrazines, even when stored in a fridge.

**Figure 4:**
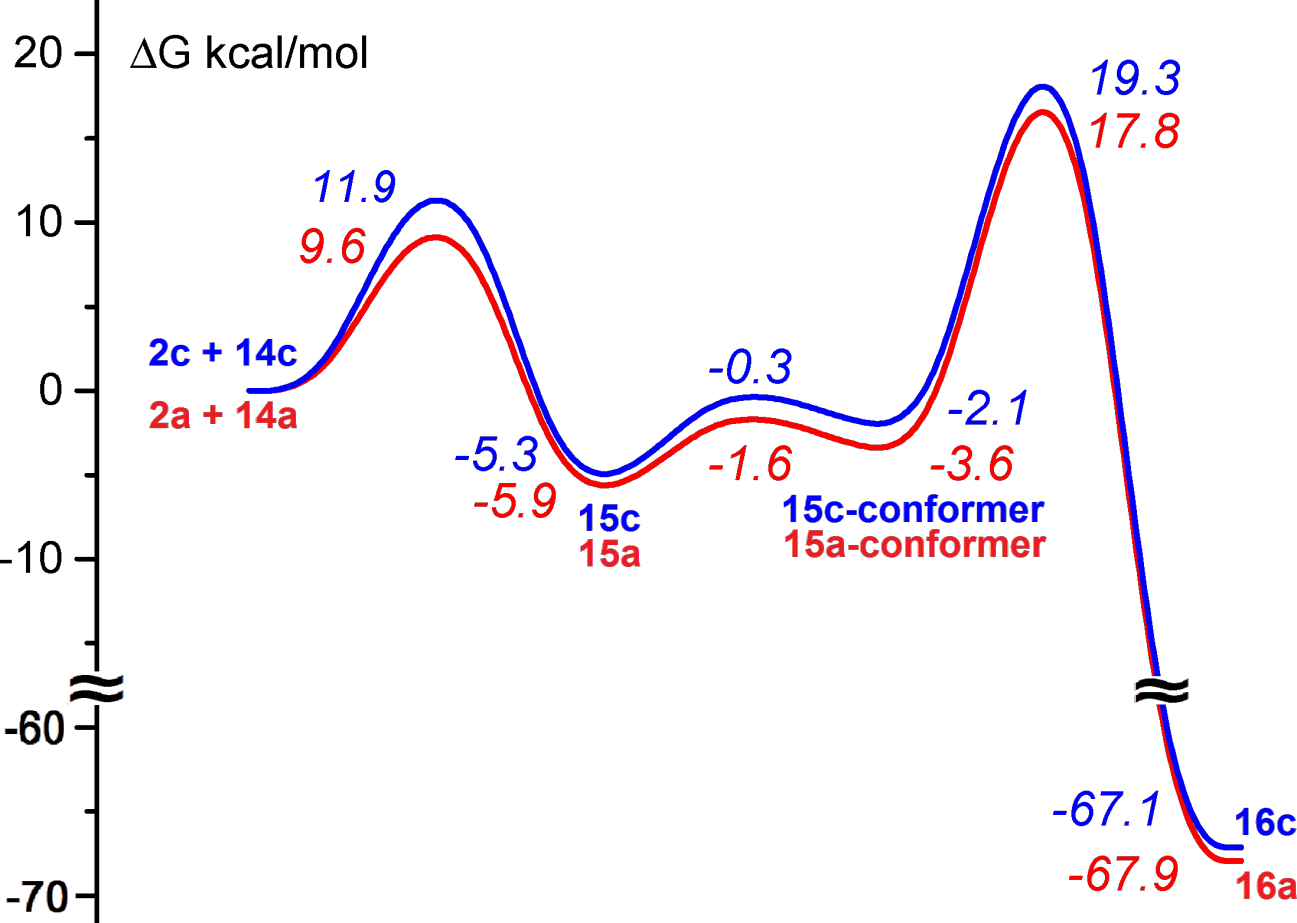
Energy profiles for the reactions of azirines **2a,c** with protonated azirines **14a,c**. Relative free energies [kcal·mol^−1^, 353 K, benzene (PCM)] computed at the DFT B3LYP/6-31G(d) level.

Different mechanisms of dimerization were assumed, such as formation and dimerization of nitrile ylides [37,40], hydrolysis to α-aminoketenes followed by condensation [37,41], intermediate formation of metal complexes in the reaction mediated by metals [41,43,46]. It was found that water [37], Brønsted [44,48] and Lewis acids [40–41,43] facilitate the formation of pyrazine derivatives. 2*H*-Azirines undergo ring opening on electronic excitation to give nitrile ylides [50]. Nitrile ylide formation under thermal conditions even from such strained compounds as 2*H*-azirines needs to overcome a quite high energy barrier. According to calculations at the DFT B3LYP/6-31G(d) level the free energy barriers to formation of nitrile ylides **13a–c** from azirines **2a–c** are 48.4, 47.6, 47.9 kcal·mol^−1^ (353 K, benzene (PCM)), respectively. Therefore the process of the formation of dihydropyrazines **11** via azirine–nitrile ylide isomerization cannot compete with reaction of azirines with acylketenes (Figure 2). Dimerization of azirine **2a** via nucleophilic attack of the nitrogen lone pair of one azirine molecule on the C=N bond of another is also energetically unfavourable (ΔG^#^ = 53.6 kcal·mol^−1^, 353 K, benzene (PCM)). In contrast to this, the nucleophilic attack of the nitrogen lone pair of azirine **2** on the C=N bond of protonated azirine **14** and consequent cyclization to dihydropyrazine **15** proceeds via quite low barriers (Figure 4).

**Scheme 4:**
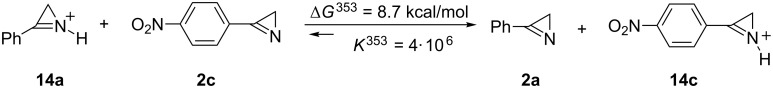
Isodesmic equation for evaluation of relative basicity of azirines **2c**,**a**.

By comparison of the data presented on Figures 2–4 one can conclude that competitive formation of compounds **3**, **4** and **5** can proceed under acidic catalysis. Probably traces of water cause hydrolysis of the furandiones **1a–c** to give 4-aryl-2,4-dioxobutanoic acids, which can protonate basic azirines **2a,b**. The concentration of protonated azirine **2c** have to be negligible due to low basicity of this azirine, as one can see from isodesmic equation (Scheme 4).

Thus, the absence of **4c**,**f**,**i** and **5c**,**f**,**i** in the reaction of furandiones **1a–c** with azirine **2c** can most probably be explained by the low basicity of the latter, and this prevents the formation of **11c** in any significant concentration.

We also decided to implement this theoretical conclusion into an approach to storing 3-aryl-2*H*-azirines. It was found that a sample of azirine **2a** upon storage over anhydrous K_2_CO_3_ at room temperature for 2 months underwent no changes, whereas a sample stored under the same conditions but without addition of K_2_CO_3_ completely transformed into 2,5-diphenylpyrazine.

**Scheme 5:**
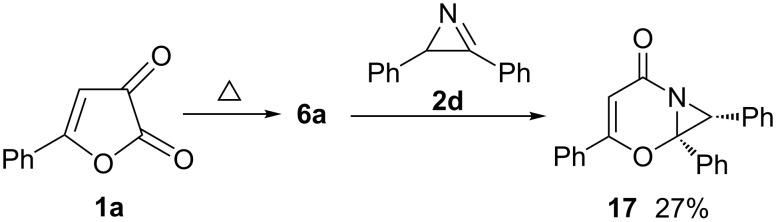
Reaction of furandione **1a** with azirine **2d**.

**Figure 5:**
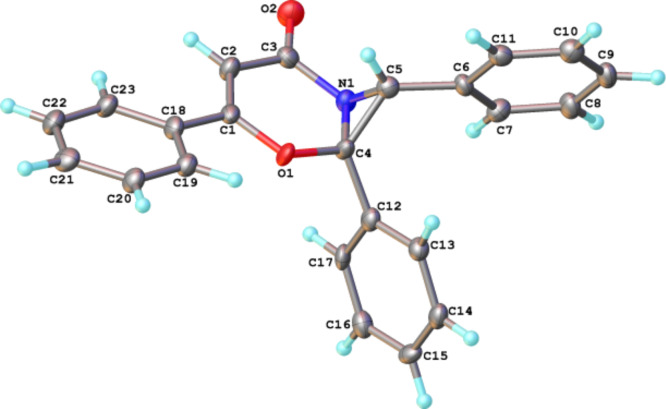
Molecular structure of compound **17**.

The reaction of 2-phenyl-substituted azirine **2d** with furandione **1a** leads, obviously due to steric reasons, to formation of only the *exo*-monoadduct **17** (Scheme 5). The structure of compound **17** was confirmed by X-ray analysis (Figure 5). In the case of the reaction of the azirine **2d** “dimeric” products of type **3**, **4** and **5** were not detected, most probably due to steric hindrance both for the reaction of monoadduct **17** with acylketene **3a** and the “dimerization” of **2d** to tetraphenyldihydropyrazole.

**Scheme 6:**
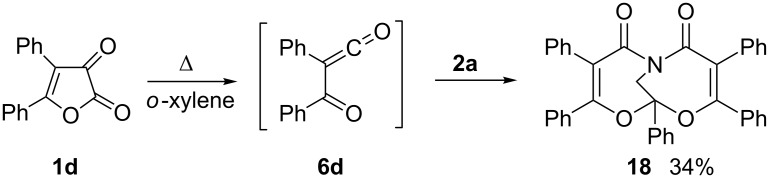
Reaction of furandione **1d** with azirine **2a**.

4,5-Diphenylfuran-2,3-dione (**1d**) is the source of benzoylphenylketene **6d**. Reaction of ketene **6d**, generated from 2-diazo-1,3-diphenylpropane-1,3-dione, with azirine **2a** was studied earlier [22]. Higher temperatures are needed to generate benzoylphenylketene **6d** from furanedione **1d**, than from the diazo compound. Boiling an *o*-xylene solution of furanedione **1d** and azirine **2a** (1:1 ratio) gave bisadduct **18** in 34% yield (Scheme 6). This is less than when using the diazo compound as a source of acylketene, probably due to dimerization of ketene **6d** under higher temperature.

**Scheme 7:**
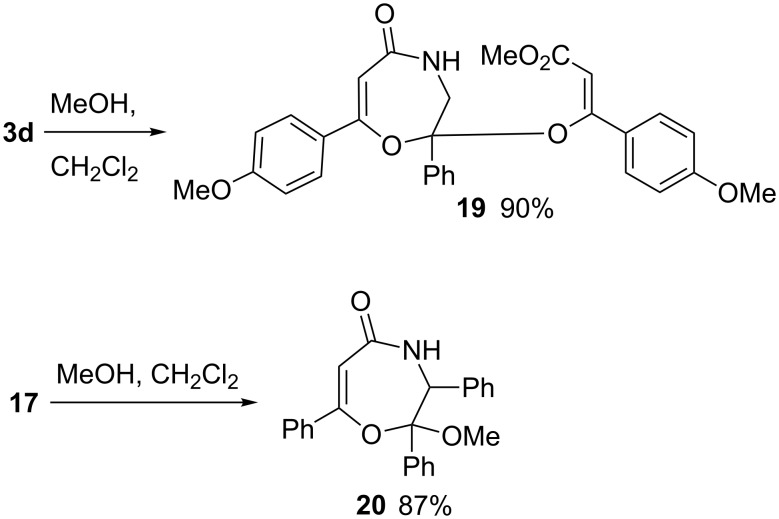
Reactions of compounds **3d** and **18a** with methanol.

Compounds **3** and **17**, stable at room temperature, react with methanol under mild conditions. Thus the boiling of methanol/CH_2_Cl_2_ (1:2) solutions of compound **3d** and **17** leads to the formation of the corresponding derivatives of 3,4-dihydro-1,4-oxazepin-5(2*H*)-one **19** and **20** (Scheme 7).

## Conclusion

2-Unsubstituted 3-aryl-2*H*-azirines **2** react with acylketenes, generated by thermolysis of 5-arylfuran-2,3-diones **1**, to give 5,7-dioxa-1-azabicyclo[4.4.1]undeca-3,8-diene-2,10-diones **3** and/or *cis*- and *trans*-6,6a,12,12a-tetrahydrobis[1,3]oxazino[3,2-*a*:3′,2′-*d*]pyrazine-4,10-diones **4** and **5**. The latter compounds are the products of coupling of two molecules of azirine with two molecules of acylketene. The ratio of the adducts **3–5** is determined by electronic effects of the substituents in the benzene rings both in arylazirine **2** and arylfurandione **1**. The increase of the electron-withdrawing effect of the substituents in the benzene rings of the arylazirine leads to an increase in the yield of 1:2 adduct **3**, and in the case of 3-(4-nitrophenyl)-2*H*-azirine (**2c**) it becomes the only product, while from 3-(4-methoxyphenyl)-2*H*-azirine (**2b**) only 2:2 adducts **4** and **5** were formed. Calculations at the DFT B3LYP/6-31G(d) level for various routes of bis[1,3]oxazino[3,2-*a*:3′,2′-*d*]pyrazine skeleton formation revealed a new reaction of 3-aryl-2*H*-azirines in the presence of acylketenes from furandiones, i.e. acid-catalyzed dimerization to dihydropyrazines followed by consecutive double cycloaddition of the latter to acylketenes. According to the calculations the larger proportion of *cis*-isomer **4** than of *trans*-isomer **5** is a result of thermodynamic control. We also recommend storing liquid 3-aryl-2*H*-azirines, both with unsubstituted and an electron-donor substituted benzene ring, over anhydrous K_2_CO_3_.

## Experimental

### General methods

Melting points were determined on a hot stage microscope and are uncorrected. ^1^H (300 MHz) and ^13^C (75 MHz) NMR spectra were determined in CDCl_3_ with a Bruker DPX 300 spectrometer. Chemical shifts (δ) are reported in parts per million downfield from tetramethylsilane. Electrospray ionization mass spectra were measured on MS Q-TOF and micrOTOF 10223 mass spectrometers. IR spectra were recorded on a Bruker TENSOR 27 spectrometer for tablets in KBr. Single-crystal X-ray data for **3a** were collected at 100 K on a Bruker Proteum R diffractometer (FR-591 rotating anode generator, Pt-135 CCD detector) equipped with Cobra (Oxford Cryosystems) open-flow cryostat. Data for **4b** and **17** were collected on an Agilent XCalibur diffractometer at the temperature 120 K maintained by Cryostream (Oxford Cryosystems) cryostat. The structures were solved by direct method and refined by full-matrix least squares on F^2^ for all data using Olex2 [51] and SHELXTL [52] software. All non-hydrogen atoms were refined anisotropically, hydrogen atoms in the structure **3a** were placed in the calculated positions and refined in riding mode. The hydrogen atoms in the structures **4b** and **17** were located in the difference Fourier maps and refined isotropically. Crystallographic data for the structure have been deposited with the Cambridge Crystallographic Data Centre as supplementary publication CCDC-974303-974305. Compounds **1a** [53], **1b,c** [32], **1d** [54], and **2a,b** [55], **2c** [56], **2d** [57] were prepared by the reported procedures.

**General procedures for reactions of acylketenes from 5-arylfuran-2,3-diones 2a–c and 3-aryl-2*H*-azirines 1a–c.** A mixture of azirine **1** (1 mmol) and furane-2,3-dione **2** (1 mmol) in anhydrous benzene (5 mL) was refluxed for 0.5–1 h. The solvent was removed in vacuum, and the residue was purified by flash chromatography on silica (eluent petroleum ether/ethyl acetate, 1:1).

**4,6,8-Triphenyl-5,7-dioxa-1-azabicyclo[4.4.1]undeca-3,8-diene-2,10-dione (3a)**. White solid; mp 214–215 °C (benzene); yield 15% (on consumed azirine); ^1^H NMR (CDCl_3_) δ 4.70 (s, 2H), 6.28 (s, 2H), 7.35–7.48 (m, 9H), 7.60–7.63 (m, 6H); ^13^C NMR (CDCl_3_) δ 49.1, 104.5, 113.6, 125.0, 127.0, 128.7, 129.0, 130.3, 131.1, 134.5, 137.4, 160.2, 164.9; IR (KBr, cm^−1^) *ν*: 1721 (C=O); HRMS–ESI: [M + Na]^+^ calcd for C_26_H_19_NNaO_4_^+^, 432.1206; found, 432.1192; Anal. calcd for C_26_H_19_NO_4_: C, 76.27; H, 4.68; N, 3.42; found: C, 76.57; H, 4.47; N, 3.66; Crystal data for **3a**: C_26_H_19_NO_4_, *M* = 409.42, monoclinic, space group *P* 2_1_/*n*, *a* = 14.5600(5), *b* = 17.9642(6), *c* = 17.1799(6) Å, β = 105.850(10)°, *U* = 4322.7(3) Å^3^, F(000) = 1712, *Z* = 8, *D*_c_ = 1.258 mg m^−3^, μ = 0.692 mm^−1^. 21195 reflections were collected yielding 5904 unique data (R_merg_ = 0.0506). Final wR_2_(F^2^) = 0.1073 for all data (559 refined parameters), conventional R_1_(F) = 0.0440 for 4157 reflections with I ≥ 2σ, GOF = 0.991.

**(6a*RS*,12a*RS*)-2,6a,8,12a-Tetraphenyl-6,6a,1,2,12a-tetrahydrobis[1,3]oxazino[3,2-*a*:3’,2’-*d*]pyrazine-4,10-dione (4a).** White solid; mp 154–156 °C (EtOAc/hexane); yield 34% (on consumed azirine); ^1^H NMR (CDCl_3_) δ 3.26 (d, *J* = 15.3 Hz, 2H), 5.62 (d, *J* = 15.3 Hz, 2H), 5.90 (s, 2H), 7.36–7.44 (m, 12H), 7.51–7.54 (m, 4H), 7.70–7.74 (m, 4H); ^13^C NMR (CDCl_3_) δ 47.9, 91.7, 98.7, 126.0, 126.3, 128.4, 128.7, 129.6, 131.3, 131.5, 138.4, 161.5, 163.2; IR (KBr, cm^−1^) *ν* 1674 (C=O); HRMS–ESI: [M + H]^+^ calcd for C_34_H_27_N_2_O_4_^+^, 527.1965; found, 527.1937.

**(6a*RS*,12a*SR*)-2,6a,8,12a-Tetraphenyl-6,6a,1,2,12a-tetrahydrobis[1,3]oxazino[3,2-*a*:3′,2′-*d*]pyrazine-4,10-dione (5a).** White solid; mp 171–173 °C (EtOAc/hexane); yield 13% (on consumed azirine); ^1^H NMR (CDCl_3_) δ 4.56 (d, *J* = 14.5 Hz, 2H), 4.68 (d, *J* = 14.5 Hz, 2H), 5.73 (s, 2H), 7.29–7.31 (m, 2H), 7.39–7.52 (m, 9H), 7.63–7.71 (m, 9H); ^13^C NMR (CDCl_3_) δ 47.5, 93.2, 97.6, 125.1, 126.3, 128.6, 129.0, 129.7, 131.3, 138.0, 162.3, 163.1; IR (KBr, cm^−1^) *ν*: 2930, 1661 (C=O); HRMS–ESI: [M + K]^+^ calcd for C_34_H_26_N_2_KO_4_^+^, 565.1524; found, 565.1496.

**(6a*RS*,12a*RS*)-6a,12a-Bis(4-methoxyphenyl)-2,8-diphenyl-6,6a,12,12a-tetrahydrobis[1,3]oxazino[3,2-*a*:3′,2′-*d*]pyrazine-4,10-dione (4b).** White solid; mp 186–186.5 °C (EtOAc/hexane); yield 24% (on consumed azirine); ^1^H NMR (CDCl_3_) δ 3.22 (d, *J* = 14.9 Hz, 2H), 3.76 (s, 6H), 5.59 (d, *J* = 14.9 Hz, 2H), 5.89 (s, 2H), 6.87 (d, *J* = 8 Hz, 4H), 7.34–7.45 (m, 10H), 7.69 (d, *J* = 8 Hz, 4H); ^13^C NMR (CDCl_3_) δ 47.6, 55.2, 93.1, 97.5, 114.2, 126.3, 126.5, 128.5, 129.8, 131.2, 131.4, 160.5, 162.1, 163.1; IR (KBr, cm^−1^) *ν*: 1731 (C=O); HRMS–ESI: [M + H]^+^ calcd for C_36_H_31_N_2_O_6_^+^, 587.2177; found, 587.2183; Crystal data for **4b**: C_36_H_30_N_2_O_6_, *M* = 586.62, monoclinic, space group *P* 2_1_/*c*, *a* = 12.1236(5), *b* = 18.2526(7), *c* = 13.4793(5) Å, β = 103.738(4)°, *U* = 2897.46(19) Å^3^, F(000) = 1232, *Z* = 4, *D*_c_ = 1.345 mg m^−3^, μ = 0.092 mm^−1^. 16575 reflections were collected yielding 6654 unique data (R_merg_ = 0.0596). Final wR_2_(F^2^) = 0.1284 for all data (517 refined parameters), conventional R_1_(F) = 0.0567 for 4337 reflections with I ≥ 2σ, GOF = 1.043.

**(6a*RS*,12a*SR*)-6a,12a-Bis(4-methoxyphenyl)-2,8-diphenyl-6,6a,12,12a-tetrahydrobis[1,3]oxazino[3,2-*a*:3′,2′-*d*]pyrazine-4,10-dione (5b).** White solid; mp 123–124 °C (EtOAc/hexane); yield 20% (on consumed azirine); ^1^H NMR (CDCl_3_) δ 3.71 (s, 6H), 4.53 (d, *J* = 14.2 Hz, 2H), 4.62 (d, *J* = 14.2 Hz, 2H), 5.74 (s, 2H), 6.75–6.78 (m, 4H), 7.40–7.48 (m, 6H), 7.53–7.56 (m, 4H), 7.67–7.69 (m, 4H); ^13^C NMR (CDCl_3_) δ 47.9, 55.1, 91.6, 98.6, 113.7, 126.3, 127.4, 128.7, 130.2, 131.36, 131.42, 160.3, 161.3, 163.3; IR (KBr, cm^−1^) *ν*: 1733 (C=O); HRMS–ESI: [M + H]^+^ calcd for C_36_H_31_N_2_O_6_^+^, 587.2177; found, 587.2196.

**Calculations.** All calculations were carried out at the DFT B3LYP/6-31G(d) level [58–60] by using the Gaussian 09 suite of quantum chemical programs [61] at Resource center ‘Computer center of Saint Petersburg State University’. Geometry optimizations of intermediates, transition states, reactants, and products in benzene were performed using the PCM model. Intrinsic reaction coordinates were calculated to authenticate all transition states.

## Supporting Information

Detailed experimental procedures including characterization data for all synthesized compounds, ^1^H and ^13^C NMR spectra for all new compounds. Computational details: energies of molecules, transition states and their Cartesian coordinates of atoms.

File 1 Detailed experimental procedures and computational details.

File 2 Chemical information file of compound **3a**.

File 3 Chemical information file of compound **4b**.

File 4 Chemical information file of compound **17**.

## References

[1] Khlebnikov A F, Novikov M S (2013). Tetrahedron.

[2] Padwa A (2010). Adv Heterocycl Chem.

[3] Lemos A (2009). Molecules.

[4] Padwa A, Katritzky A R, Ramsden C A, Scriven E F V (2008). Comprehensive Heterocyclic Chemistry III.

[5] Pinho e Melo T M V D, d’A. Rocha Gonsalves A M (2004). Curr Org Synth.

[6] Palacios F, Ochoa de Retana A M, Martínez de Marigorta E, de los Santos J M (2002). Org Prep Proced Int.

[7] Palacios F, Ochoa de Retana A M, Martínez de Marigorta E, de los Santos J M (2001). Eur J Org Chem.

[8] Gilchrist T L (2001). Aldrichimica Acta.

[9] Rai K L M, Hassner A, Halton B (2000). Advances in Strained and interesting Organic Molecules.

[10] Cardoso A L, Gimeno L, Lemos A, Palacios F, Pinho e Melo T M V D (2013). J Org Chem.

[11] Nunes C M, Reva I, Fausto R (2013). J Org Chem.

[12] Januar L A, Molinski T F (2013). Org Lett.

[13] Loy N S Y, Singh A, Xu X, Park C-M (2013). Angew Chem, Int Ed.

[14] Rostovskii N V, Novikov M S, Khlebnikov A F, Korneev S M, Yufit D S (2013). Org Biomol Chem.

[15] Banert K, Ihle A, Kuhtz A, Penk E, Saha B, Würthwein E-U (2013). Tetrahedron.

[16] Zavyalov K V, Novikov M S, Khlebnikov A F, Yufit D S (2013). Tetrahedron.

[17] Rostovskii N V, Novikov M S, Khlebnikov A F, Khlebnikov V A, Korneev S M (2013). Tetrahedron.

[18] Duarte V C M, Faustino H, Alves M J, Gil Fortes A, Micaelo N (2013). Tetrahedron: Asymmetry.

[19] Zheng Y, Yang C, Zhang-Negrerie D, Du Y, Zhao K (2013). Tetrahedron Lett.

[20] Banert K, Bochmann S, Hagedorn M, Richter F (2013). Tetrahedron Lett.

[21] Räber J L, Stoykova S A, Strässler C, Heimgartner H (2013). Phosphorus, Sulfur Silicon Relat Elem.

[22] Khlebnikov A F, Novikov M S, Pakalnis V V, Yufit D S (2011). J Org Chem.

[23] Kascheres A, Nunes J, Brandão F (1997). Tetrahedron.

[24] Schaumann E, Grabley S, Henriet M, Ghosez L, Touillaux R, Declercq J P, Germain G, Van Meerssche M (1980). J Org Chem.

[25] Haddadin M J, Hassner A (1973). J Org Chem.

[26] Hassner A, Miller A S, Haddadin M J (1972). Tetrahedron Lett.

[27] Allen A D, Tidwell T T (2013). Chem Rev.

[28] Paull D H, Weatherwax A, Lectka T (2009). Tetrahedron.

[29] Kirmse W (2002). Eur J Org Chem.

[30] Reber K P, Tilley S D, Sorensen E J (2009). Chem Soc Rev.

[31] Wentrup C, Heilmayer W, Kollenz G (1994). Synthesis.

[32] Murai S, Hasegawa K, Sonoda N (1975). Angew Chem, Int Ed Engl.

[33] Freeman F, Govindarajoo G (1995). Rev Heteroat Chem.

[34] Andreichikov Yu S, Nekrasov D D, Rudenko M A, Konovalov A Yu (1987). Chem Heterocycl Compd.

[35] Andreichikov Y S, Ionov Y V (1981). J Org Chem USSR.

[36] Ziegler E, Kollenz G, Ott W (1973). Synthesis.

[37] Banert K, Meier B (2006). Angew Chem, Int Ed.

[38] Alves M J, Gilchrist T L (1998). J Chem Soc, Perkin Trans 1.

[39] Inada A, Heimgartner H (1982). Helv Chim Acta.

[40] Alper H, Prickett J E, Wollowitz S (1977). J Am Chem Soc.

[41] Alper H, Wollowitz S (1975). J Am Chem Soc.

[42] Palacios F, Ochoa de Retana A M, Gil J I, de Munain R L (2002). Org Lett.

[43] Auricchio S, Grassi S, Malpezzi L, Sartori A S, Truscello A M (2001). Eur J Org Chem.

[44] Flammang R, Lacombe S, Laurent A, Maquestiau A, Marquet B, Novkova S (1986). Tetrahedron.

[45] Nitta M, Kobayashi T (1984). Bull Chem Soc Jpn.

[46] Nitta M, Kobayashi T (1983). Chem Lett.

[47] Hassner A, Belinka B A, Steinfeld A S (1982). Heterocycles.

[48] Alvernhe G, Lacombe S, Laurent A (1980). Tetrahedron Lett.

[49] Smolinsky G (1961). J Am Chem Soc.

[50] Padwa A (1976). Acc Chem Res.

[51] Dolomanov O V, Bourhis L J, Gildea R J, Howard J A K, Puschmann H (2009). J Appl Crystallogr.

[52] Sheldrick G M (2008). Acta Crystallogr, Sect A.

[53] Sof'ina O A, Igidov N M, Koz'minykh E N, Trapeznikova N N, Kasatkina Yu S, Koz'minykh V O (2001). Russ J Org Chem.

[54] Vostrov E S, Leont'eva E V, Tarasova O P, Maslivets A N (2003). Russ J Org Chem.

[55] Hortmann A G, Robertson D A, Gillard B K (1972). J Org Chem.

[56] Brown D, Brown G A, Andrews M, Large J M, Urban D, Butts C P, Hales N J, Gallagher T (2002). J Chem Soc, Perkin Trans 1.

[57] Fowler F W, Hassner A, Levy L A (1967). J Am Chem Soc.

[58] Becke A D (1993). J Chem Phys.

[59] Becke A D (1988). Phys Rev A.

[60] Lee C, Yang W, Parr R G (1988). Phys Rev B.

[61] (2010). Gaussian 09.

