# Supplementary material for: Domino reactions of 2H-azirines with acylketenes from furan-2,3-diones: Competition between the formation of ortho-fused and bridged heterocyclic systems
Source: Beilstein J Org Chem. 2014 Apr 4;10:784–93. doi: 10.3762/bjoc.10.74 (PMC3999847; doi:10.3762/bjoc.10.74)
Supplement: File 1 — Detailed experimental procedures and computational details. [file Beilstein_J_Org_Chem-10-784-s001.pdf]

Supporting Information

for

Domino reactions of 2*H*-azirines with acylketenes from furan-2,3-diones: Competition between the formation of *ortho*-fused and bridged heterocyclic systems

Alexander F. Khlebnikov*[1], Mikhail S. Novikov[1], Viktoriia V. Pakalnis[1], Roman O. Iakovenko[1] and Dmitry S. Yufit[2]

Address: [1]Department of Chemistry, Saint-Petersburg State University, Universitetskii pr. 26, 198504 St. Petersburg, Russia and [2]Department of Chemistry, University of Durham, Durham, South Rd., DH1 3LE, UK

Email: Alexander F. Khlebnikov - alexander.khlebnikov@pobox.spbu.ru

*Corresponding author

This article is dedicated to Professor Armin de Meijere on the occasion of his 75th birthday.

Detailed experimental procedures and computational details.

Pages S2–S4: Detailed experimental procedures including characterization data for all sythesized compounds.
Pages S5–S38: ^{1}H and ^{13}C NMR spectra of new compounds.
Pages S39–S63: Computational details.

General procedures for reactions of acylketenes from 5-arylfuran-2,3-diones 2a–c and 3-aryl-2*H*-azirines 1a–c. A mixture of azirine **1** (1 mmol) and furane-2,3-dione **2** (1 mmol) in anhydrous benzene (5 mL) was refluxed for 0.5–1 h. The solvent was removed in vacuum, and the residue was purified by flash chromatography on silica (eluent petroleum ether/ethyl acetate, 1:1).

6-(4-Nitrophenyl)-4,8-diphenyl-5,7-dioxa-1-azabicyclo[4.4.1]undeca-3,8-diene-2,10-dione (3c). White solid; mp 234–236 °C (benzene); yield 79% (on consumed azirine); ^{1}H NMR ($CDCl_3$) δ 4.73 s (2H), 6.31 s (2H), 7.38–7.43 m (4H), 7.46–7.51 m (2H), 7.60 d (4H, J = 8 Hz), 7.79 d (2H, J = 8 Hz), 8.22 d (2H, J = 8 Hz); ^{13}C NMR ($CDCl_3$) δ 48.7, 105.0, 112.4, 124.1, 126.5, 126.7, 128.9, 131.5, 133.9, 143.6, 159.7, 164.3; IR (KBr, cm^{-1}) ν 1709 (C=O); HRMS-ESI calcd for $C_{26}H_{19}N_2O_6^+$ $[M+H]^+$ 455.1238, found 455.1246.

4,8-Bis(4-methoxyphenyl)-6-phenyl-5,7-dioxa-1-azabicyclo[4.4.1]undeca-3,8-diene-2,10-dione (3d). White solid; mp 202–203 °C (benzene); yield 42% (on consumed azirine); ^{1}H NMR ($CDCl_3$) δ 3.85 s (6H), 4.65 s (2H), 6.18 s (2H), 6.87 d (4H, J = 9.1 Hz), 7.38–7.41 m (3H), 7.56–7.62 m (6H); ^{13}C NMR ($CDCl_3$) δ 49.5, 55.4, 102.6, 113.2, 114.0, 125.0, 126.7, 128.8, 128.9, 130.1, 137.7, 160.0, 161.9, 165.2; IR (KBr, cm^{-1}) ν 1711 (C=O); HRMS-ESI calcd for $C_{28}H_{24}NO_6^+$ $[M+H]^+$ 470.1598, found 470.1574.

(6a*RS*,12a*RS*)-2,8-Bis(4-methoxyphenyl)-6a,12a-diphenyl-6,6a,12,12a-tetrahydrobis[1,3]oxazino[3,2-*a*:3′,2′-*d*]pyrazine-4,10-dione (4d), (6a*RS*,12a*SR*)-2,8-bis(4-methoxyphenyl)-6a,12a-diphenyl-6,6a,12,12a-tetrahydrobis[1,3]oxazino[3,2-*a*:3′,2′-*d*]pyrazine-4,10-dione (5d), (4d/5d* mixture 3.7/1). White solid; mp 185–189 °C (CH_2Cl_2/hexane); yield 18% (on consumed azirine); ^{1}H NMR ($CDCl_3$) δ 3.24 d (7.4H, J = 14.9 Hz), 3.83 s (22.2H), 3.87* s (6H), 4.53* d (2H, J_{AB} = 14.5 Hz), 4.66* d (2H, J_{AB} = 14.2 Hz), 5.59 d (7.4H, J = 15.2 Hz), 5.62* s (2H), 5.78 s (7.4H), 6.89 d (14.8H, J = 9 Hz), 6.94* d (4H, J = 9 Hz), 7.36–7.38 m (24H), 7.49–7.52 m (16H), 7.61–7.69 m (24.8H); ^{13}C NMR ($CDCl_3$) δ 48.0, 48.4*, 55.81, 55.83*, 92.0*, 93.5, 96.4, 97.4*, 114.4, 114.6*, 124.1*, 124.2, 125.5, 126.4*, 128.5*, 128.6, 128.8*, 129.3, 129.8*, 130.0, 138.7, 139.1*, 161.9*, 162.6, 162.7, 162.8*, 163.8, 163.9*; IR (KBr, cm^{-1}) ν 1671, 1653 (C=O); HRMS-ESI calcd for $C_{36}H_{31}N_2O_6^+$ $[M+H]^+$ 587.2177, found 587.2169.
Compound 4d. White solid; mp 198–200 °C (EtOAc/hexane); ^{1}H NMR ($CDCl_3$) δ 3.24 d (2H, J = 15.2 Hz), 3.83 s (6H), 5.59 d (2H, J = 15.2 Hz), 5.78 s (2H, 2C=CH), 6.89 d (4H, J = 9 Hz), 7.36–7.38 m (6H), 7.49–7.51 m (4H), 7.67 d (4H, J = 9 Hz); ^{13}C NMR ($CDCl_3$) δ 47.6, 55.4, 93.1, 96.0, 114.0, 123.9, 125.1, 128.2, 128.9, 129.6, 138.3, 162.2, 162.3, 163.4; IR (KBr, cm^{-1}) ν 1660 (C=O); HRMS-ESI calcd for $C_{36}H_{30}N_2NaO_6^+$ $[M+Na]^+$ 609.1996, found 609.2024.

(6a*RS*,12a*RS*)-2,6a,8,12a-Tetrakis(4-methoxyphenyl)-6,6a,12,12a-tetrahydrobis[1,3]oxazino[3,2-*a*:3′,2′-*d*]pyrazine-4,10-dione (4e), (6a*RS*,12a*SR*)-2,6a,8,12a-Tetrakis(4-methoxyphenyl)-6,6a,12,12a-tetrahydrobis[1,3]oxazino[3,2-*a*:3′,2′-*d*]pyrazine-4,10-dione (5e). Overall yield 51% (on consumed azirine).

Compound 4e. White solid; mp 180–181 °C (EtOAc/hexane); ^{1}H NMR ($CDCl_3$) δ 3.19 d (2H, J = 14.9 Hz), 3.77 s (6H), 3.82 s (6H), 3.72 s (6H), 3.86 s (6H), 5.55 d (2H, J = 14.9 Hz), 5.77 s (2H), 6.85–6.89 m (8H), 7.42 d (4H, J = 8.7 Hz), 7.65 d (4H, J = 8.7 Hz); ^{13}C NMR ($CDCl_3$) δ 47.7, 55.3, 55.4, 93.0, 95.9, 114.0, 114.2, 123.9, 126.5, 128.1, 130.1, 160.5, 162.1, 162.2, 163.5; IR (KBr, cm^{-1}) ν 1658 (C=O); HRMS-ESI calcd for $C_{38}H_{34}N_2NaO_8^+$ $[M+Na]^+$ 669.2207, found 669.2225.

Compound 5e. White solid; mp 174–175 °C (EtOAc/hexane); ^{1}H NMR ($CDCl_3$) δ 3.72 s (6H), 3.86 s (6H), 4.50 d (2H, J = 14.5 Hz), 4.59 d (2H, J = 14.2 Hz), 5.63 s (2H), 6.76 d (4H, J = 9 Hz), 6.93 d (4H, J = 9 Hz), 7.53 d (4H, J = 9 Hz), 7.64 d (4H, J = 9 Hz); ^{13}C NMR ($CDCl_3$) δ 48.0, 55.2, 55.4, 91.5, 97.0, 113.7, 114.1, 123.9, 127.4, 128.1, 130.5, 160.3, 161.3, 162.3, 163.6; IR (KBr, cm^{-1}) ν 1653 (C=O); HRMS-ESI calcd for $C_{38}H_{35}N_2O_8^+$ [M+H]$^+$ 647.2388, found 647.2389.

4,8-Bis(4-methoxyphenyl)-6-(4-nitrophenyl)-5,7-dioxa-1-azabicyclo[4.4.1]undeca-3,8-diene-2,10-dione (3f). White solid; mp 233–235 °C ($CHCl_3/Et_2O$); yield 85% (on consumed azirine); ^{1}H NMR (DMSO-d_6) δ 3.77 s (6H), 5.06 s (2H), 6.27 s (2H), 6.93 d (4H, J = 8.0 Hz), 7.61 d (4H, J = 8.3 Hz), 8.07 d (2H, J = 8.3 Hz), 8.26 d (2H, J = 8.3 Hz); ^{13}C NMR (DMSO-d_6) δ 47.8, 56.3, 104.6, 112.7, 115.0, 124.7, 126.8, 128.0, 129.3, 144.4, 149.1, 158.5, 162.3, 165.5; IR (KBr, cm^{-1}) ν 1703 (C=O); HRMS-ESI calcd for $C_{28}H_{22}N_2NaO_8^+$ [M+Na]$^+$ 537.1268, found 537.1258.

(6a*RS*,12a*RS*)-2,8-Bis(4-nitrophenyl)-6a,12a-diphenyl-6,6a,12,12a-tetrahydrobis[1,3]oxazino[3,2-*a*:3′,2′-*d*]pyrazine-4,10-dione (4g), (6a*RS*,12a*SR*)-2,8-Bis(4-nitrophenyl)-6a,12a-diphenyl-6,6a,12,12a-tetrahydrobis[1,3]oxazino[3,2-*a*:3′,2′-*d*]pyrazine-4,10-dione (5g) (4g*/5g mixture 2/1). White solid; mp 170–171 °C (EtOAc/hexane); yield 80% (on consumed azirine); ^{1}H NMR ($CDCl_3$) δ 3.28* d (2H, J = 15.2 Hz, 4.61 d (1H, J_{AB} = 14.2 Hz), 4.70 d (1H, J_{AB} = 14.5 Hz), 5.66* d (2H, J = 15.2 Hz), 5.86 s (1H), 6.04* s (2H), 7.30–7.35 m (3H), 7.40–7.44 m (6H), 7.47–7.51 m (4H), 7.60 d (2H, J = 7 Hz), 7.83–7.88 m (6H), 8.25* d (4H, J = 9 Hz), 8.30 d (2H, J = 9 Hz); ^{13}C NMR ($CDCl_3$) δ 47.4*, 47.8, 92.2, 93.9*, 100.6*, 101.6, 123.8*, 124.0, 125.1*, 126.0, 126.97, 127.02*, 128.6, 129.2*, 130.0, 130.2*, 137.0, 137.1, 137.7, 149.3*, 149.4, 158.8, 159.6*, 162.1, 162.2, 162.3 (C); IR (KBr, cm^{-1}) ν 1667 (C=O); HRMS-ESI calcd for $C_{34}H_{25}N_4O_8^+$ [M+H]$^+$ 617.1667, found 617.1677.

4,6,8-Tris(4-nitrophenyl)-5,7-dioxa-1-azabicyclo[4.4.1]undeca-3,8-diene-2,10-dione (3i). Light yellow solid; mp 243–244 °C (C_6H_6); yield 56% (on consumed azirine); ^{1}H NMR (DMSO-d_6) δ 5.25 s (2H), 6.63 s (2H), 7.92 d (4H, J = 9 Hz), 8.03 d (2H, J = 9 Hz), 8.19–8.25 m (6H); ^{13}C NMR (DMSO-d_6) δ 46.8, 109.9, 114.0, 124.6, 124.8, 127.9, 128.9, 140.7, 143.7, 149.2, 149.4, 156.1, 164.6; IR (KBr, cm^{-1}) ν 1731 (C=O); HRMS-ESI calcd for $C_{26}H_{16}N_4NaO_{10}^+$ [M+Na]$^+$ 567.0759, found 567.0781.

(6*RS*,7*RS*)-4,6,7-Triphenyl-5-oxa-1-azabicyclo[4.1.0]hept-3-en-2-one (17). A mixture of 2,3-diphenylazirine (**2d**, 96.5 mg, 0.5 mmol) and 5-phenylfuran-2,3-dione (**1a**, 87 mg, 0.5 mmol) in anhydr. benzene (4 mL) was refluxed for 0.5 h. The solvent was removed in vacuum, and the residue was purified by flash chromatography on silica (eluent petroleum ether/ethyl acetate, 5:1) to give compound **17** (45.8 mg, 27%). White solid; mp 164–165.5 °C (EtOAc/hexane); ^{1}H NMR ($CDCl_3$) δ 4.17 s (1H), 5.96 s (1H), 7.19–7.27 m (3H), 7.29–7.32 m (4H), 7.47–7.59 m (6H), 7.75–7.78 m (2H); ^{13}C NMR ($CDCl_3$) δ 48.2, 80.9, 92.3, 126.3, 127.4, 127.5, 127.8, 128.0, 128.2, 128.8, 129.0, 131.2, 132.0, 132.4, 133.0, 165.4, 173.2 (C); IR (KBr, cm^{-1}) ν 1685 (C=O); Crystal data for **17**: $C_{23}H_{17}NO_2$, M = 339.38, monoclinic, space group C 2/c, a = 34.473(4), b = 5.5047(4), c = 17.7523(17) Å, β = 101.619(10)°, U = 3299.7(6) Å^3, F(000) = 1424, Z = 8, D_c = 1.366 mg m^{-3}, μ = 0.087 mm^{-1}. 8210 reflections were collected yielding 3980 unique data (R_{merg} = 0.1241). Final $wR_2(F^2)$ = 0.1376 for all data (303 refined parameters), conventional R_1 (F) = 0.0737 for 1654 reflections with I ≥ 2σ, GOF = 0.902.

3,4,6,8,9-Pentaphenyl-5,7-dioxa-1-azabicyclo[4.4.1]undeca-3,8-diene-2,10-dione (18). A mixture of 3-phenylazirine (**2a**, 117 mg, 1 mmol) and 4,5-diphenylfuran-2,3-dione (**1d**, 250 mg, 1 mmol) in absolute *o*-xylene (5 mL) was refluxed for 2 h. The reaction was monitored by IR-spectroscopy. The solvent was partially removed in vacuum, crystalline substance thus obtained

was filtered and washed with hexane/diethyl ether. The solvent was removed fully in vacuum, and the residue was purified by recrystallization from hexane/AcOEt mixture (5:1) to give compound **9** (53 mg, 34% on consumed **1d**) as white solid; mp 221–223 °C (hexane/CH_2Cl_2), (Lit.[1] mp 221–223 °C).

Methyl 3-(4-methoxyphenyl)-3-(7-(4-methoxyphenyl)-5-oxo-2-phenyl-2,3,4,5-tetrahydro-1,4-oxazepin-2-yloxy)acrylate (19). The solution of 20.9 mg (0.045 mmol) 4,8-bis-(4-methoxyphenyl)-6-phenyl-5,7-dioxa-1-azabicyclo[4.4.1]undeca-3,8-diene-2,10-dione (**3d**) in MeOH/CH_2Cl_2 mixture (1:2) was heated to 40 °C with stirring for 20 h. The reaction was monitored by TLC. The solvent was removed in vacuum, and the residue was recrystallized from hexane/Et_2O mixture to give compound **19** (20 mg, 90%) as white solid. Mp 58–59 °C (hexane/Et_2O); ^{1}H NMR ($CDCl_3$) δ 3.72 s (3H), 3.76 s (3H), 3.88 s (3H), 3.96 br (1H), 4.13 dd (1H, J_{AB} = 15.2 Hz, J_{AX} = 6.5 Hz), 5.36 s (1H), 5.91 br (1H), 6.40 br (1H), 6.54 d (2H, J = 9 Hz), 6.90 d (4H, J = 9 Hz), 7.25–7.33 m (3H), 7.42 d (2H, J = 9 Hz), 7.47 d (2H, J = 9 Hz); ^{13}C NMR ($CDCl_3$) δ 51.0, 55.2, 55.4, 65.8, 98.8, 106.5, 113.1, 113.6, 126.8, 127.7, 128.1, 128.3, 128.7, 129.3, 129.4, 130.9, 136.5, 155.4, 160.8, 161.1, 164.9, 165.3, 169.0; IR (KBr, cm^{-1}) ν 1717 (C=O); HRMS-ESI calcd for $C_{29}H_{27}NKO_7^+$ [M+K]$^+$ 540.1419, found 540.1431.

2-Methoxy-2,3,7-triphenyl-3,4-dihydro-1,4-oxazepin-5(2*H*)-one (20). The solution of 20.3 mg (0.06 mmol) 4,6,7-triphenyl-5-oxa-1-aza[4.1.0]hept-3-en-2-one (**17**) in MeOH/CH_2Cl_2 mixture (1/2) was heated to 40 °C with stirring for 5 h. The reaction was monitored by TLC. The solvent was removed in vacuum, and the residue was purified by flash chromatography on silica (eluent petroleum ether/ethyl acetate, 1:7) to give compound **20** (19.4 mg, 87%). White solid; mp 227–231 °C (CH_2Cl_2/MeOH); ^{1}H NMR ($CDCl_3$) δ 3.17 s (3H), 4.80 d (1H, J = 7.3 Hz), 6.22 d (1H, J = 1.8 Hz), 6.60 br d (1H, J = 5.8 Hz), 6.77 d (2H, J = 7.6 Hz), 7.06–7.10 m (2H), 7.14–7.19 m (1H), 7.38–7.45 m (8H), 7.73–7.76 m (2H); ^{13}C NMR ($CDCl_3$) δ 51.3, 66.0, 102.5, 105.7, 126.5, 127.7, 127.8, 128.1, 128.3, 128.6, 129.1, 130.2, 135.0, 136.4, 137.2, 154.8, 168.9; IR (KBr, cm^{-1}) ν 1704 (C=O); HRMS-ESI calcd for $C_{24}H_{22}NO_3^+$ [M+H]$^+$ 372.1594, found 372.1597.

[1]Khlebnikov, A. F.; Novikov, M. S.; Pakalnis, V. V.; Yufit, D. S. *J. Org. Chem.* **2011**, *76*, 9344

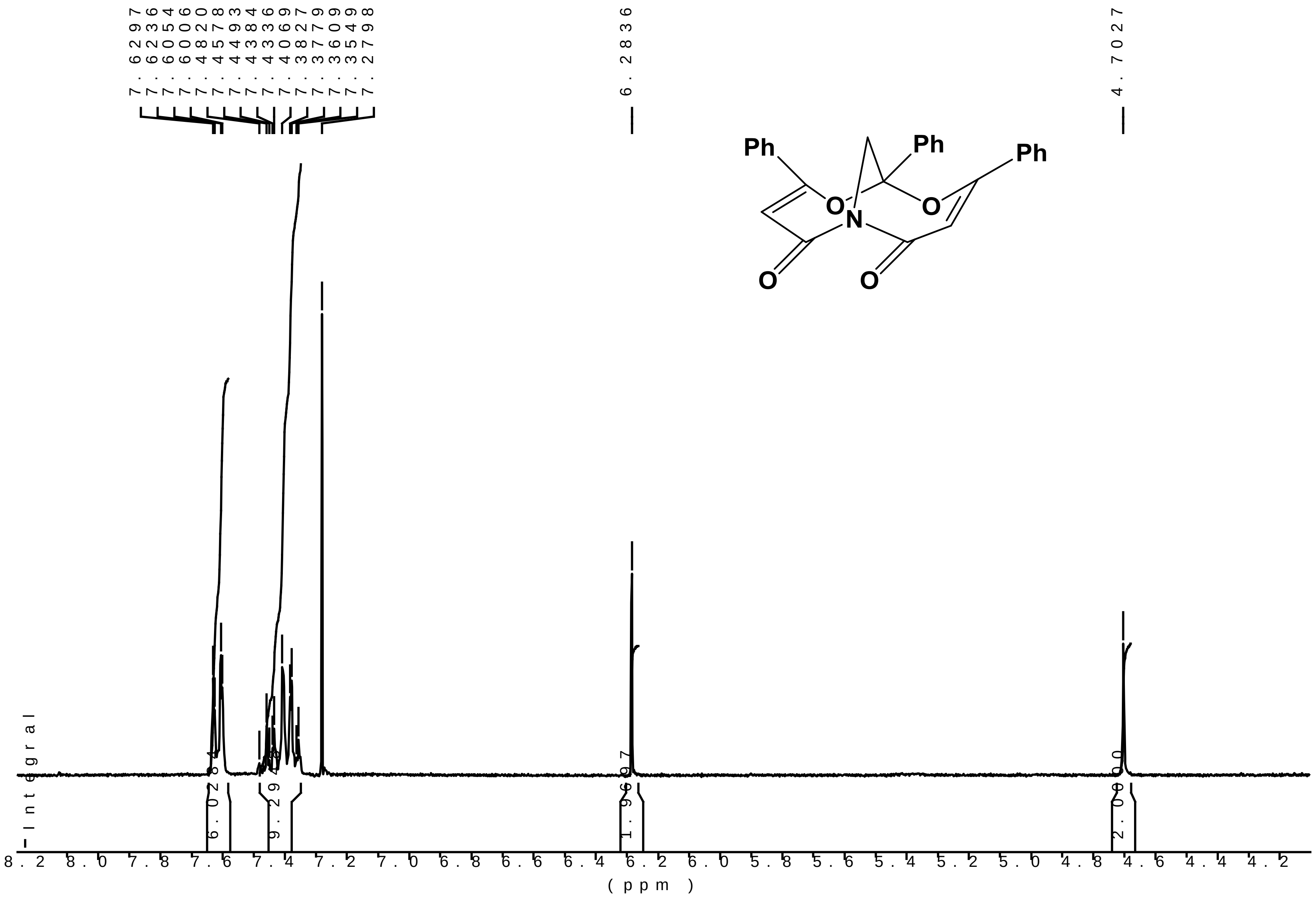

^{1}H NMR spectrum of compound **3a**.

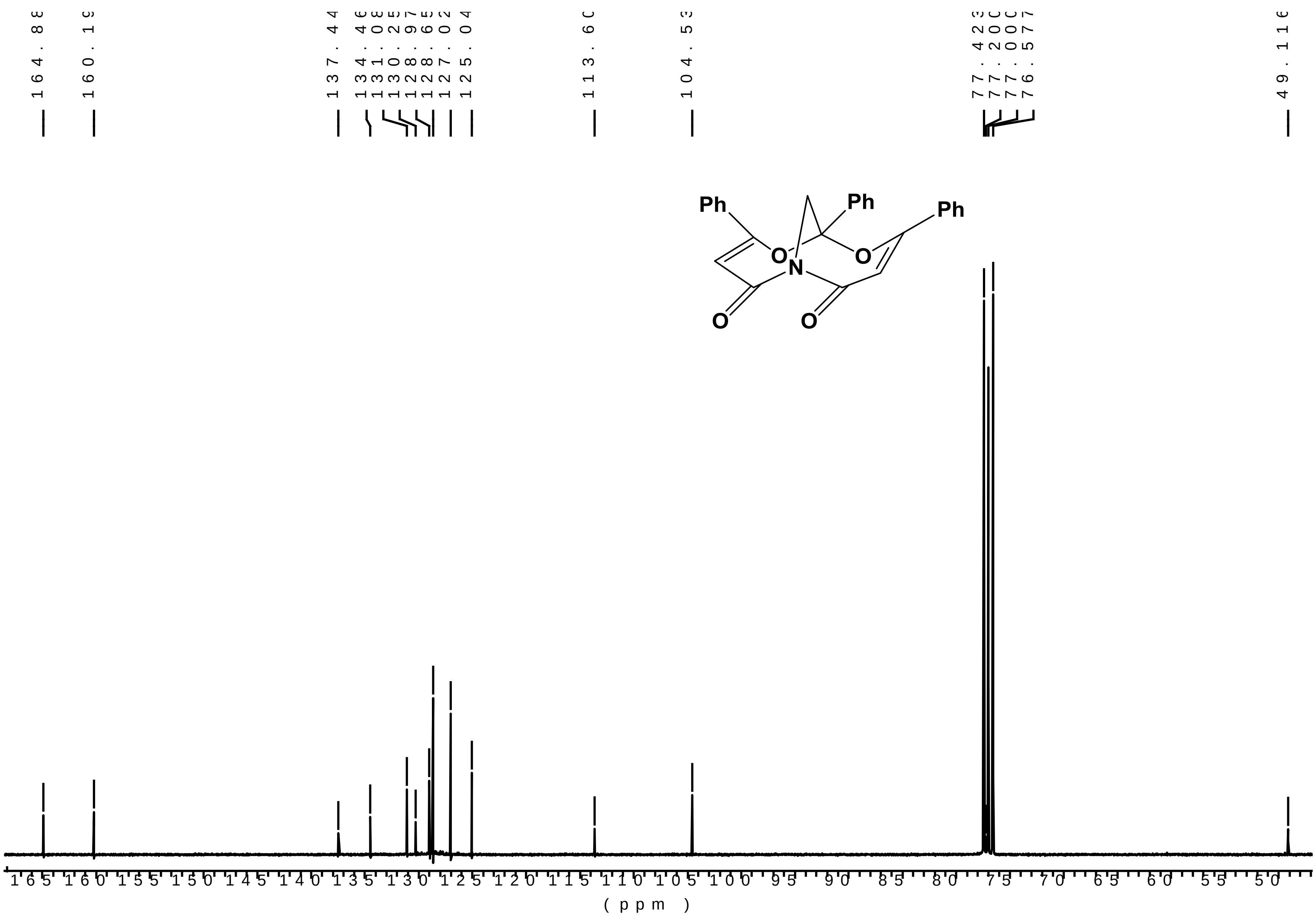

^{13}C NMR spectrum of compound **3a**.

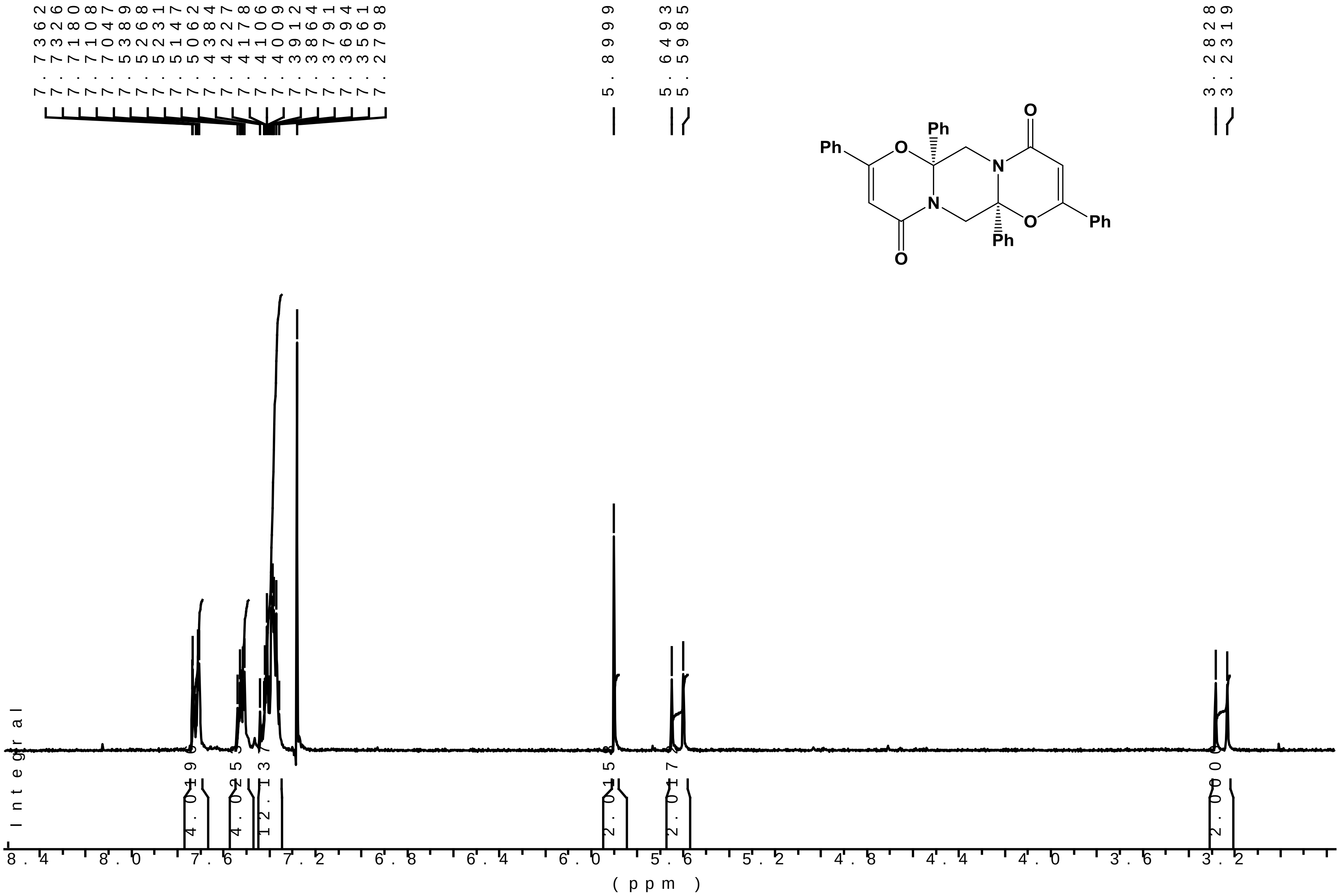

^{1}H NMR spectrum of compound **4a**.

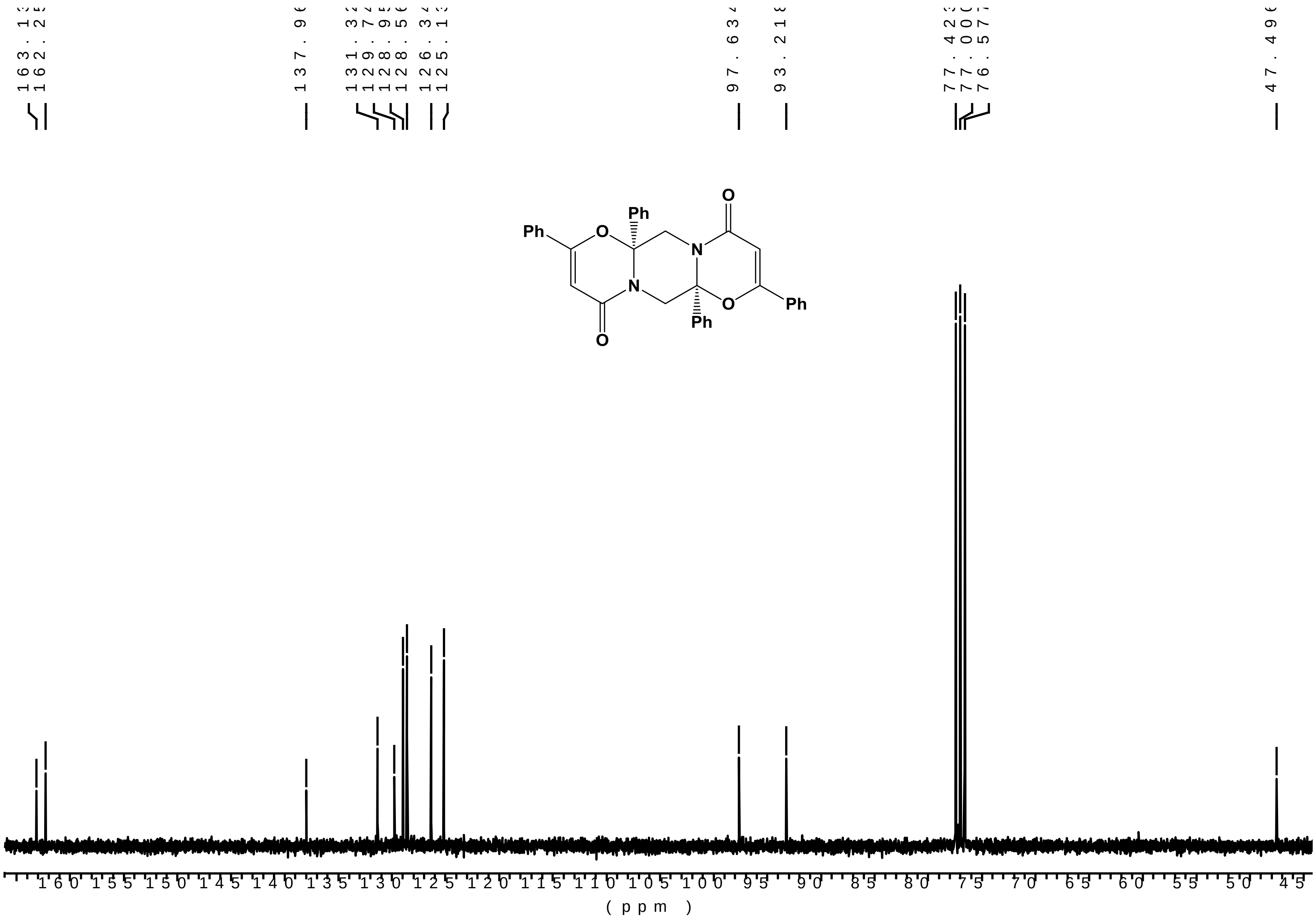

^{13}C NMR spectrum of compound **4a**.

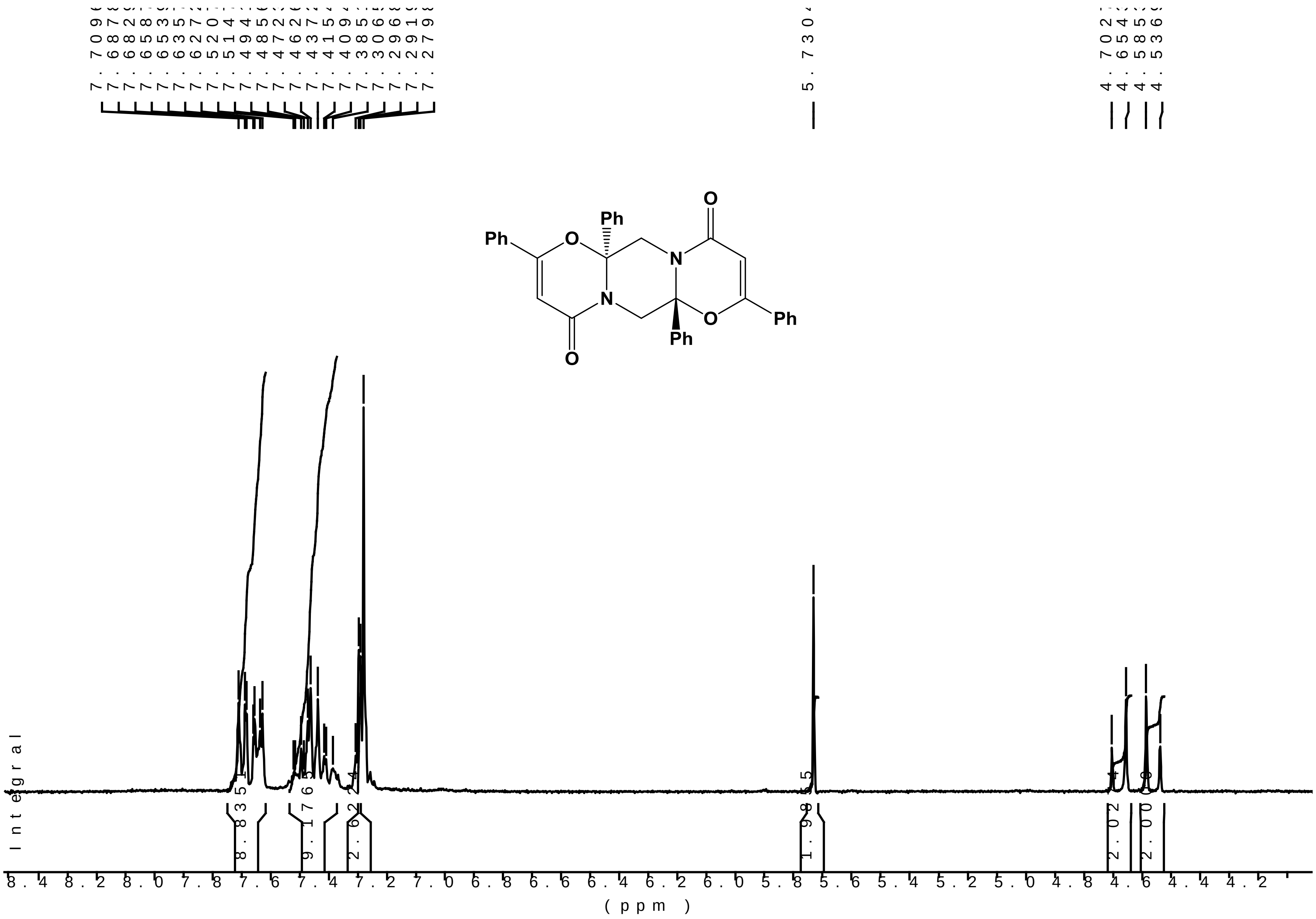

^{1}H NMR spectrum of compound **5a**.

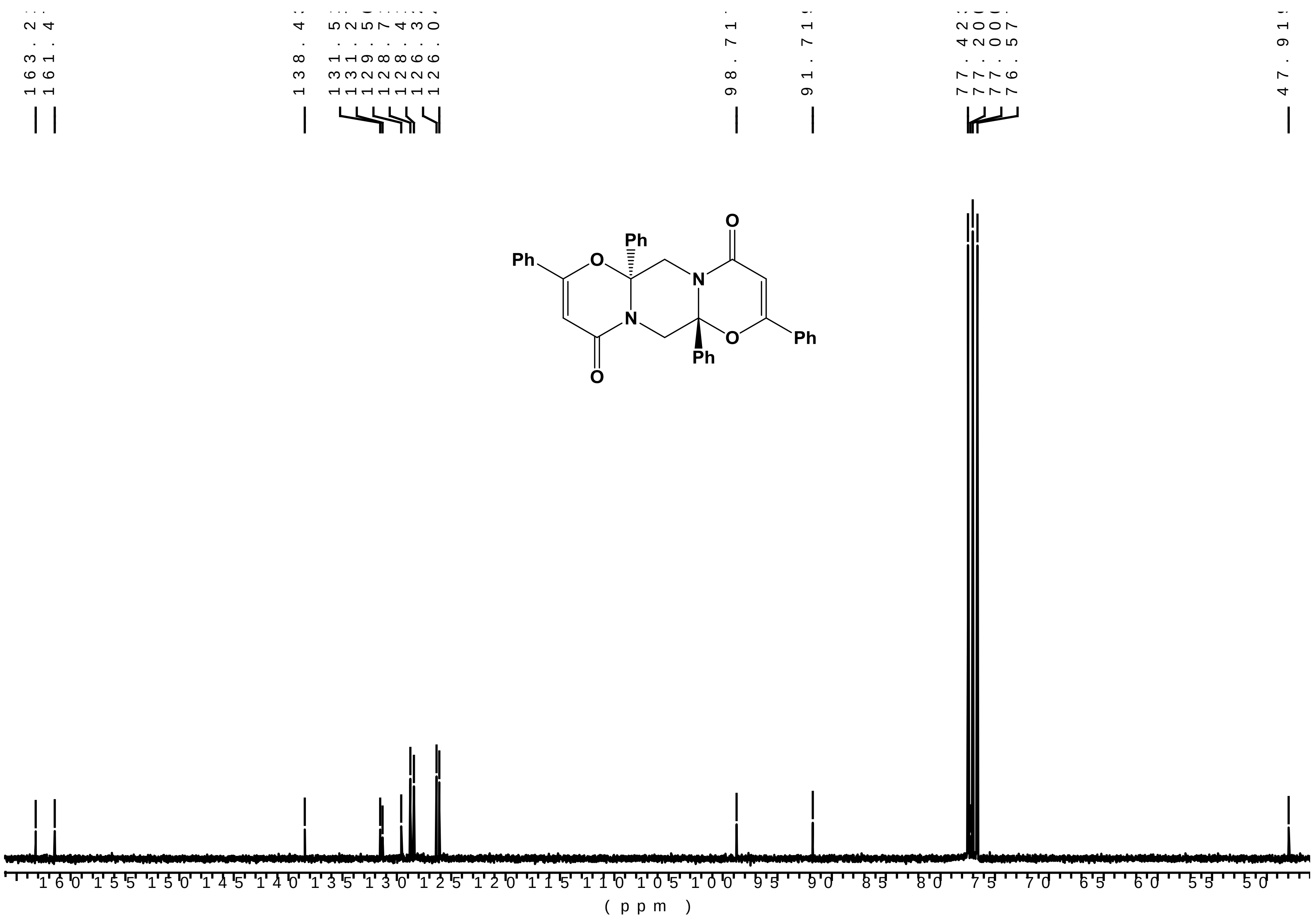

^{13}C NMR spectrum of compound **5a**.

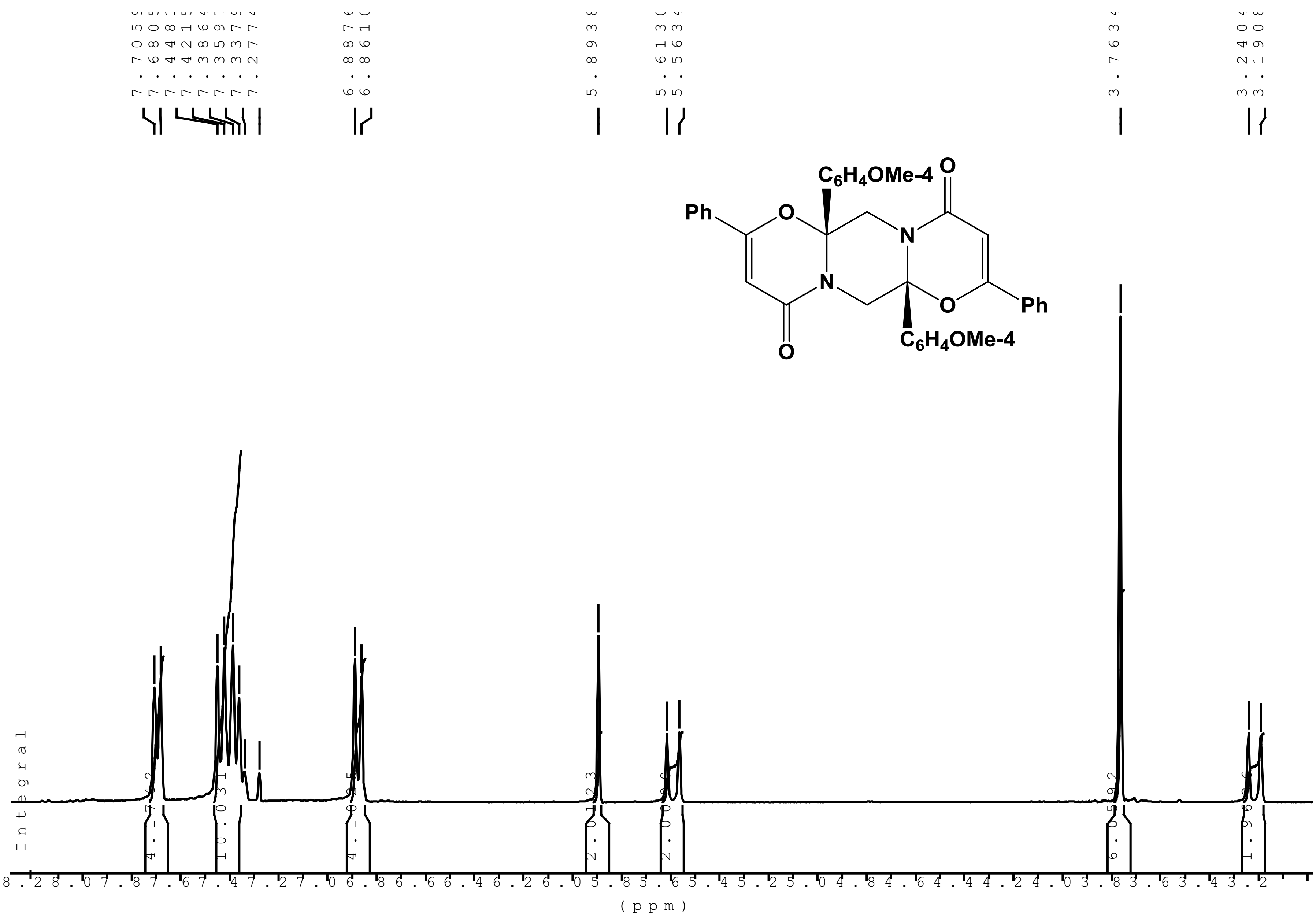

^{1}H NMR spectrum of compound **4b**.

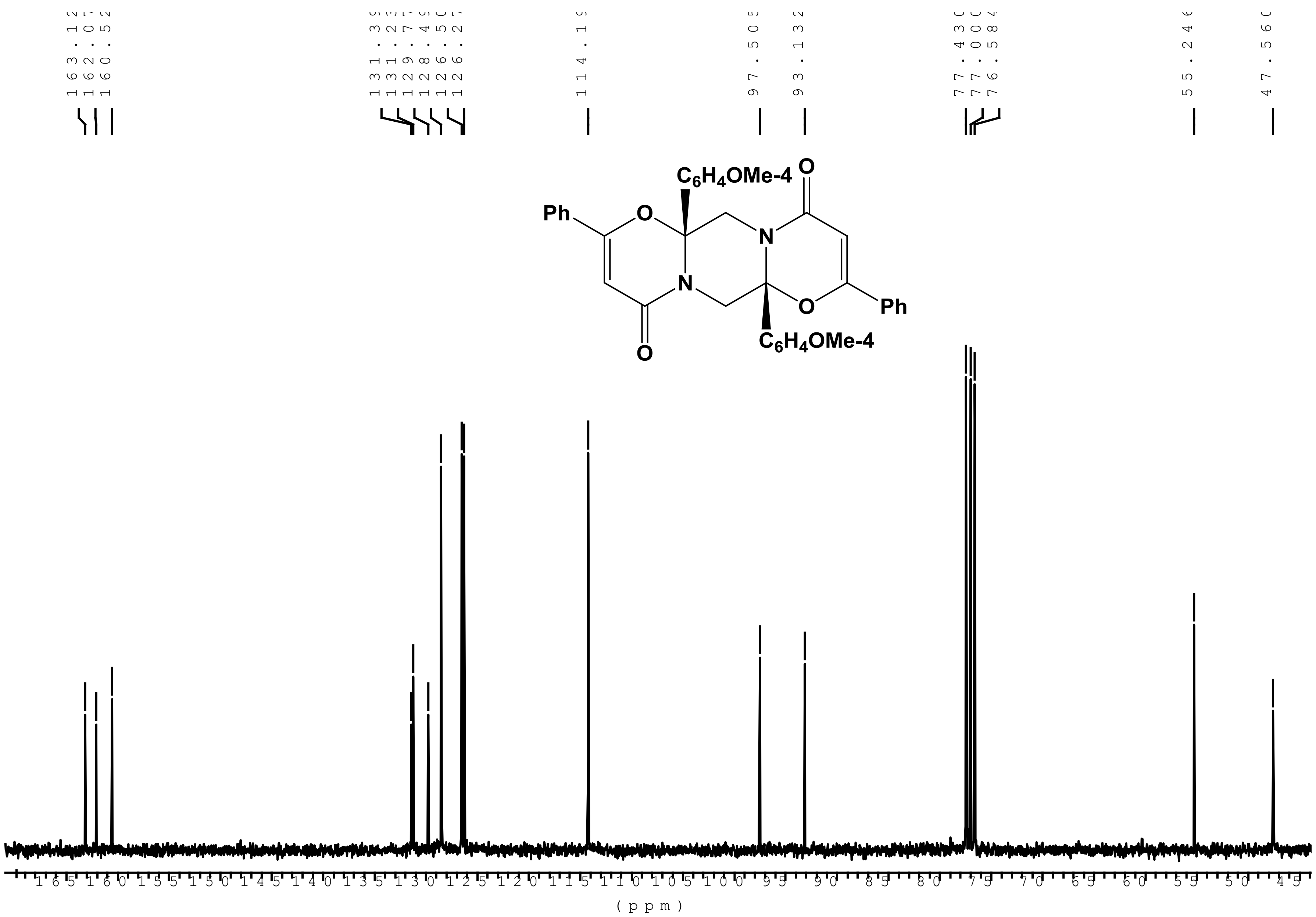

^{13}C NMR spectrum of compound **4b**.

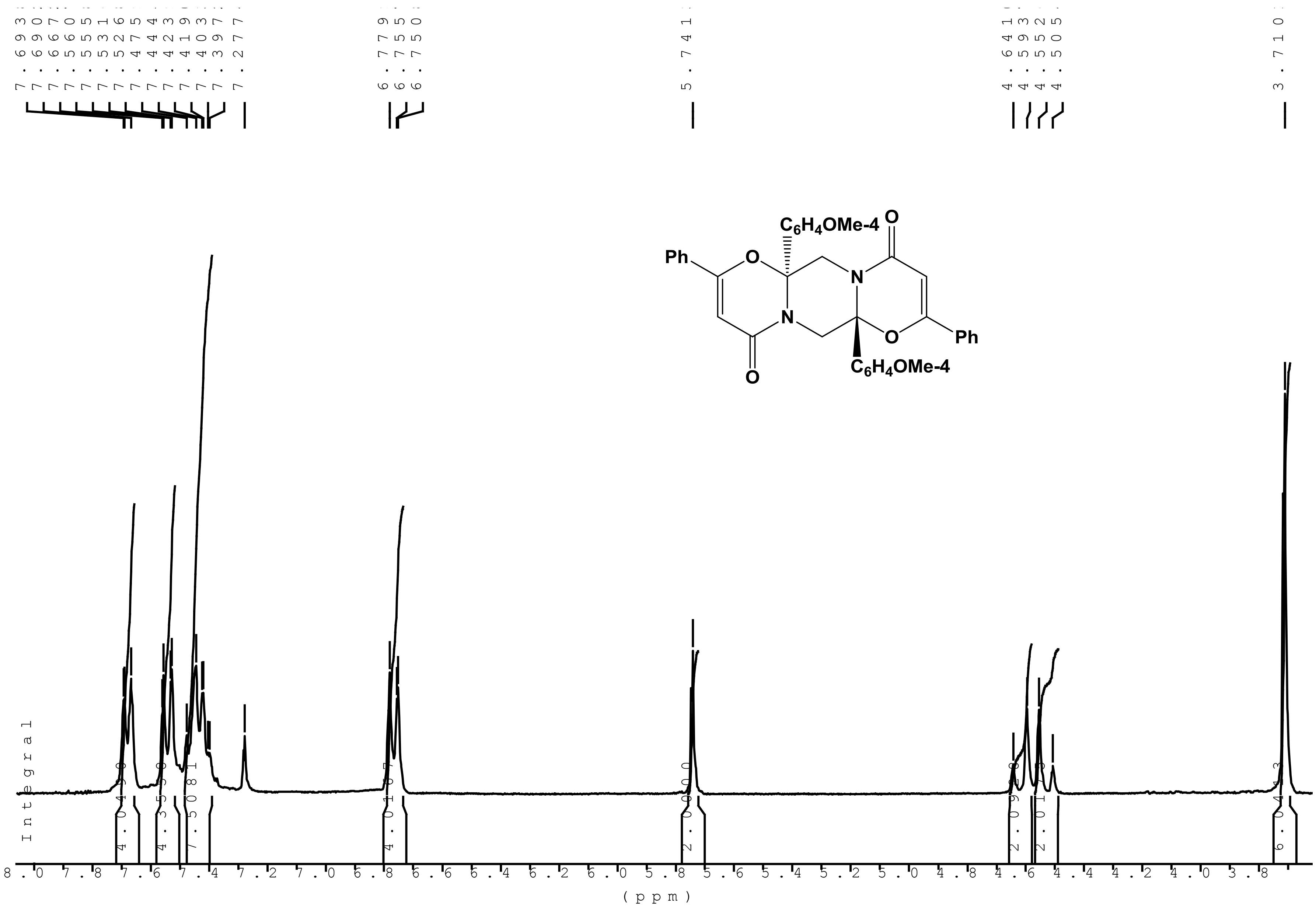

^{1}H NMR spectrum of compound **5b**.

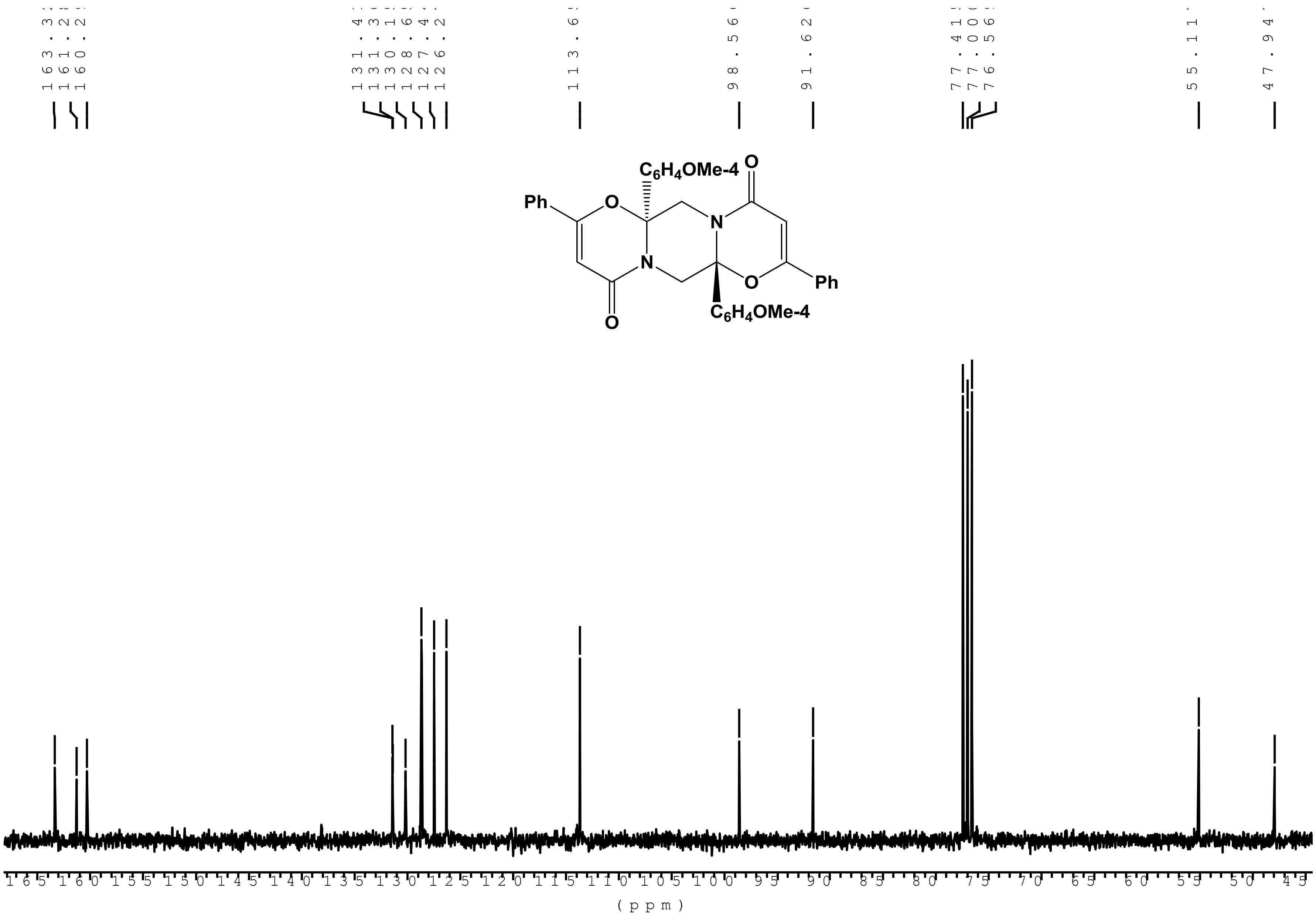

^{13}C NMR spectrum of compound **5b**.

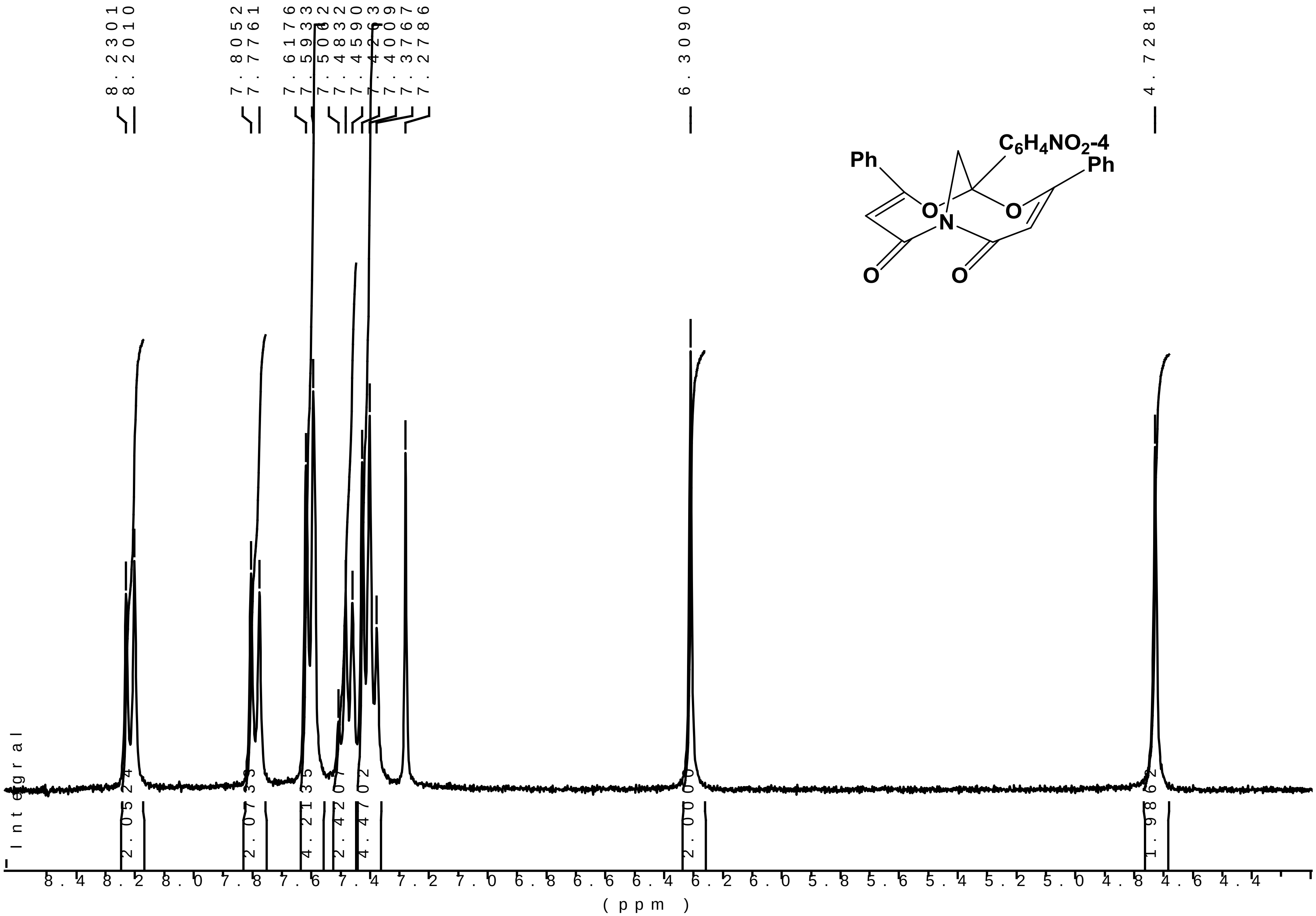

^{1}H NMR spectrum of compound **3c**.

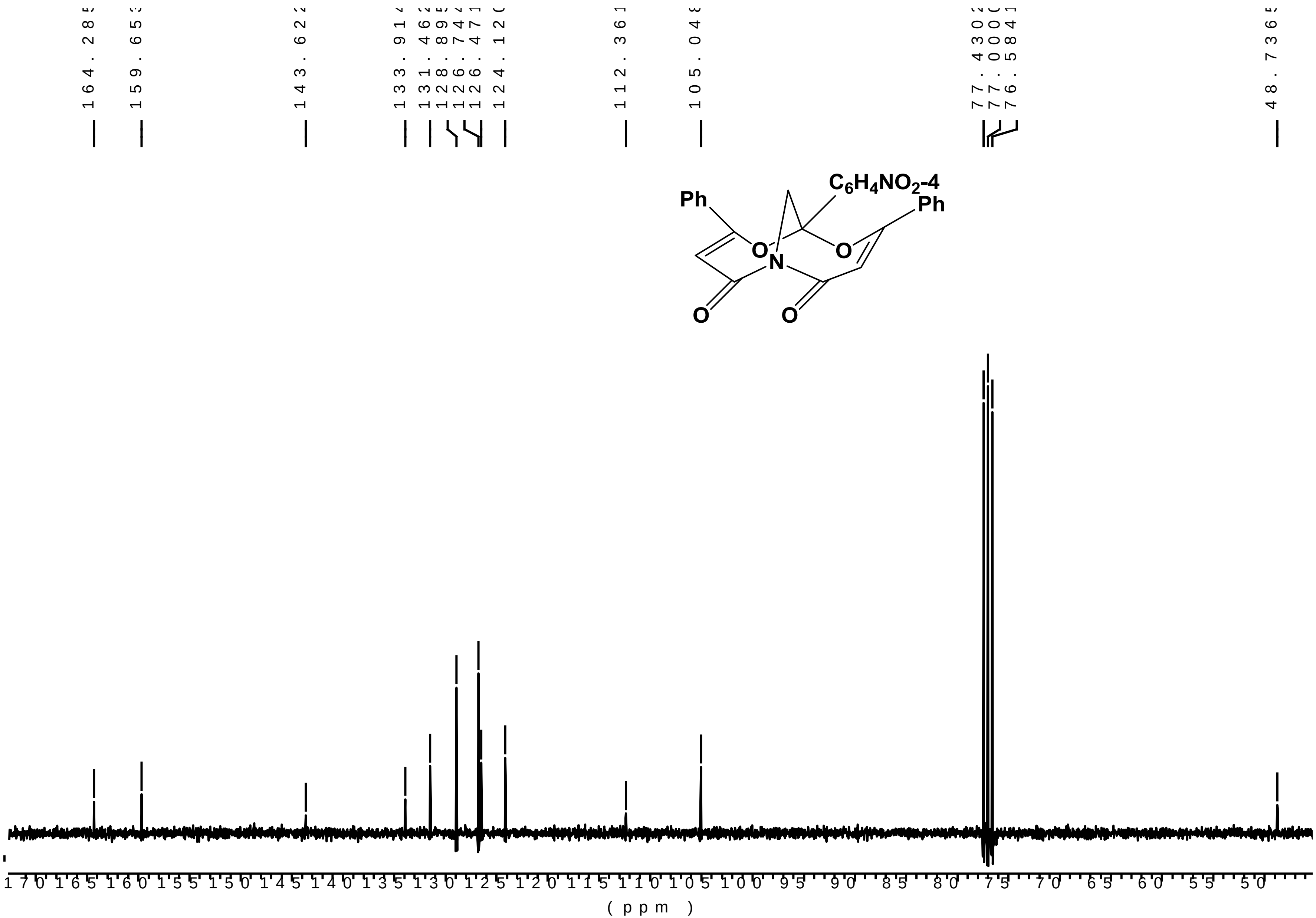

^{13}C NMR spectrum of compound **3c**.

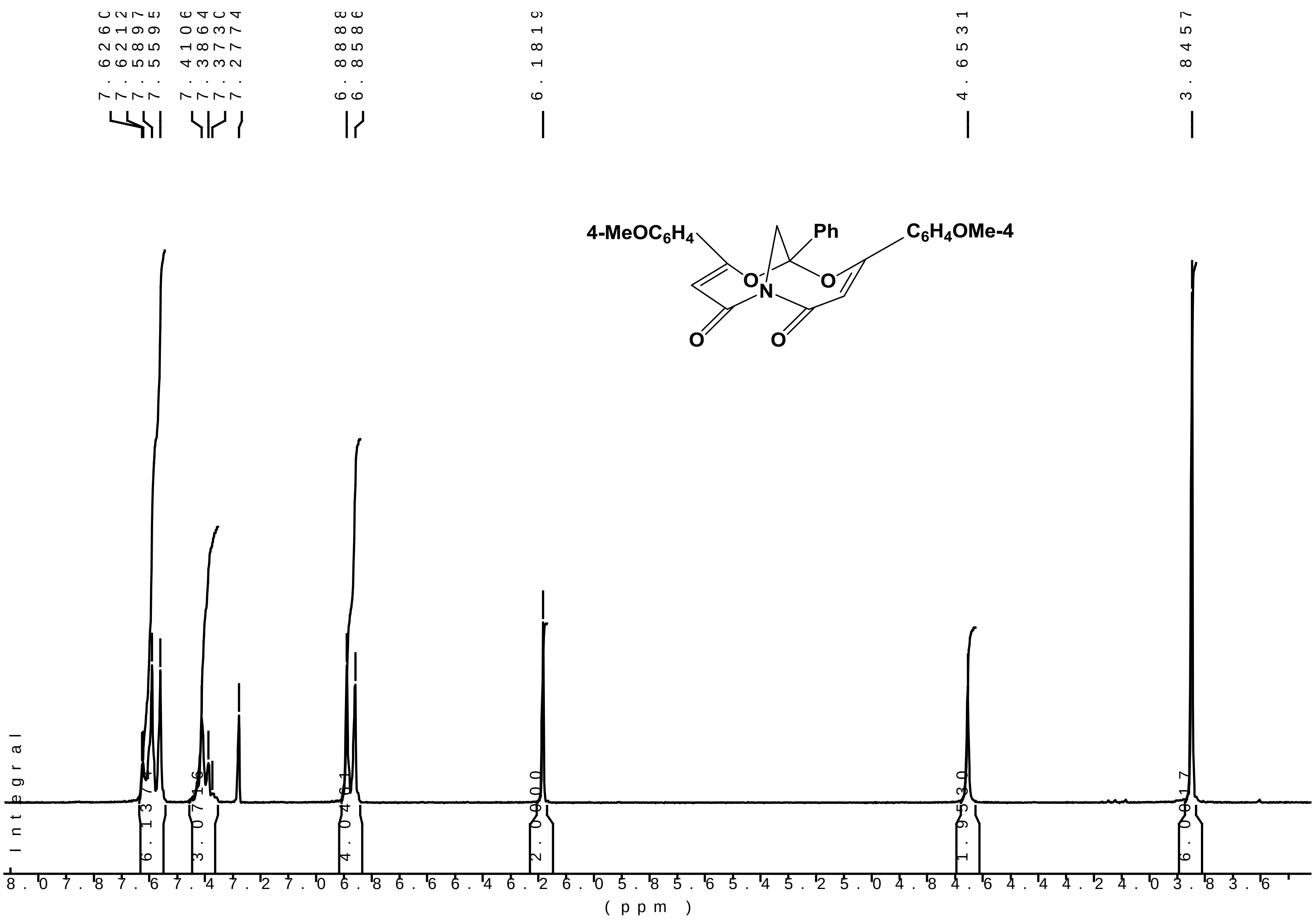

^{1}H NMR spectrum of compound **3d**.

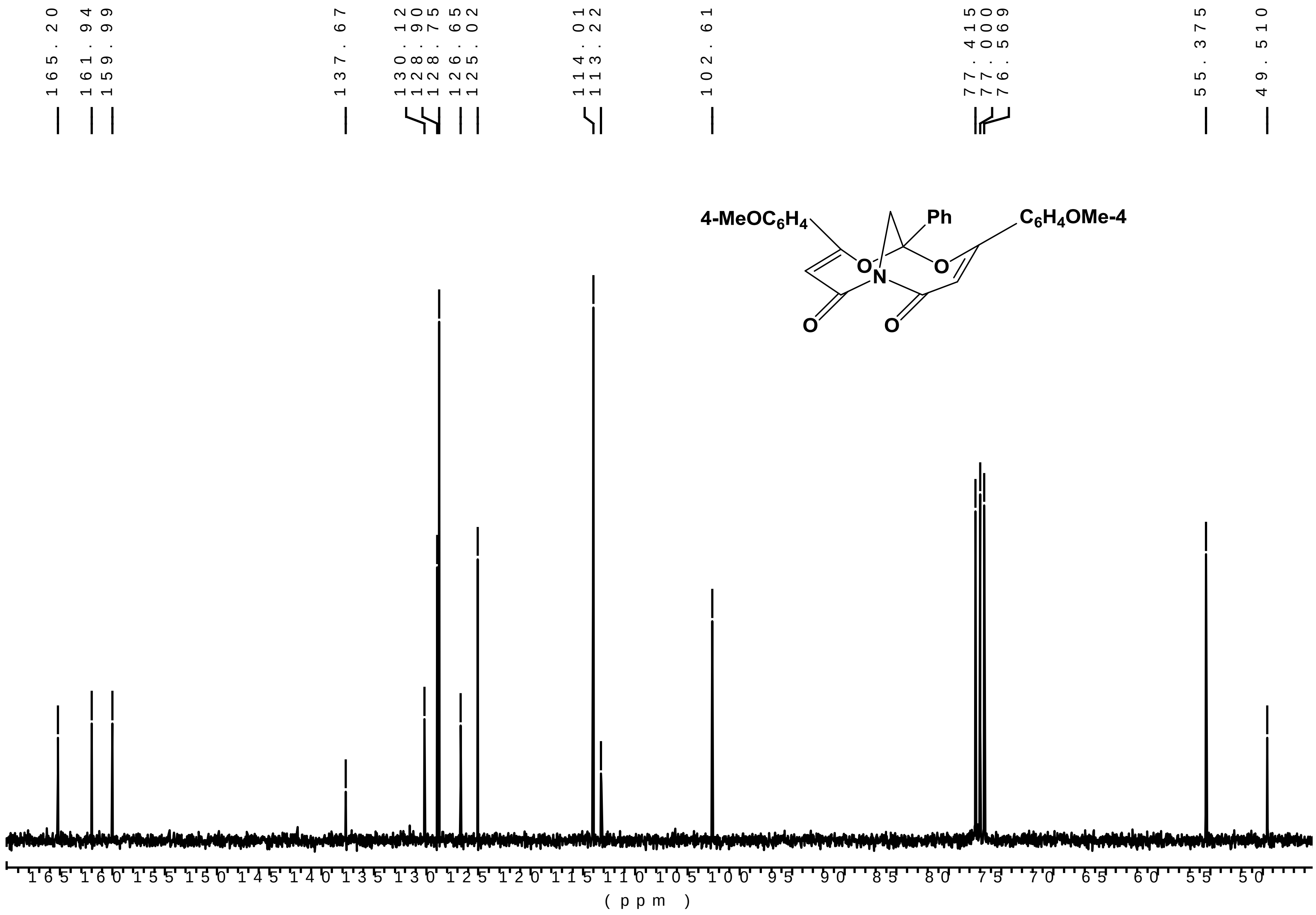

^{13}C NMR spectrum of compound **3d**.

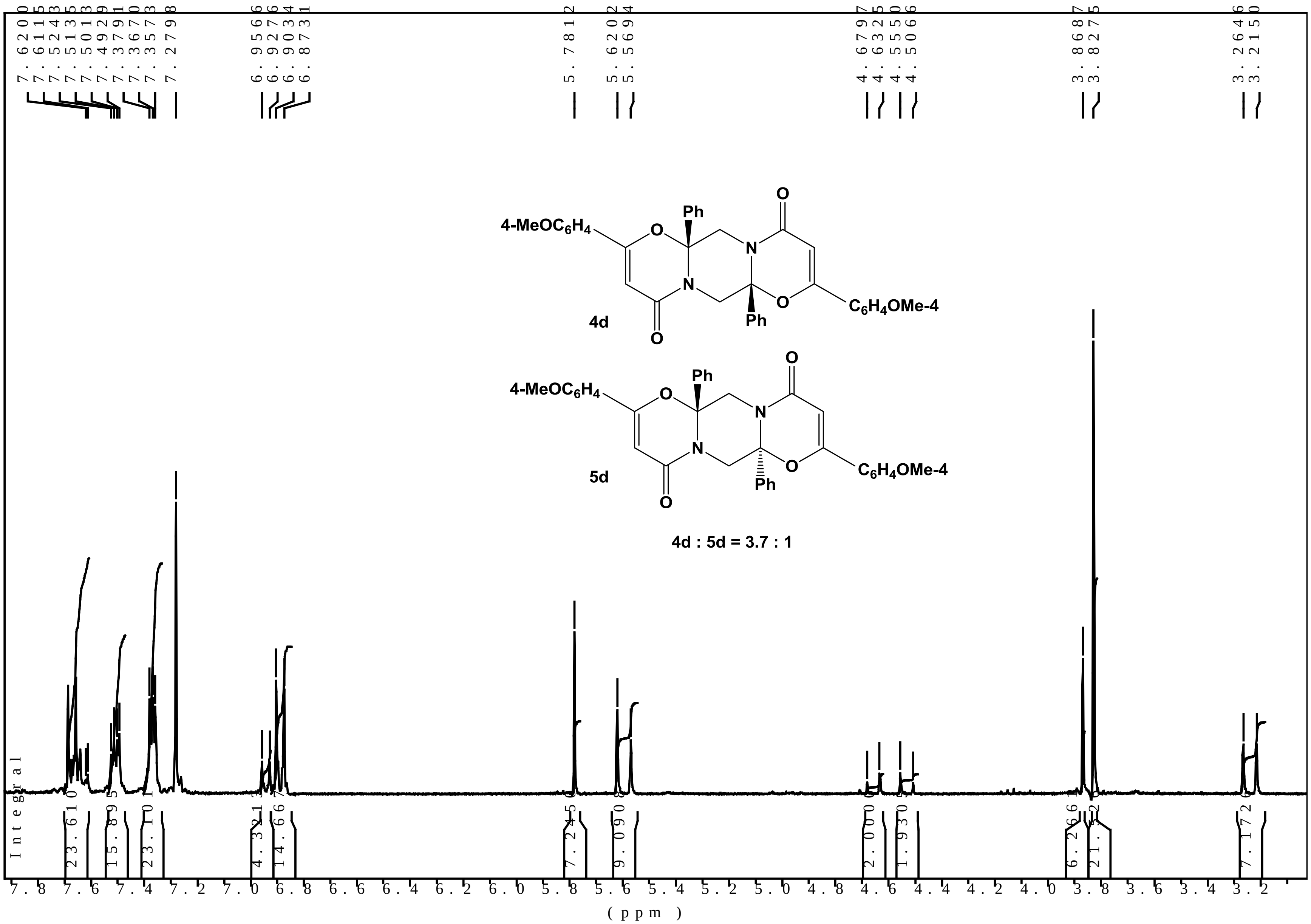

^{1}H NMR spectrum of compounds **4d, 5d** (3.7 : 1).

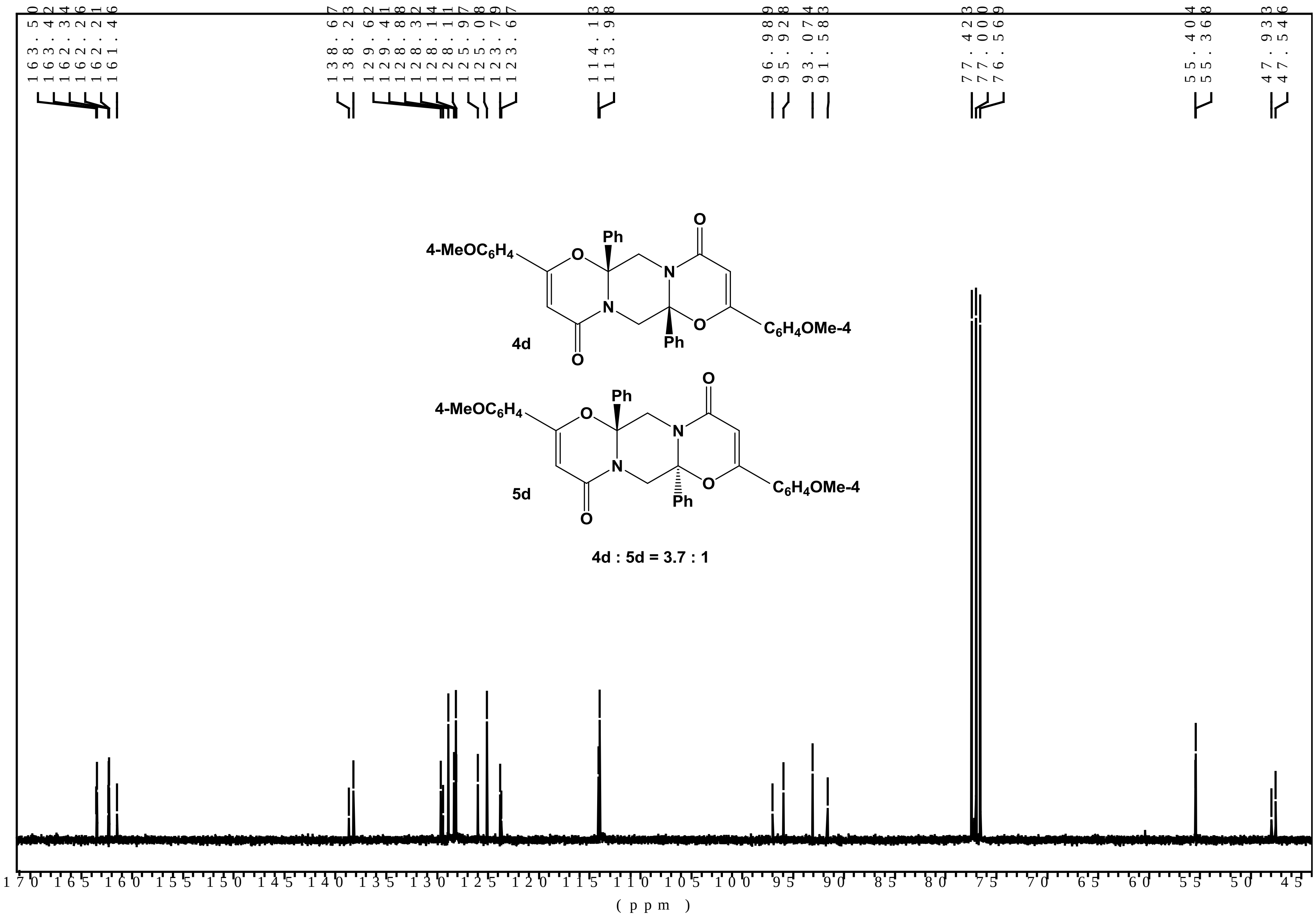

^{13}C NMR spectrum of compounds **4d, 5d** (3.7 : 1).

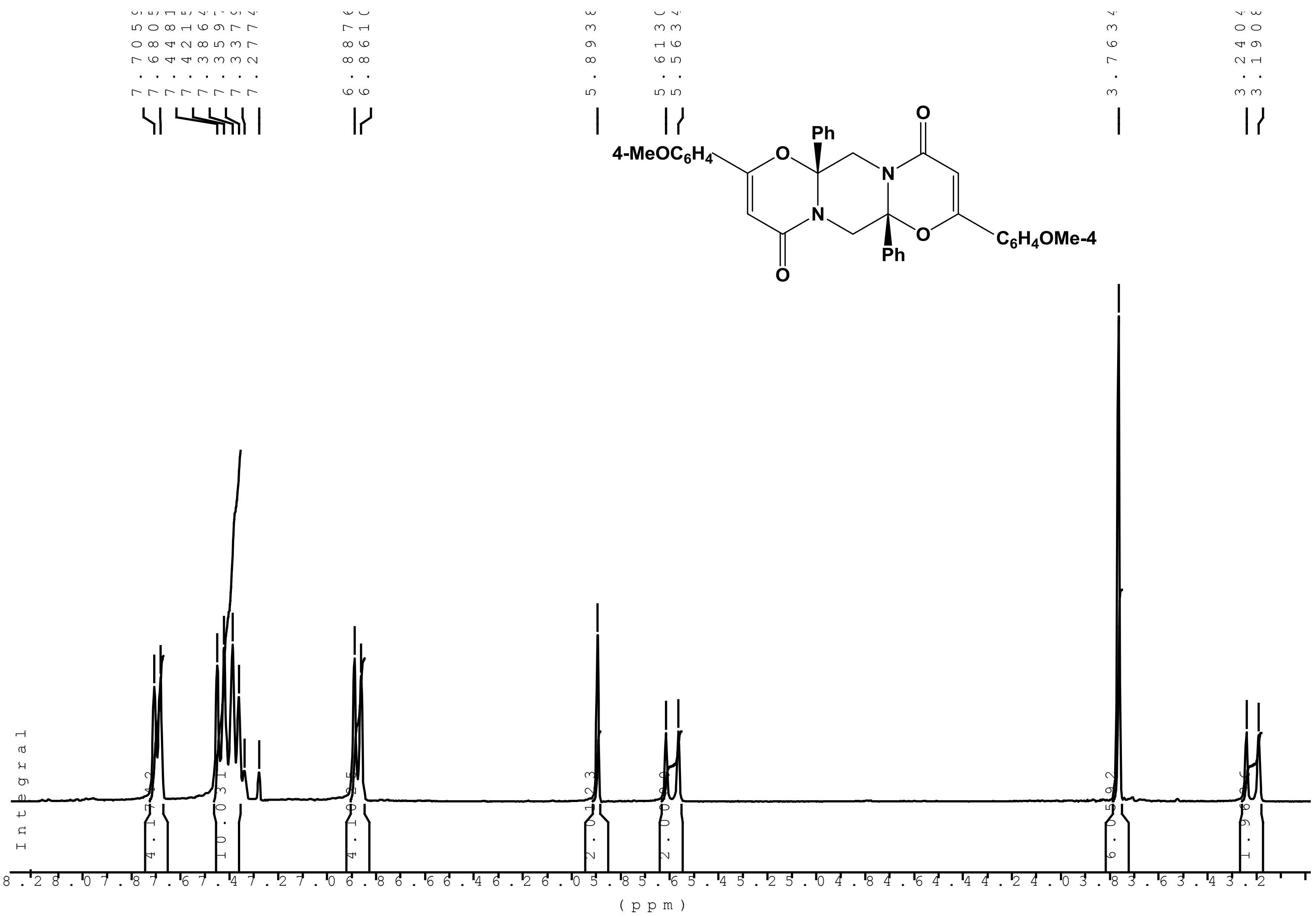

^{1}H NMR spectrum of compound **4d**.

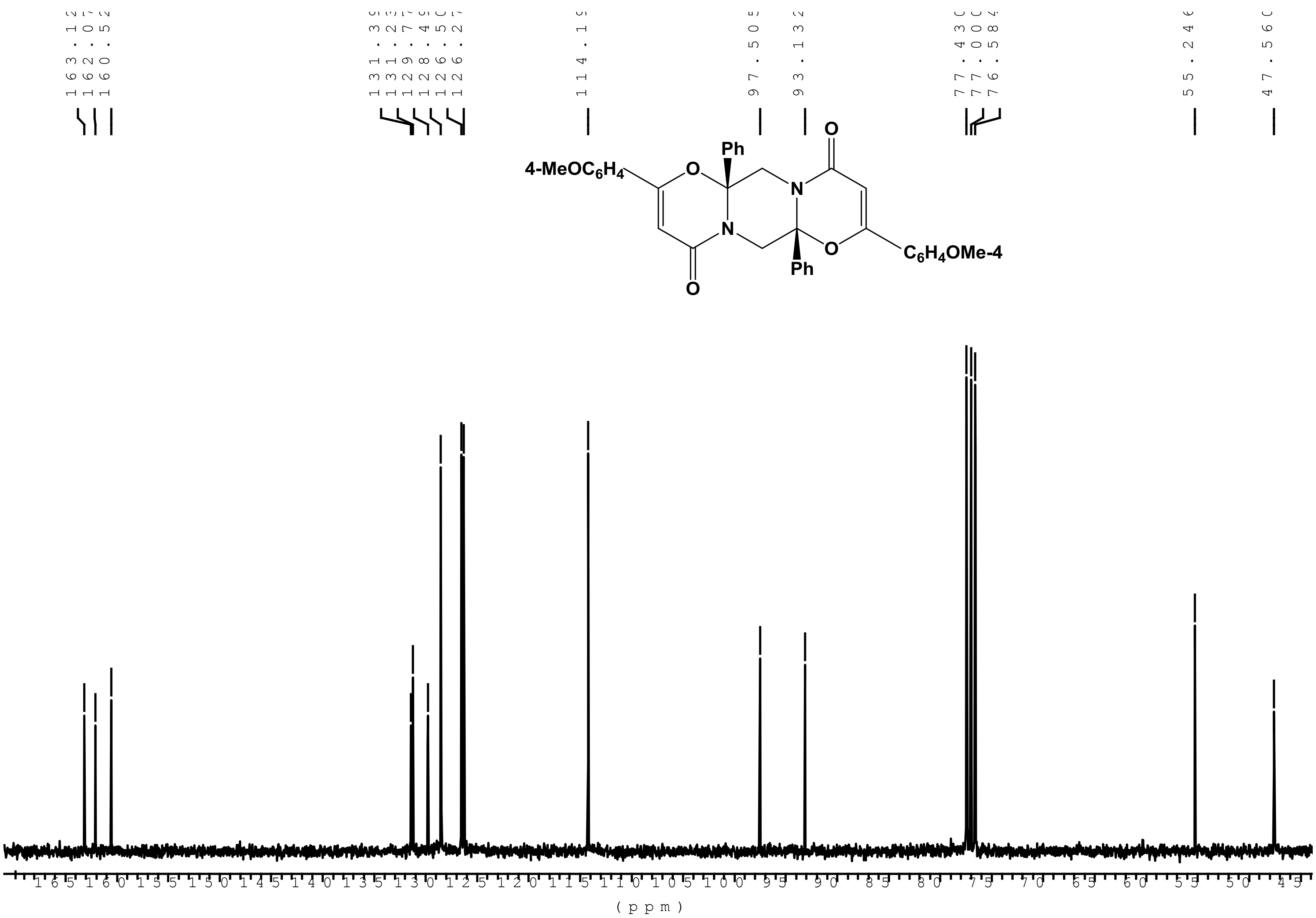

^{13}C NMR spectrum of compound **4d**.

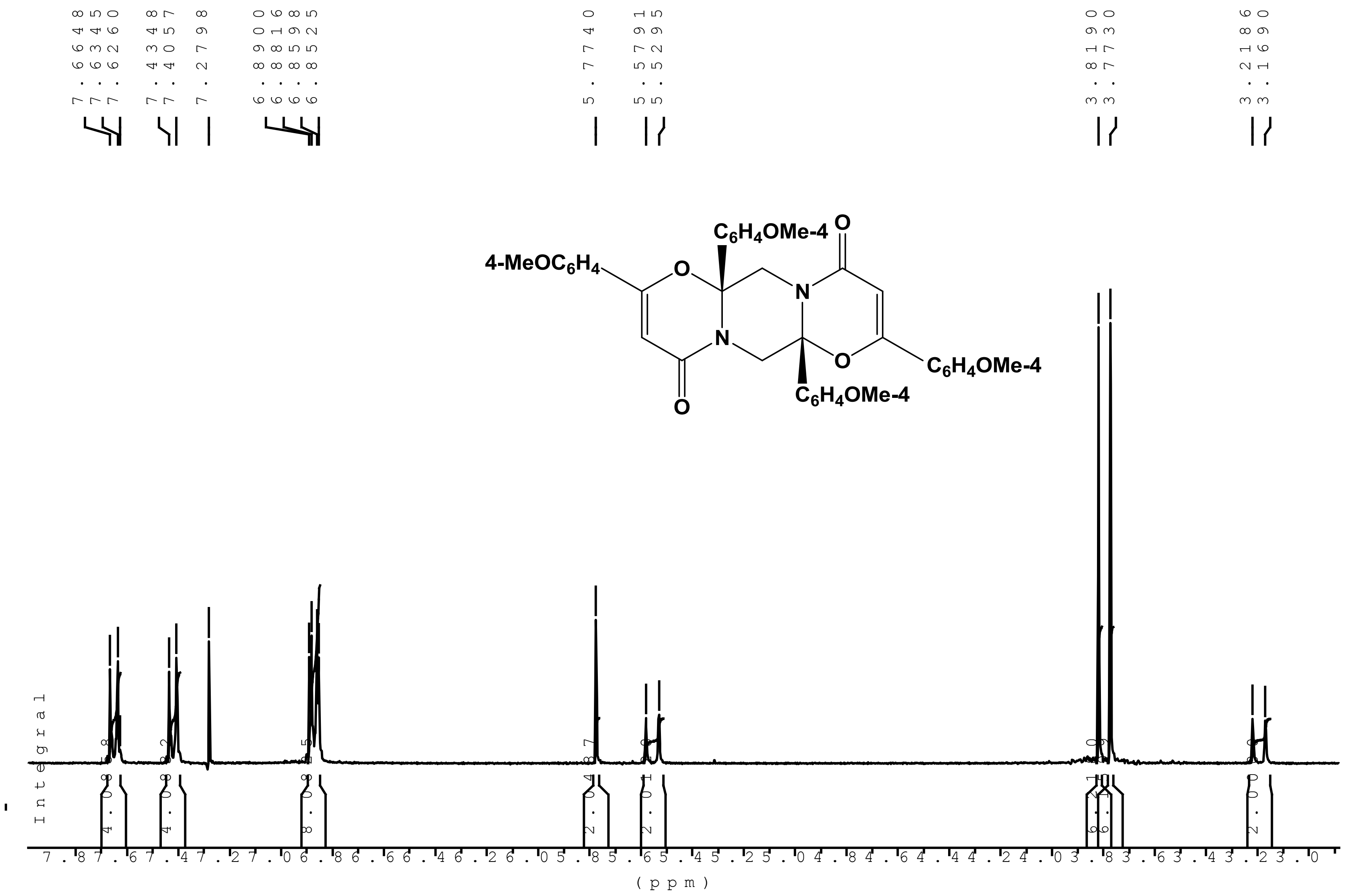

^{1}H NMR spectrum of compound **4e**.

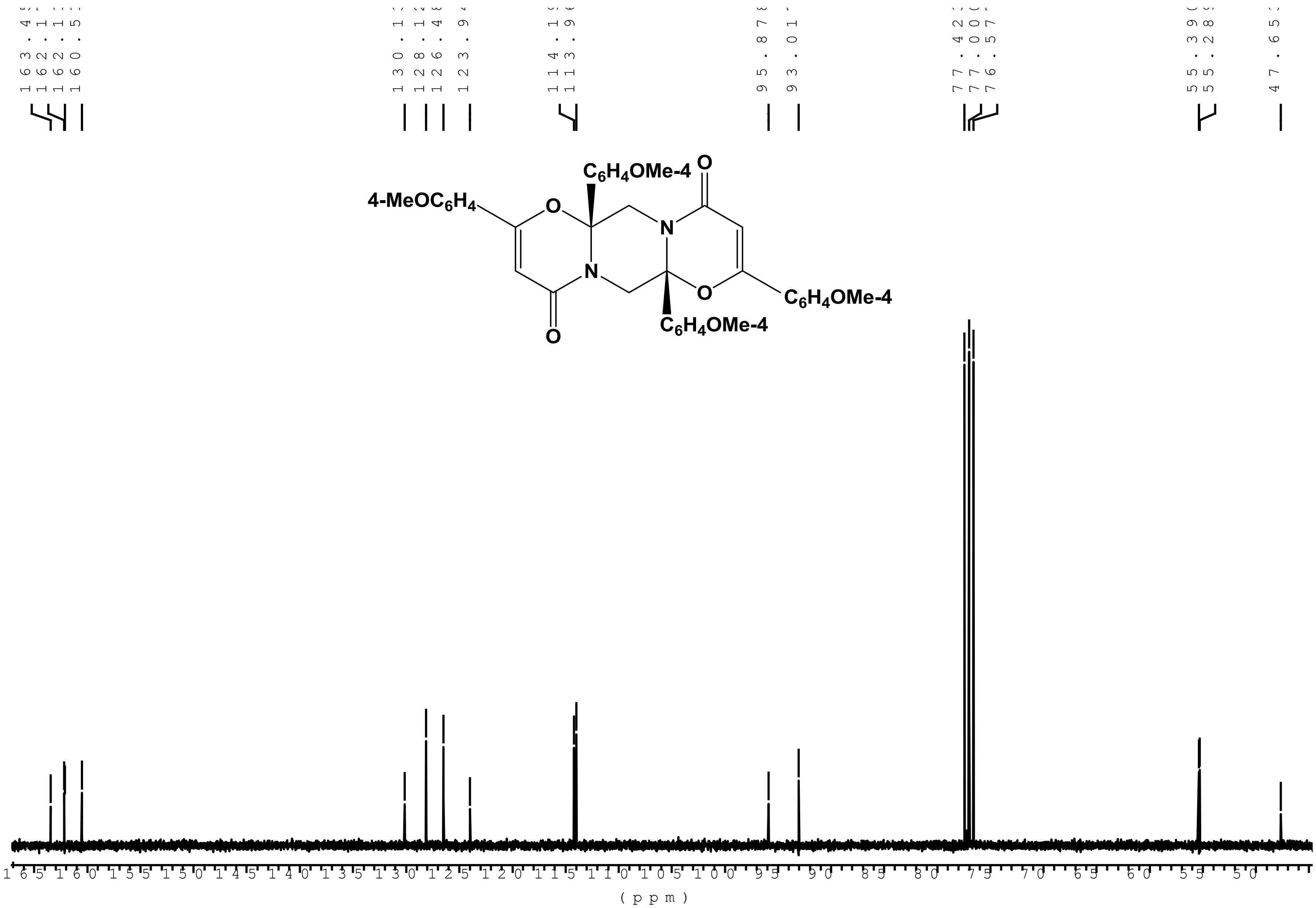

^{13}C NMR spectrum of compound **4e**.

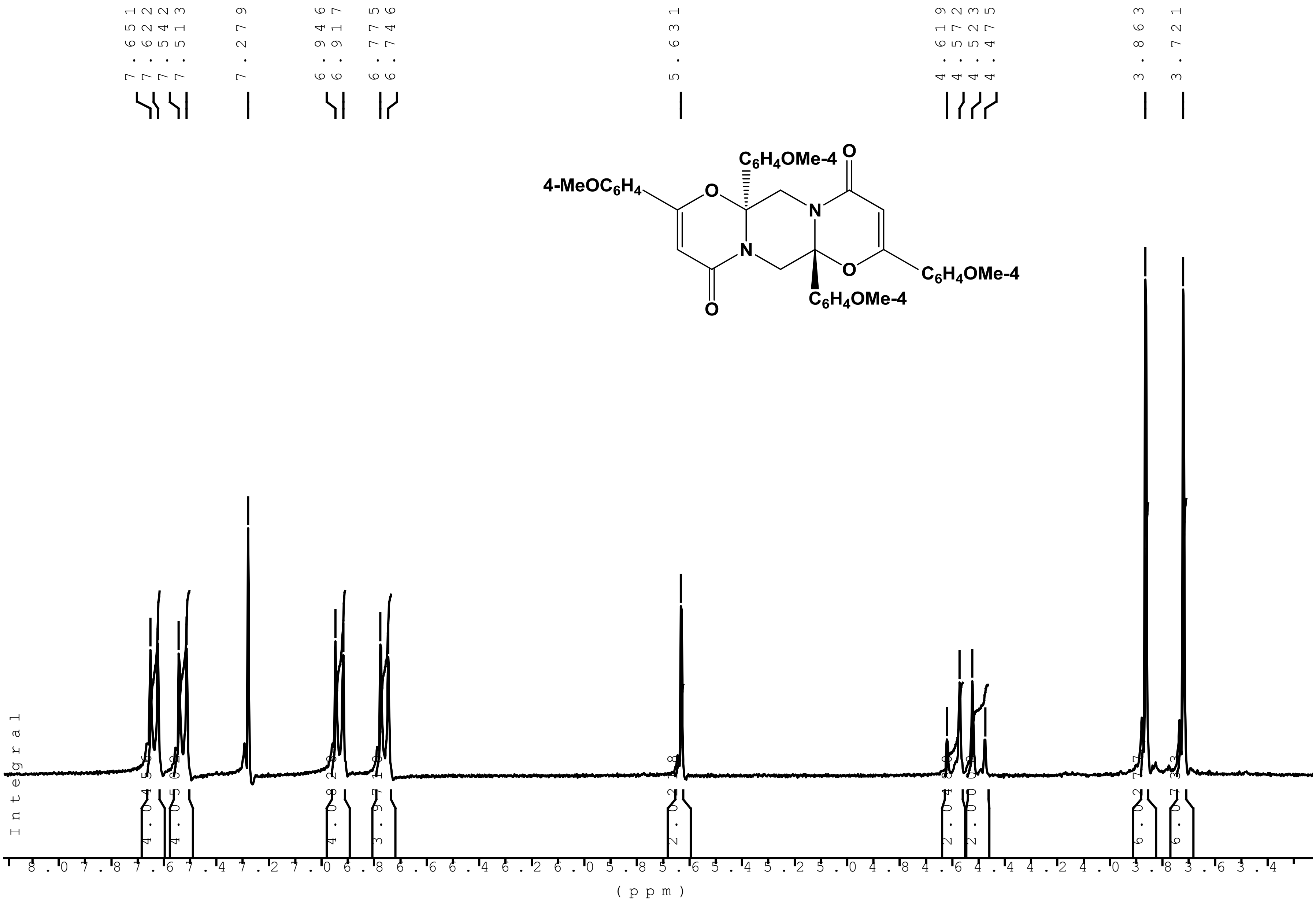

^{1}H NMR spectrum of compound **5e**.

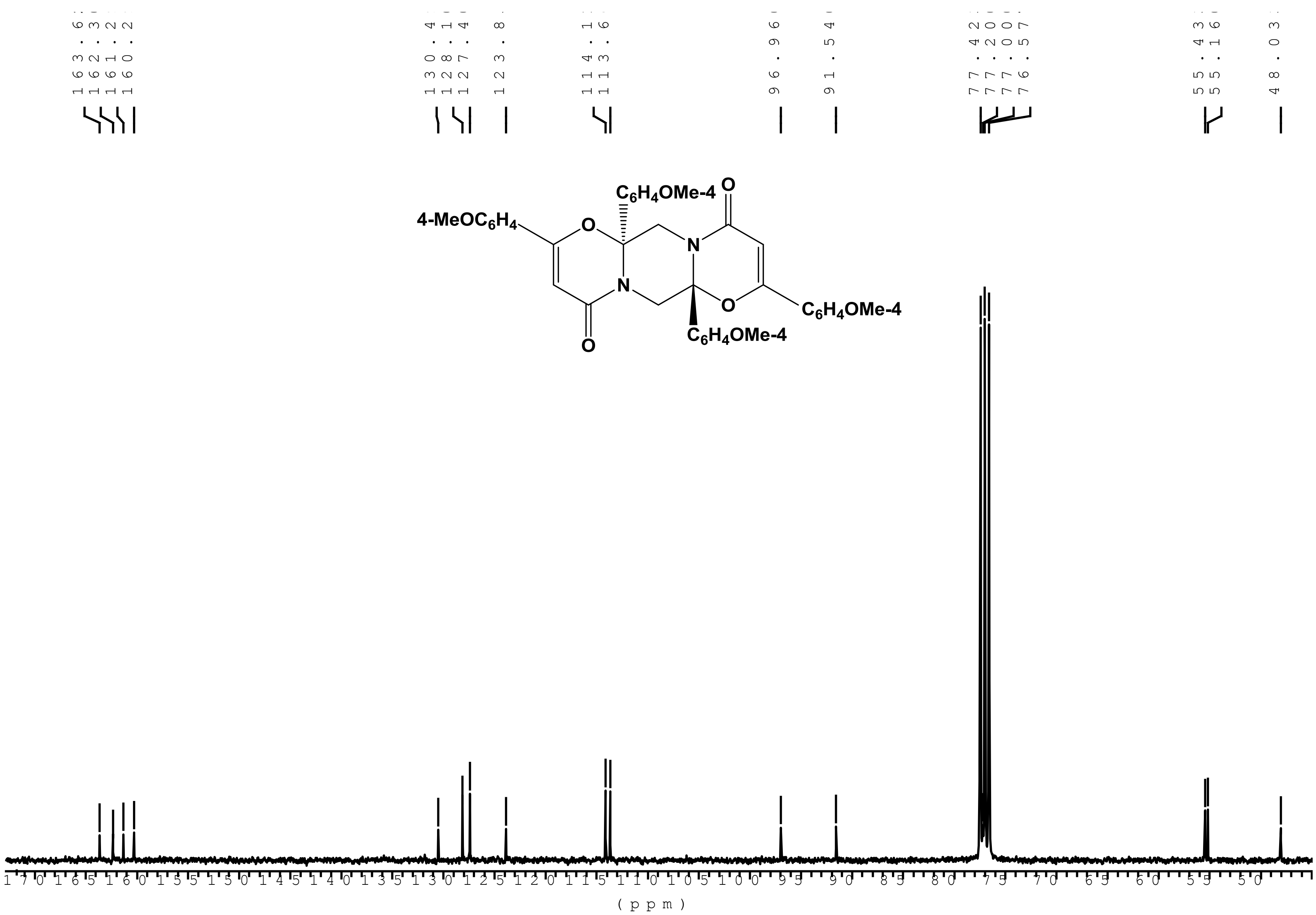

^{13}C NMR spectrum of compound **5e**.

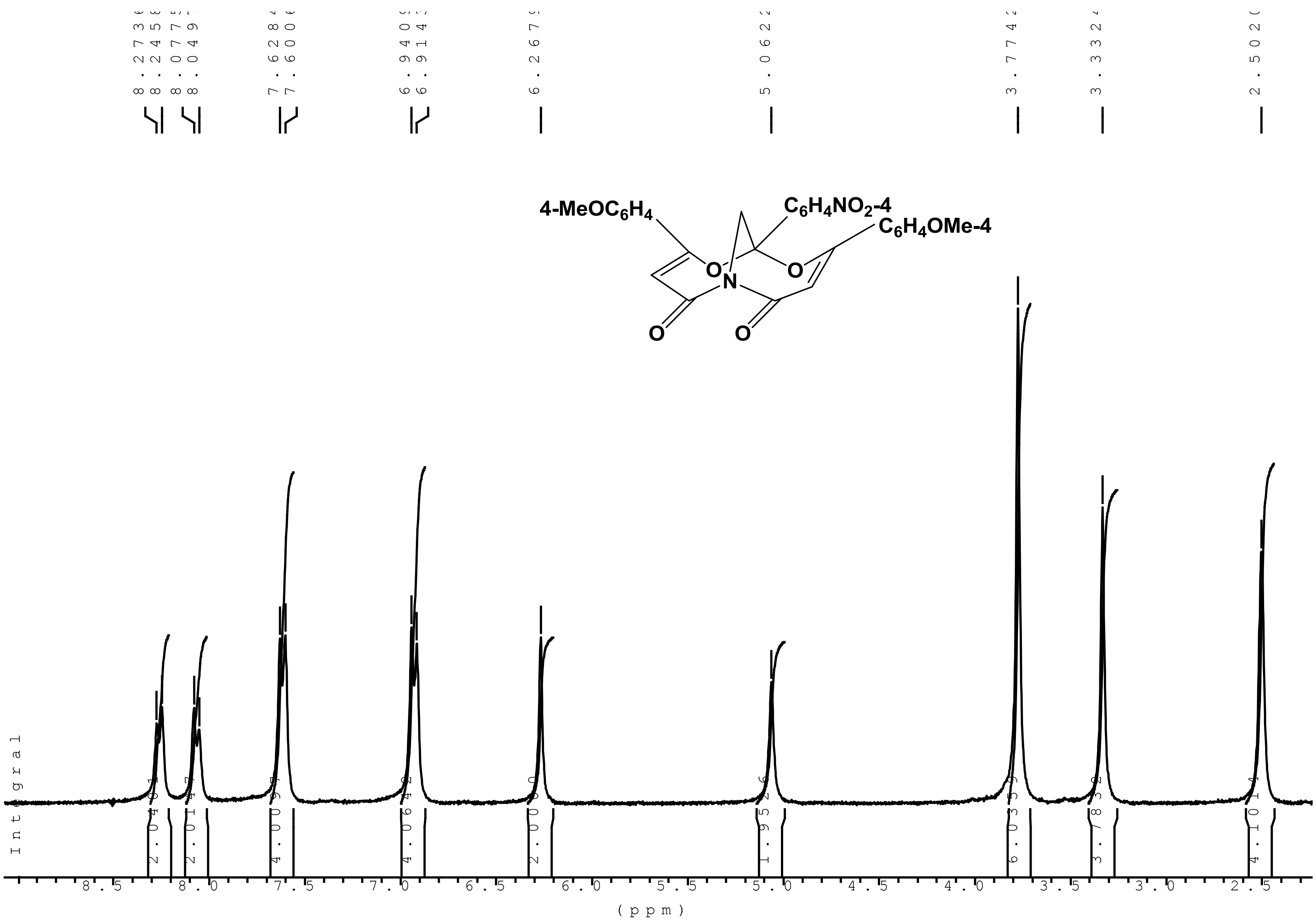

^{1}H NMR spectrum of compound **3f**.

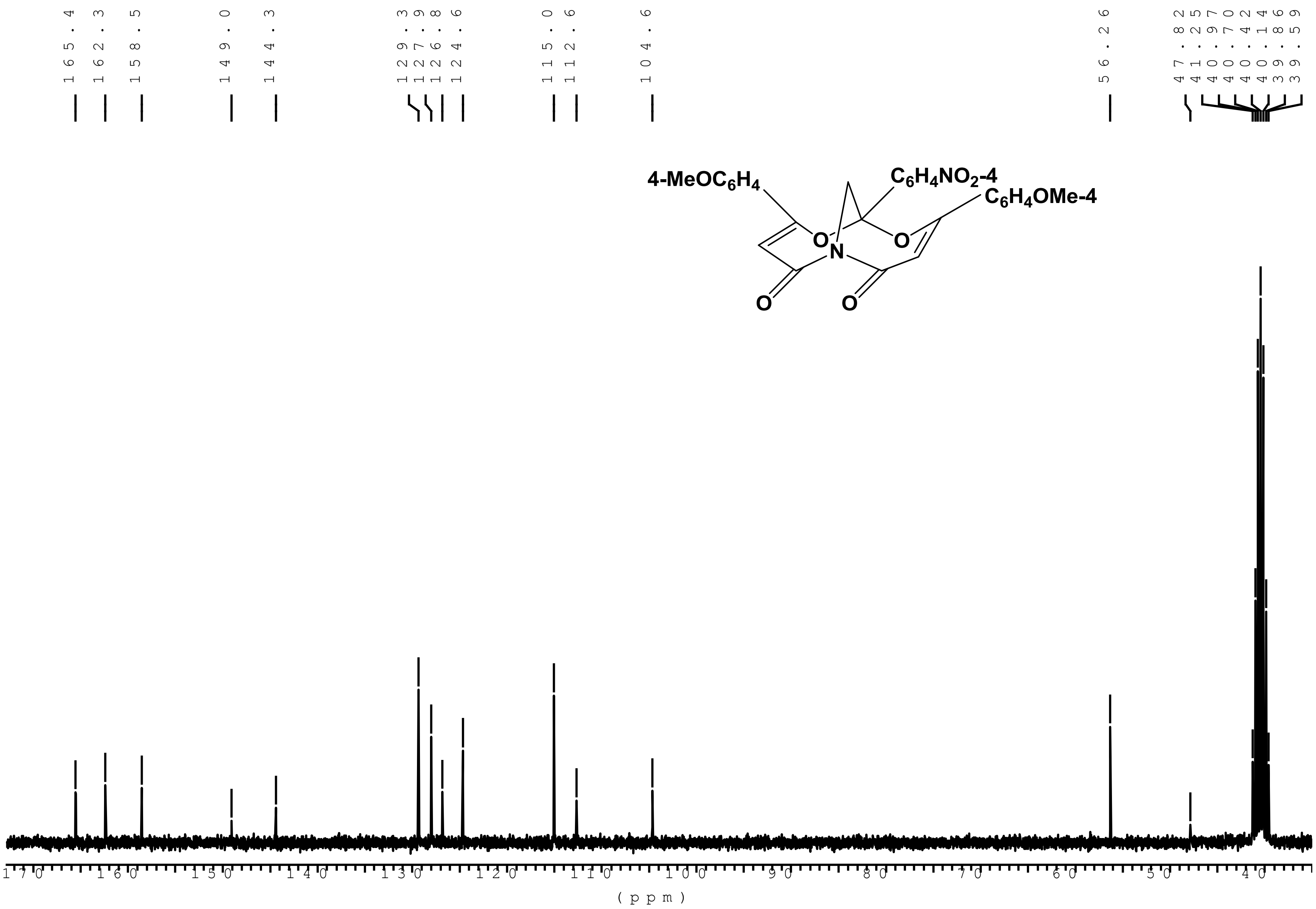

^{13}C NMR spectrum of compound **3f**.

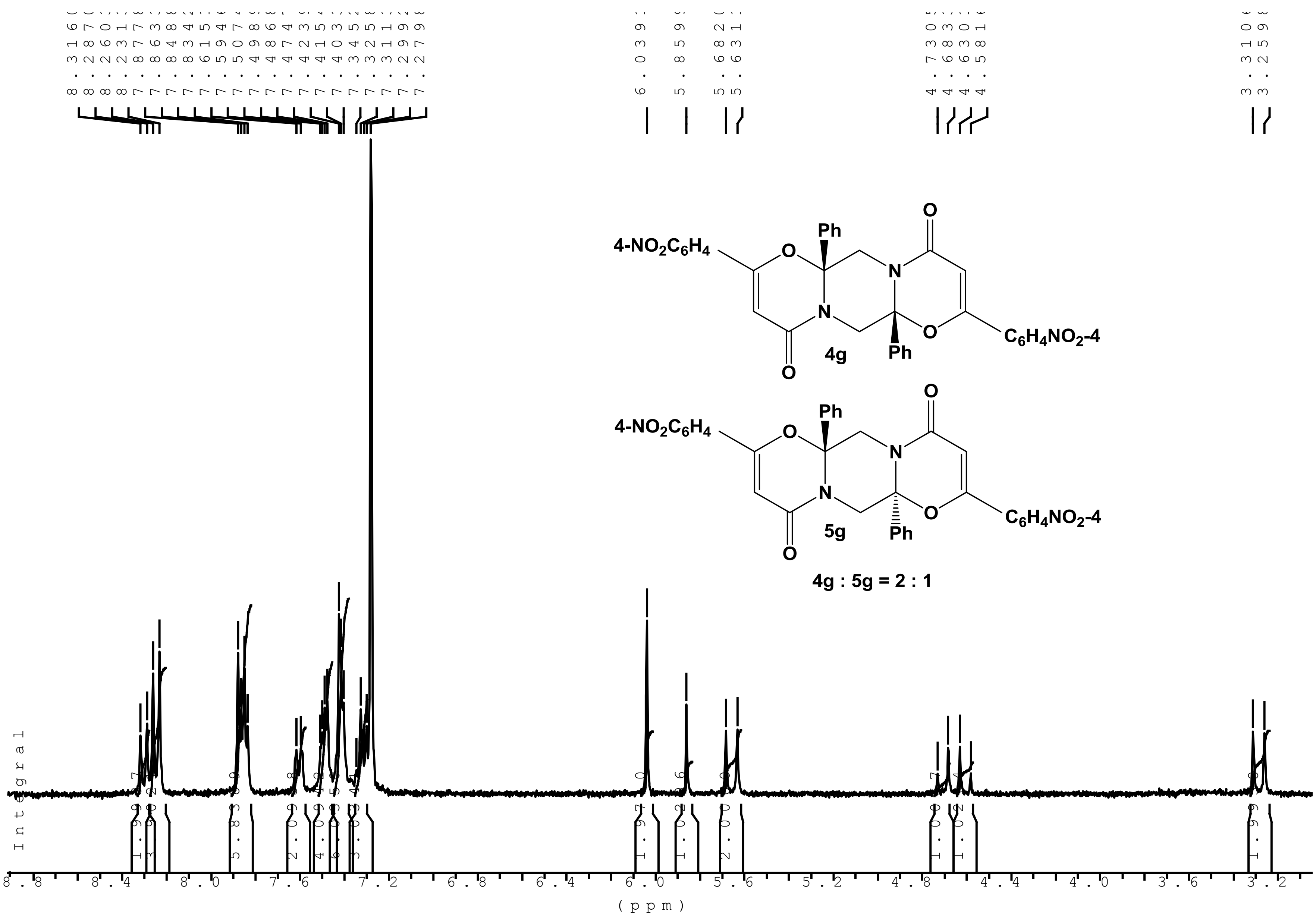

^{1}H NMR spectrum of compounds **4g, 5g (2 : 1)**.

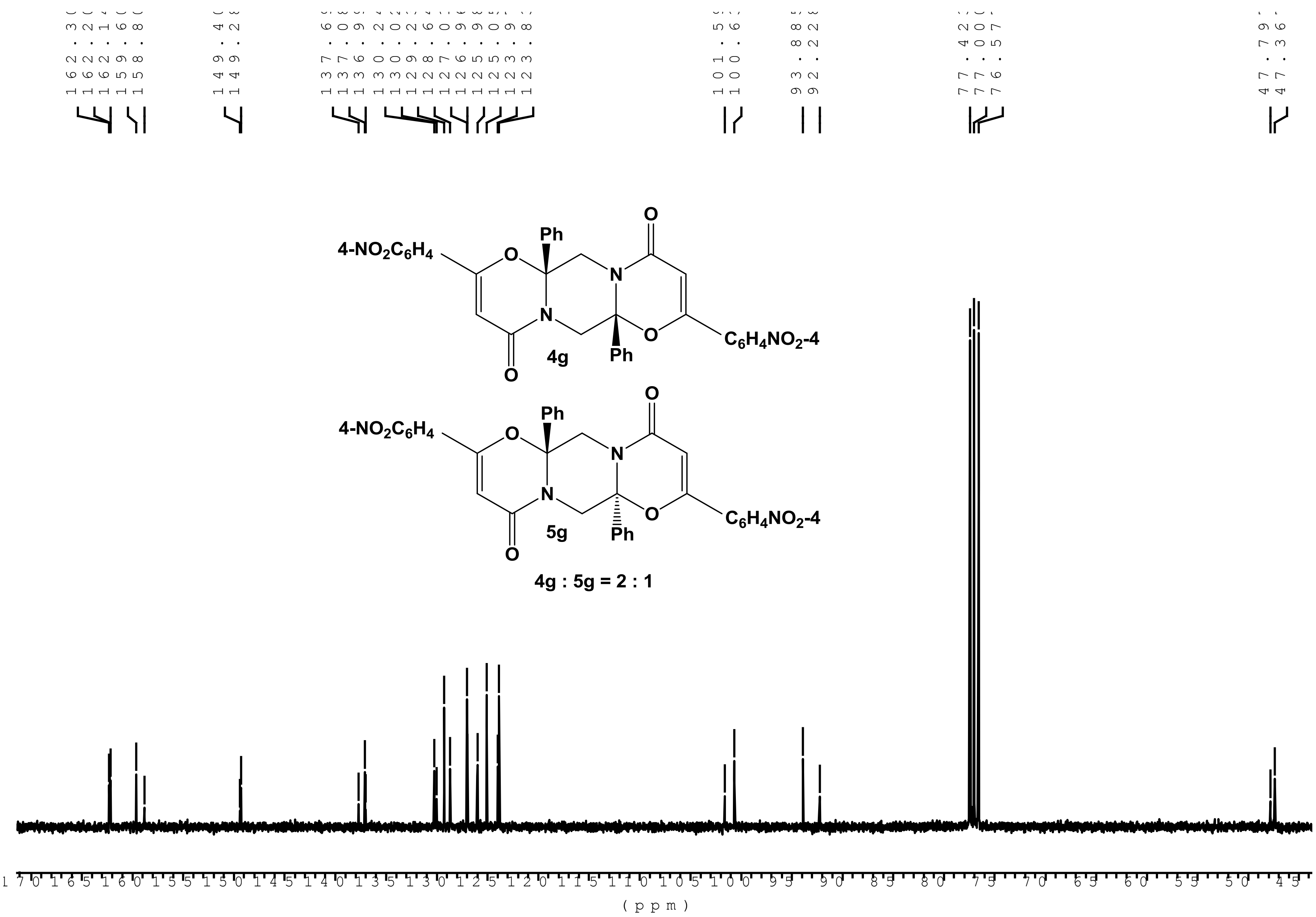

^{13}C NMR spectrum of compounds **4g, 5g (2 : 1)**.

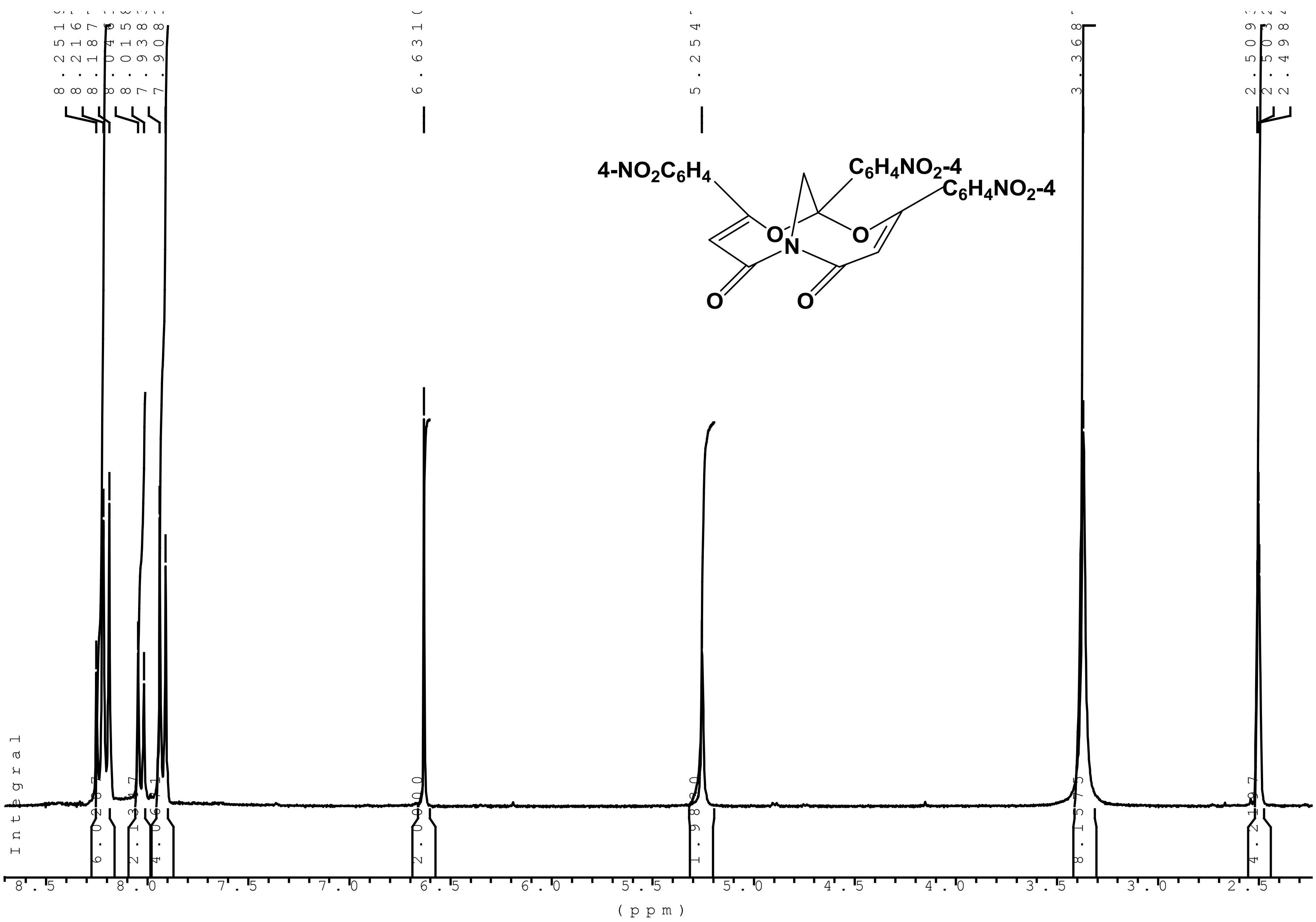

^{1}H NMR spectrum of compound **3i**.

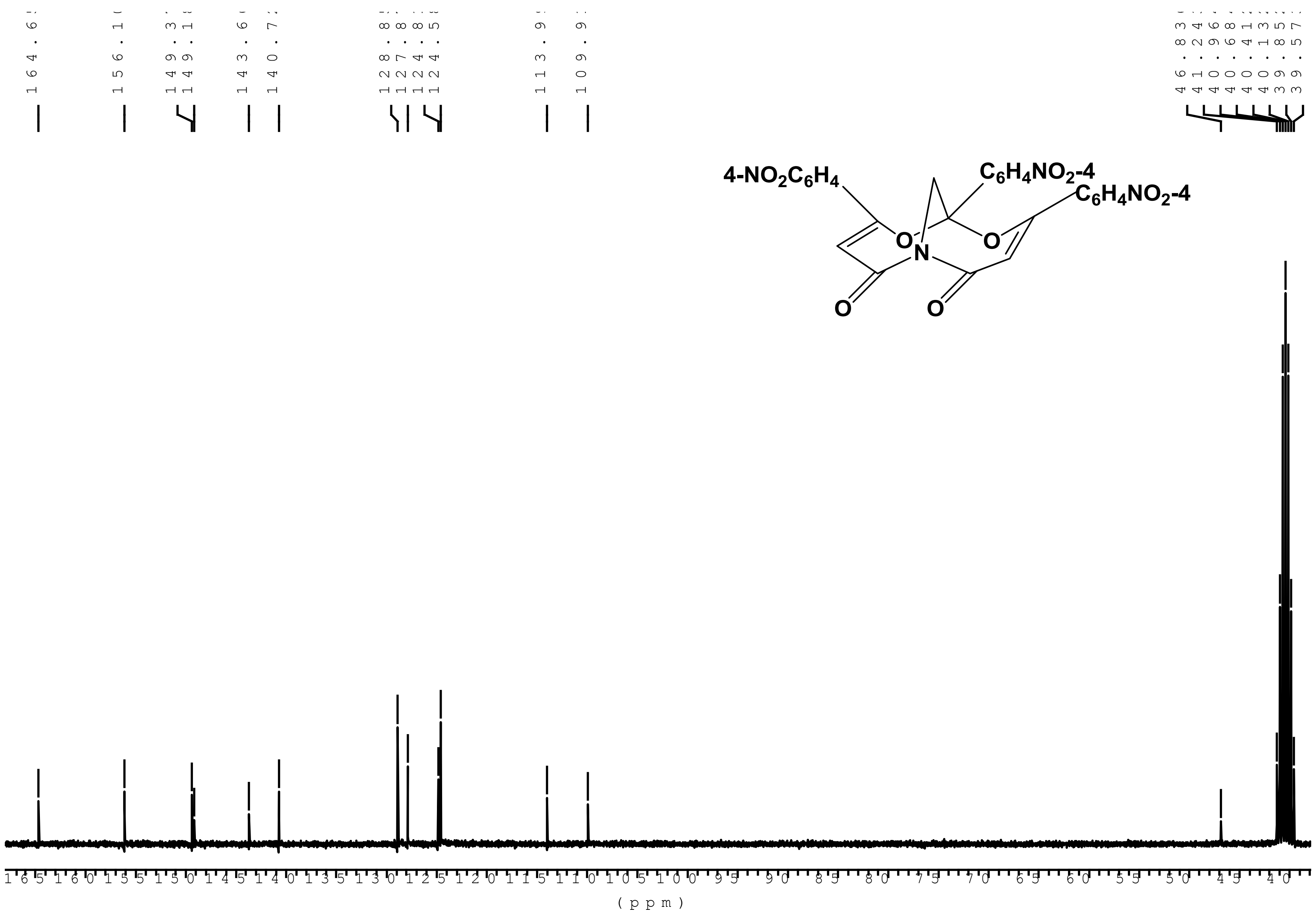

^{13}C NMR spectrum of compound **3i**.

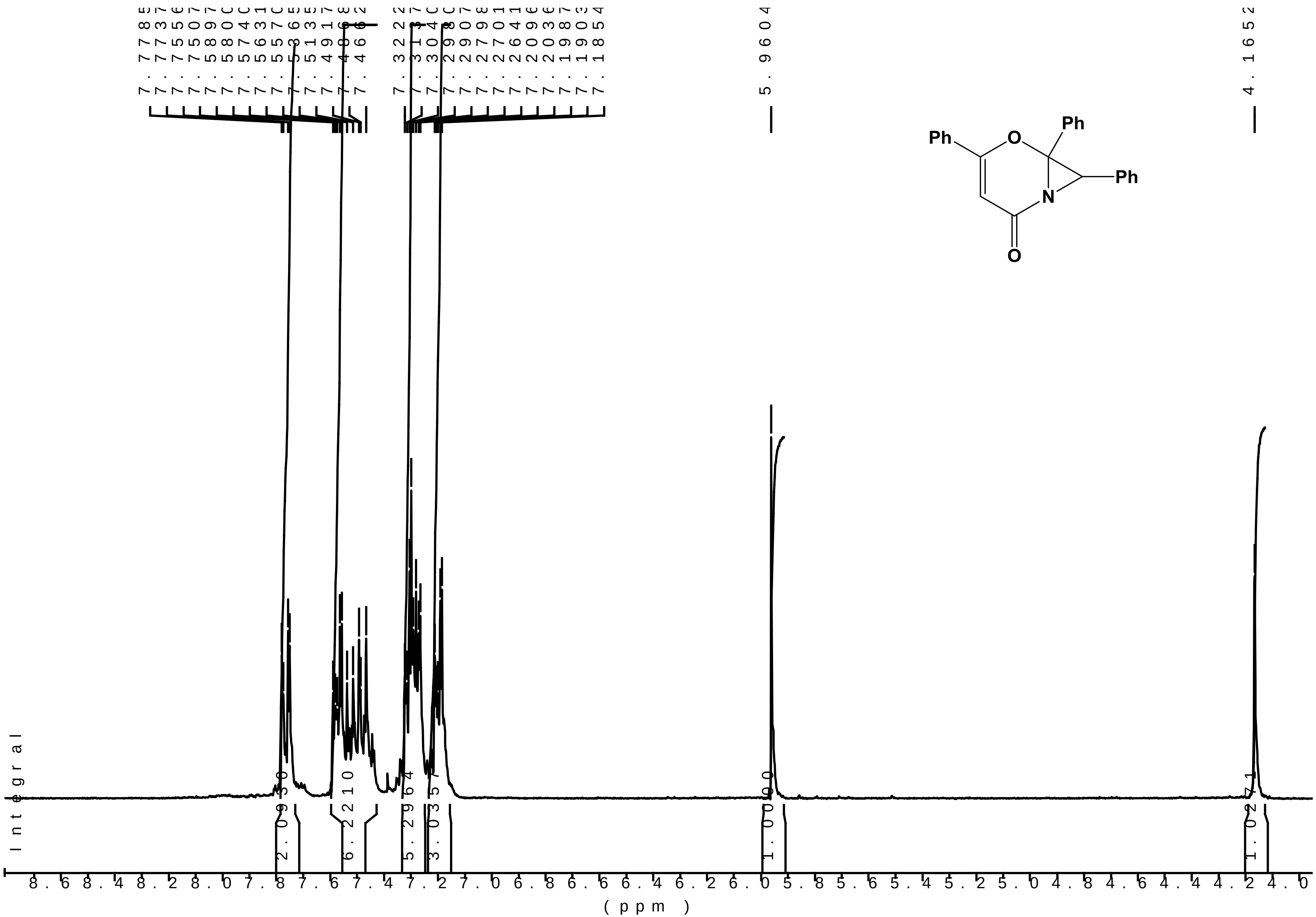

^{1}H NMR spectrum of compound **17**.

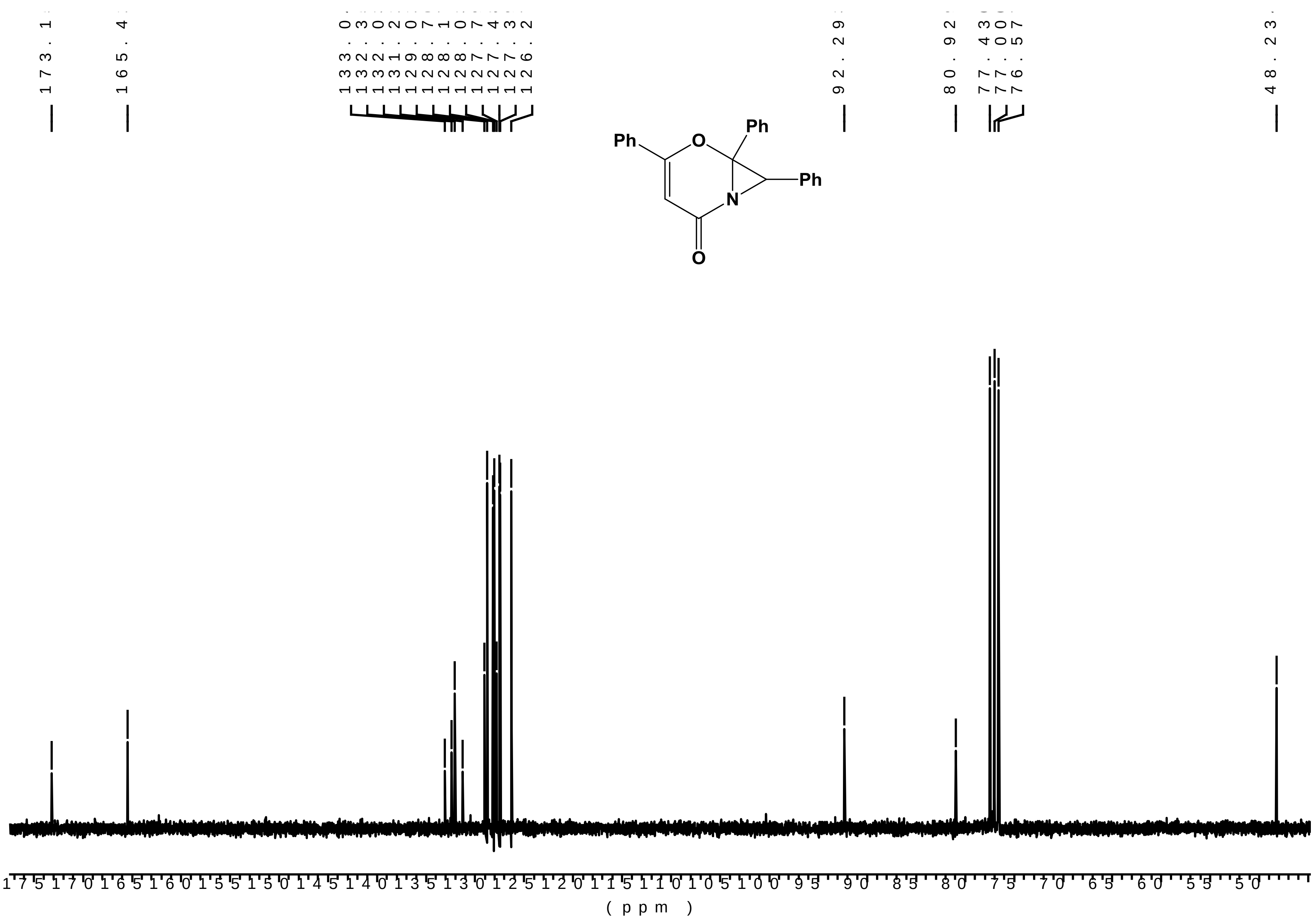

^{13}C NMR spectrum of compound **17**.

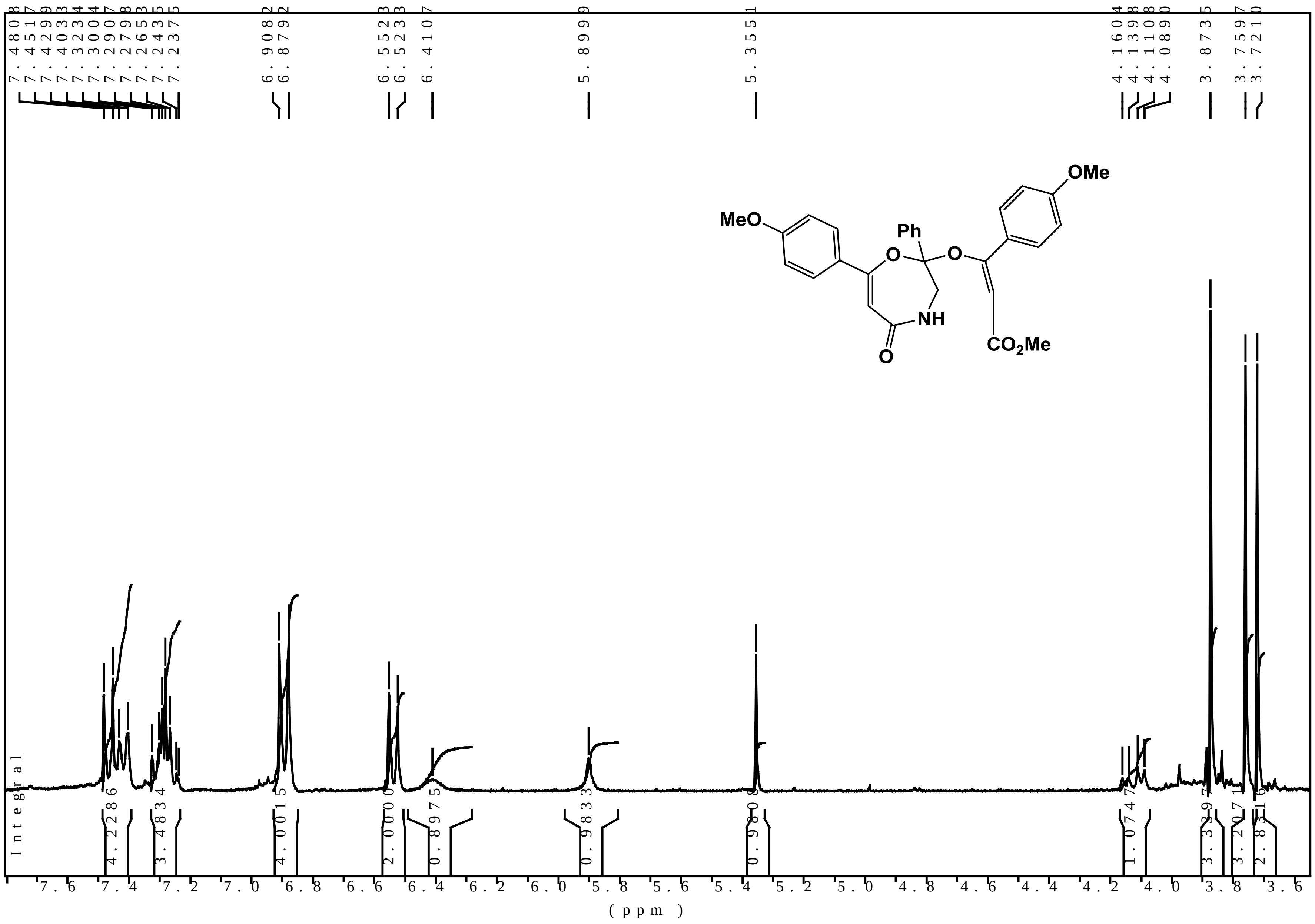

^{1}H NMR spectrum of compound **19**.

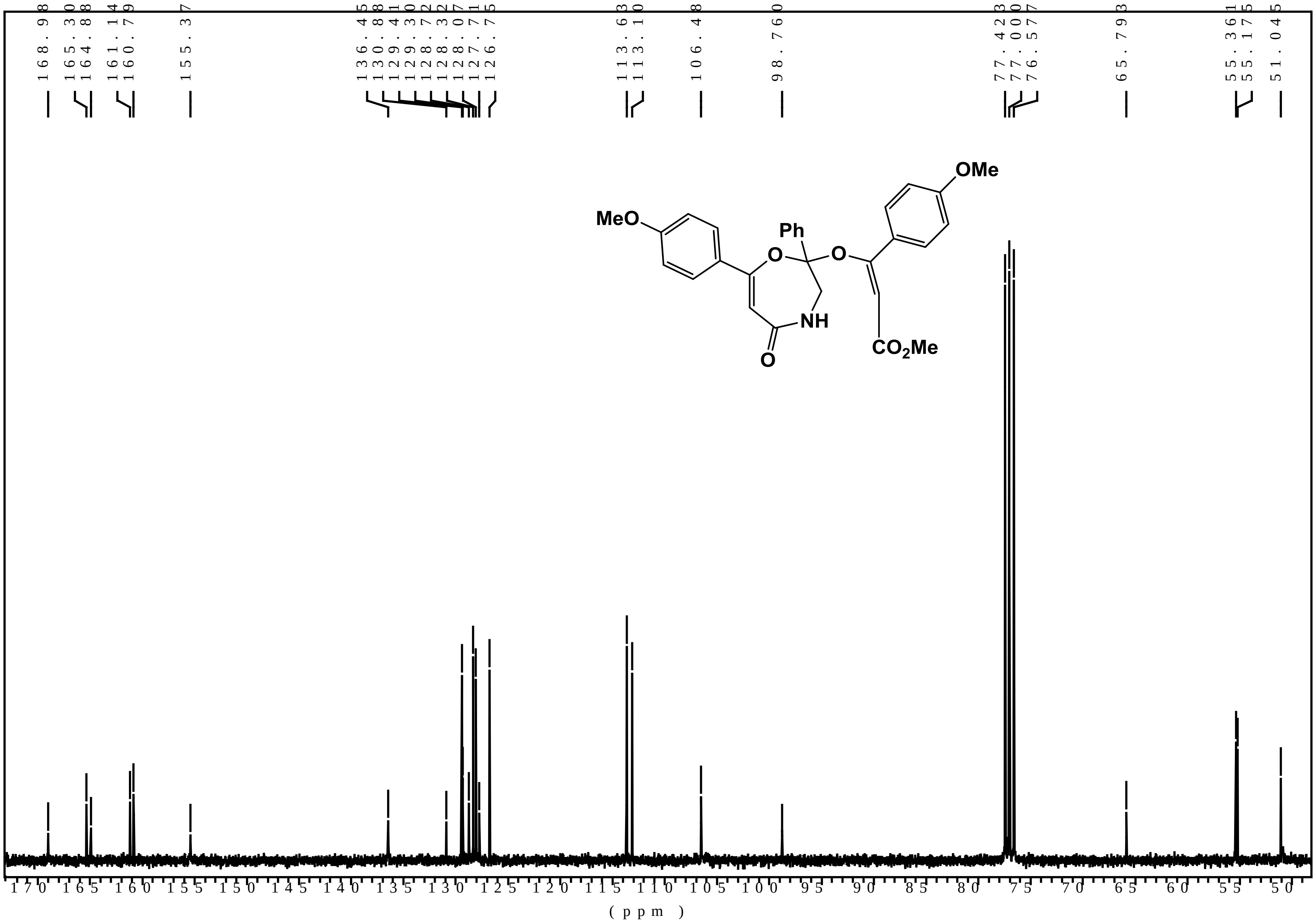

^{13}C NMR spectrum of compound **19**

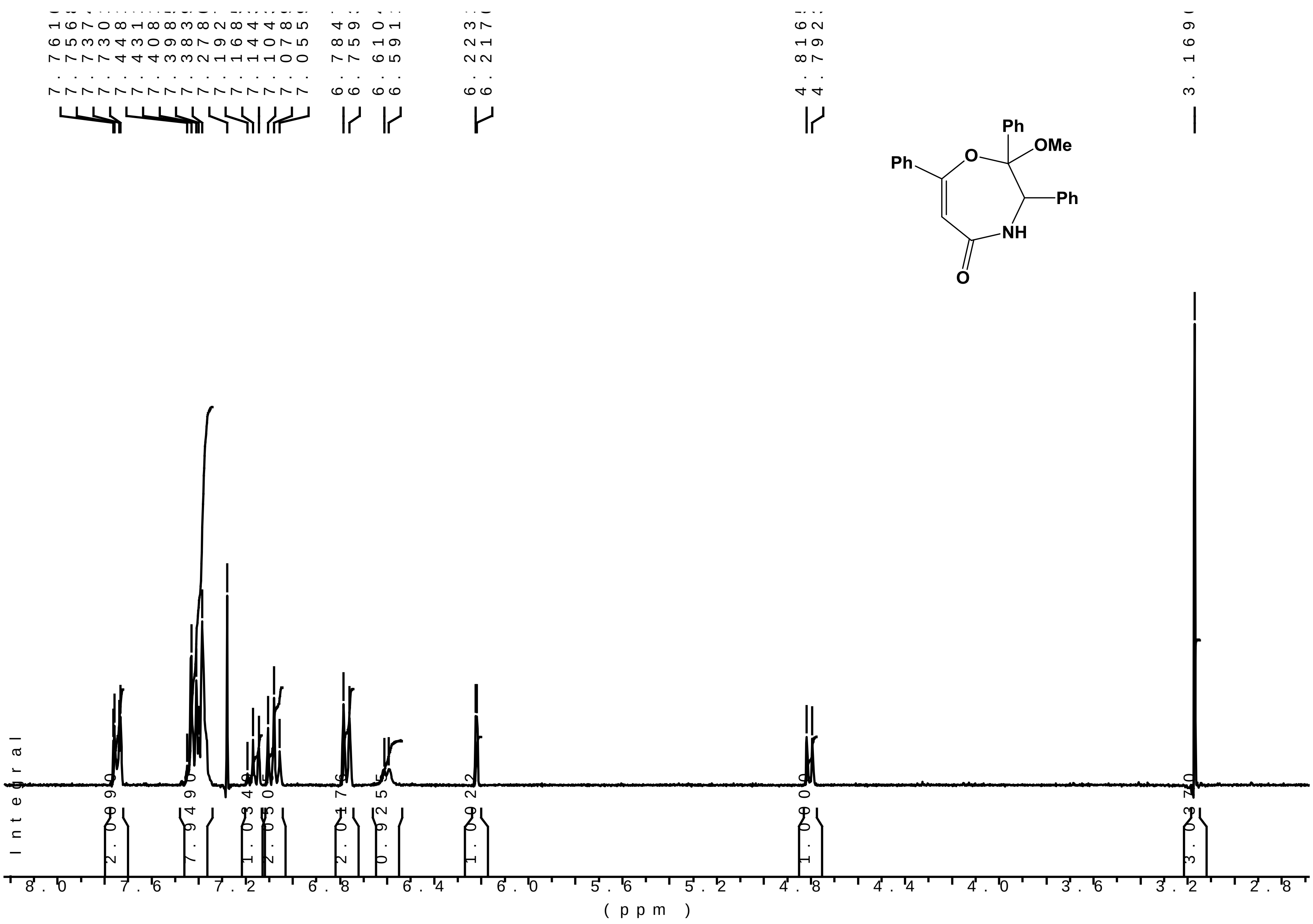

^{1}H NMR spectrum of compound **20**.

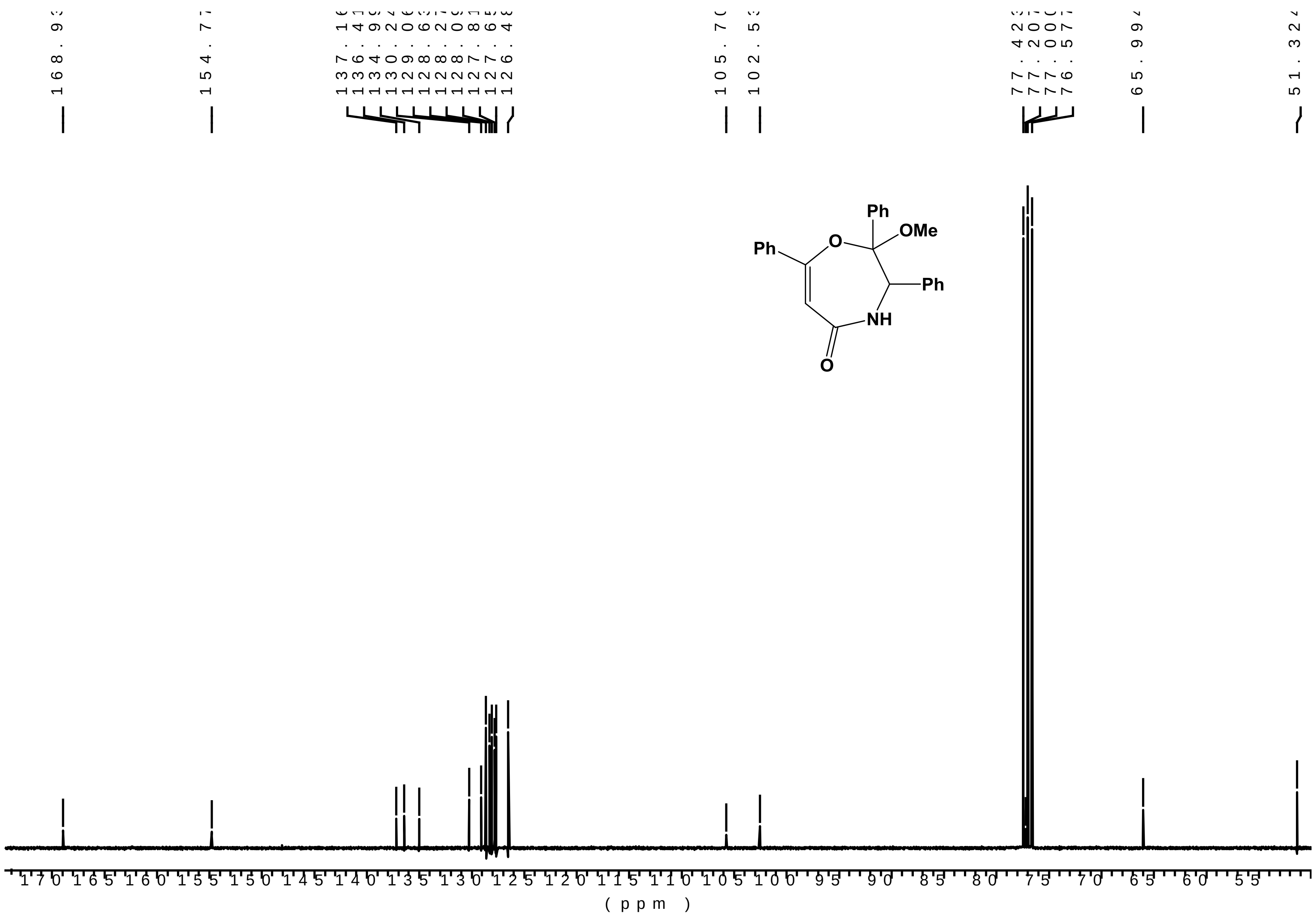

^{13}C NMR spectrum of compound **20**.

Computational Details

All calculations were performed with the B3LYP density functional method[1] by using the Gaussian 09 suite of quantum chemical programs[2] at Resource center "Computer center of Saint Petersburg State University". Geometry optimizations of molecules in the gas phase were performed at the B3LYP/6-31G(d) level.

Table. B3LYP/6-31G(d) Absolute Energies (au), Cartesian Coordinates of stationary points

Compound **2a**

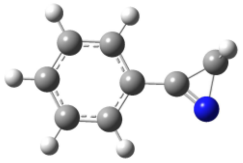

E = -363.75292788, **H (0K)** = -363.625374,
H (353K) = -363.614374, **G (353K)** = -363.665087 au.
Imaginary frequency = 0.

C	2.9048460	0.6259840	0.0309180
C	1.6374760	-0.0743490	-0.0530210
N	2.6064230	-0.8889470	0.0340030
C	0.1873820	-0.0321630	-0.0419750
C	-0.4731530	1.2055910	-0.0263140
C	-1.8652360	1.2546520	0.0091550
C	-2.6048180	0.0696320	0.0279160
C	-1.9503510	-1.1668930	0.0121730
C	-0.5602530	-1.2228670	-0.0207240
H	3.2539150	1.0451160	0.9755600
H	3.3631760	1.0427300	-0.8646860
H	0.1151170	2.1187410	-0.0429540
H	-2.3734460	2.2148800	0.0216680
H	-3.6905730	0.1080980	0.0544380
H	-2.5270510	-2.0876380	0.0256590
H	-0.0414630	-2.1768200	-0.0364750

Compound **2b**

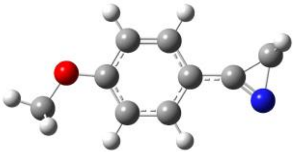

E = -478.27883949, **H (0K)** = -478.118488,
H (353K) = -478.104063, **G (353K)** = -478.162911 au.
Imaginary frequency = 0.

C	-3.8686250	0.4494180	-0.0001930
C	-2.5503330	-0.1579430	-0.0003320
N	-3.4617560	-1.0391520	-0.0005500
C	-1.1145070	-0.0089960	-0.0001630
C	-0.5322240	1.2718950	-0.0000590
C	0.8450440	1.4197210	0.0001170
C	1.6750250	0.2845650	0.0002030
C	1.1054860	-1.0001630	0.0000720
C	-0.2785800	-1.1367270	-0.0001110
H	-4.3062600	0.8361460	-0.9211410
H	-4.3062680	0.8357170	0.9209340
H	-1.1749990	2.1476330	-0.0001020
H	1.3088040	2.4008670	0.0002030
H	1.7293720	-1.8860520	0.0001040
H	-0.7239590	-2.1274220	-0.0002040
O	3.0089770	0.5334570	0.0003320
C	3.9113300	-0.5696900	0.0004530
H	4.9111130	-0.1337290	0.0006700
H	3.7833080	-1.1896460	0.8958910
H	3.7836640	-1.1895850	-0.8950820

Compound **2c**

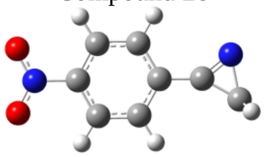

E = -568.25331808, **H (0K)** = -568.123294,
H (353K) = -568.108951, **G (353K)** = -568.168760 au.
Imaginary frequency = 0.

C	4.0747340	-0.6569550	0.0002390
C	2.8075210	0.0486430	-0.0001870
N	3.7766840	0.8610870	-0.0003680

Compound **6a**

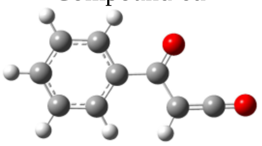

E = -496.99304962, **H (0K)** = -496.868252,
H (353K) = -496.854740, **G (353K)** = -496.912698 au.
Imaginary frequency = 0.

O	1.3219270	1.5663650	-0.1549650
C	1.8572960	-0.7125490	0.1130690
O	4.2985910	-0.2242870	0.0063180
C	3.1610930	-0.4531930	0.0568150

[1] (a) Becke, A. D. *J. Chem. Phys.* **1993**, *98*, 5648. (b) Becke, A. D. *Phys. Rev. A* **1998**, *38*, 3098. (c) Lee, C.; Yang, W.; Parr, R. G. *Phys. Rev. B* **1998**, *37*, 785.

[2] Gaussian 09, Revision C.01, M. J. Frisch, G. W. Trucks, H. B. Schlegel, G. E. Scuseria, M. A. Robb, J. R. Cheeseman, G. Scalmani, V. Barone, B. Mennucci, G. A. Petersson, H. Nakatsuji, M. Caricato, X. Li, H. P. Hratchian, A. F. Izmaylov, J. Bloino, G. Zheng, J. L. Sonnenberg, M. Hada, M. Ehara, K. Toyota, R. Fukuda, J. Hasegawa, M. Ishida, T. Nakajima, Y. Honda, O. Kitao, H. Nakai, T. Vreven, J. A. Montgomery, Jr., J. E. Peralta, F. Ogliaro, M. Bearpark, J. J. Heyd, E. Brothers, K. N. Kudin, V. N. Staroverov, T. Keith, R. Kobayashi, J. Normand, K. Raghavachari, A. Rendell, J. C. Burant, S. S. Iyengar, J. Tomasi, M. Cossi, N. Rega, J. M. Millam, M. Klene, J. E. Knox, J. B. Cross, V. Bakken, C. Adamo, J. Jaramillo, R. Gomperts, R. E. Stratmann, O. Yazyev, A. J. Austin, R. Cammi, C. Pomelli, J. W. Ochterski, R. L. Martin, K. Morokuma, V. G. Zakrzewski, G. A. Voth, P. Salvador, J. J. Dannenberg, S. Dapprich, A. D. Daniels, O. Farkas, J. B. Foresman, J. V. Ortiz, J. Cioslowski, and D. J. Fox, Gaussian, Inc., Wallingford CT, **2010**.

C	1.3567980	0.0313800	-0.0001260
C	0.6737360	-1.1943560	-0.0001320
C	-0.7171370	-1.2201030	-0.0000520
C	-1.4035050	-0.0070020	0.0000460
C	-0.7493370	1.2265550	0.0000580
C	0.6392470	1.2408340	-0.0000340
H	4.4844030	-1.0682870	-0.9216600
H	4.4841510	-1.0676260	0.9225470
H	1.2383280	-2.1213510	-0.0001960
H	-1.2697570	-2.1509280	-0.0000580
H	-1.3279020	2.1415770	0.0001400
H	1.1790710	2.1823620	-0.0000220
N	-2.8753430	-0.0273970	0.0001380
O	-3.4643490	1.0537080	0.0001880
O	-3.4344040	-1.1244020	0.0000610

C	0.9158230	0.4108190	-0.0317720
C	-0.5519830	0.1065310	-0.0123950
C	-1.4338560	1.1967340	0.0643150
C	-1.0789130	-1.1918940	-0.0800040
C	-2.8102980	0.9925210	0.0845880
H	-1.0141950	2.1959750	0.1084130
C	-2.4585820	-1.3957030	-0.0660930
H	-0.4240700	-2.0535030	-0.1634820
C	-3.3263100	-0.3052360	0.0199770
H	-3.4825760	1.8435130	0.1498300
H	-2.8548450	-2.4054760	-0.1246250
H	-4.4009050	-0.4656780	0.0342570
H	1.5668290	-1.7396400	0.2937810

TS **2a+6a → 7a**

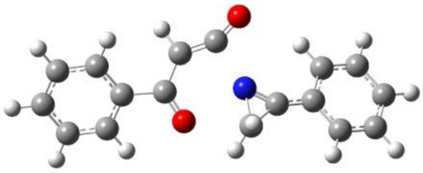

E = 860.73921931, **H (0K)** = -860.485087,
H (353K) = -860.460777, **G (353K)** = -860.546357 au.
Imaginary frequency = 1.

C	2.1066470	-0.4369800	0.8847240
N	0.9744890	0.1104590	1.0205480
C	1.1992940	-1.1996500	1.7463340
C	3.3800780	-0.3179320	0.2162640
C	4.3227690	-1.3503590	0.3514420
C	5.5542820	-1.2542250	-0.2917120
C	5.8495860	-0.1282310	-1.0647710
C	4.9149700	0.9051810	-1.1967510
C	3.6810670	0.8166260	-0.5607950
O	-1.1590820	-1.0458200	-0.0778130
C	-1.8312460	-0.0093050	-0.0490260
C	-1.2922540	1.3363310	0.0530070
C	-0.0317770	1.7589760	0.3141810
O	0.7772460	2.6178290	0.3709750
H	4.0787720	-2.2187950	0.9562710
H	6.2827840	-2.0531780	-0.1905190
H	6.8112290	-0.0533360	-1.5648010
H	5.1520150	1.7794460	-1.7958130
H	2.9506310	1.6148700	-0.6477750
C	-3.3323340	-0.1150100	-0.1782320
C	-3.8598590	-1.2966190	-0.7209730
C	-4.2165800	0.8875510	0.2469970
C	-5.2355720	-1.4642760	-0.8569070
H	-3.1683010	-2.0729660	-1.0313590
C	-5.5959590	0.7163750	0.1213750
H	-3.8336840	1.7943210	0.7052920
C	-6.1084490	-0.4563690	-0.4364950
H	-5.6297210	-2.3804310	-1.2888350
H	-6.2696870	1.4971740	0.4640460
H	-7.1825790	-0.5869290	-0.5386470
H	0.6563280	-2.0545670	1.3509310
H	1.2917720	-1.1394680	2.8301640
H	-1.9342810	2.1720770	-0.2100440

TS **2c+6a → 7c**

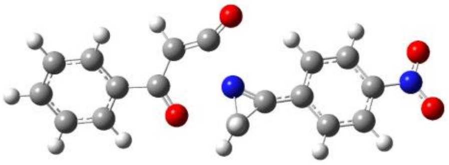

E = -1065.23753660, **H (0K)** = -1064.980821,
H (353K) = -1064.953262, **G (353K)** = -1065.047583 au.
Imaginary frequency = 1.

C	0.9542920	-0.5897430	1.1846770
N	-0.1921280	-0.0629440	1.2441610
C	-0.0253620	-1.4193490	1.8949880
C	2.2838350	-0.3987470	0.6493480
C	3.2197960	-1.4403480	0.7589820
C	4.5021980	-1.2817350	0.2457380
C	4.8265640	-0.0720930	-0.3657770
C	3.9181100	0.9811050	-0.4805990
C	2.6373990	0.8155520	0.0308040
O	-2.2155060	-1.0819080	-0.0574260
C	-2.8671940	-0.0302210	-0.0100700
C	-2.3121860	1.2789600	0.2782060
C	-1.0674860	1.6144610	0.7059090
O	-0.2676080	2.4624470	0.9255020
H	2.9354860	-2.3695880	1.2419320
H	5.2429650	-2.0682370	0.3124940
H	4.2210650	1.9030350	-0.9604240
H	1.9114800	1.6193130	-0.0321570
C	-4.3437830	-0.0799290	-0.3163310
C	-4.8294680	-1.1961220	-1.0145530
C	-5.2497810	0.9116280	0.0890870
C	-6.1833250	-1.3094700	-1.3199280
H	-4.1231520	-1.9650450	-1.3096800
C	-6.6083470	0.7939100	-0.2070110
H	-4.9035730	1.7663680	0.6621610
C	-7.0773040	-0.3129290	-0.9172410
H	-6.5441170	-2.1742280	-1.8705350
H	-7.3005920	1.5645250	0.1213130
H	-8.1347520	-0.4012290	-1.1520760
H	-0.5204210	-2.2420380	1.3864130
H	-0.0389700	-1.4333020	2.9835480
H	-2.9022350	2.1594740	0.0443680
N	6.1856530	0.1028330	-0.9104390
O	6.4504560	1.1747220	-1.4526320
O	6.9744550	-0.8337420	-0.7905430

Compound **7a**

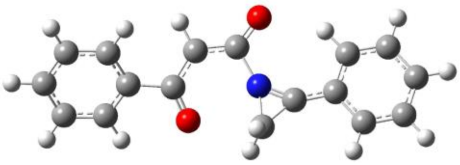

E = -860.74698270, **H (0K)** = -860.490434,
H (353K) = -860.466359, **G (353K)** = -860.550202 au.
Imaginary frequency = 0.

C	2.0896370	-0.5815900	0.3629080
N	0.9065600	-0.1176770	0.2640800
C	1.0188210	-1.4754800	0.8618270
C	3.4686610	-0.3110400	0.1160910
C	4.4159090	-1.3228050	0.3737540
C	5.7632230	-1.0771220	0.1381890
C	6.1673530	0.1718330	-0.3467550
C	5.2290640	1.1804330	-0.5977930
C	3.8777420	0.9495420	-0.3716300
O	-1.4570040	-1.2717550	-0.4665270
C	-1.9608940	-0.1584880	-0.2092060
C	-1.2140970	1.0560460	-0.0303170
C	0.1708390	1.1810500	0.0556640
O	0.9162490	2.1621700	0.0345900
H	4.0837390	-2.2857150	0.7500640
H	6.4988680	-1.8516880	0.3310420
H	7.2215490	0.3610590	-0.5285930
H	5.5564550	2.1466500	-0.9690290
H	3.1259740	1.7142220	-0.5407920
C	-3.4673920	-0.0674380	-0.1165270
C	-4.2231800	-1.1066040	-0.6803520
C	-4.1414970	0.9808140	0.5285170
C	-5.6147480	-1.0912240	-0.6202730
H	-3.6921800	-1.9206560	-1.1628160
C	-5.5353190	0.9940310	0.5974110
H	-3.5785050	1.7802400	1.0002800
C	-6.2761890	-0.0386850	0.0190650
H	-6.1858390	-1.8993460	-1.0704130
H	-6.0422540	1.8093120	1.1071410
H	-7.3619330	-0.0256880	0.0694770
H	0.6337170	-2.2845830	0.2505770
H	0.8493280	-1.5476980	1.9352450
H	-1.7363980	2.0046860	-0.0486760

Compound **7c**

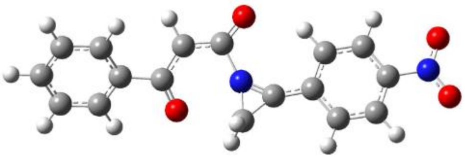

E = -1065.24272864, **H (0K)** = -1064.984096,
H (353K) = -1064.956627, **G (353K)** = -1065.049577 au.
Imaginary frequency = 0.

C	0.9432860	-0.6993980	0.4940450
N	-0.2184370	-0.1921950	0.3888930
C	-0.1610730	-1.5822290	0.9308490
C	2.3384320	-0.4506700	0.2801930
C	3.2541270	-1.4993400	0.4937530
C	4.6108240	-1.2834560	0.2918280
C	5.0264350	-0.0160410	-0.1160000
C	4.1401120	1.0409730	-0.3276380
C	2.7828070	0.8241080	-0.1299800
O	-2.5260350	-1.2130570	-0.5492350
C	-3.0356780	-0.1194280	-0.2287270
C	-2.2797040	1.0696260	0.0650500
C	-0.8992860	1.1500090	0.2221730
O	-0.1175490	2.0980970	0.2946510
H	2.8967420	-2.4734110	0.8113720
H	5.3409340	-2.0679030	0.4438130
H	4.5184380	2.0064000	-0.6387230
H	2.0547430	1.6177650	-0.2654560
C	-4.5407890	-0.0201850	-0.1812490
C	-5.2853930	-1.0099000	-0.8412030
C	-5.2261460	0.9894450	0.5119960
C	-6.6777840	-0.9826920	-0.8263660
H	-4.7461170	-1.7939760	-1.3624520
C	-6.6211130	1.0135160	0.5348420
H	-4.6727560	1.7480190	1.0567260
C	-7.3505630	0.0311390	-0.1380190
H	-7.2407300	-1.7509310	-1.3502040
H	-7.1380050	1.7974390	1.0820800
H	-8.4371360	0.0531810	-0.1233960
H	-0.5575550	-2.3486550	0.2736160
H	-0.3588240	-1.6923810	1.9957840
H	-2.7827350	2.0284340	0.0861000
N	6.4690840	0.2164820	-0.3300210
O	6.8170750	1.3371350	-0.6962730
O	7.2336980	-0.7255320	-0.1284750

TS **7a** → **8a**

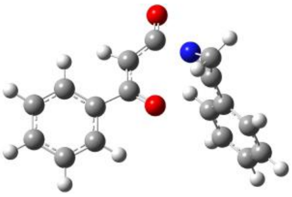

E = -860.73862274, **H (0K)** = -860.482904,
H (353K) = -860.459526, **G (353K)** = -860.541514 au.
Imaginary frequency = 1.

C	1.9925290	0.9661090	0.7661830
N	1.2126290	1.9283930	0.4903950
C	1.5192290	1.7166850	1.9435150
C	2.7352730	-0.1106960	0.1907110

TS **7c** → **8c**

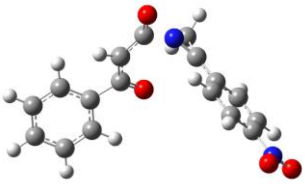

E = -1065.23396640, **H (0K)** = -1064.976297,
H (353K) = -1064.949371, **G (353K)** = -1065.041193 au.
Imaginary frequency = 1.

C	-0.7247060	1.8757010	0.8315720
N	0.2555140	2.5010560	0.3350950
C	0.0872350	2.5930610	1.8288890

C	3.5043320	-0.9367990	1.0298430
C	4.2285230	-1.9894110	0.4805020
C	4.1904780	-2.2132510	-0.8992710
C	3.4290350	-1.3894880	-1.7375940
C	2.6992060	-0.3372300	-1.1997040
O	-0.4538490	-0.1745230	1.0061710
C	-1.2600010	0.3945990	0.2340060
C	-1.0020150	1.5748300	-0.5386480
C	0.1294530	2.3887620	-0.5584340
O	0.4511440	3.3674300	-1.2051940
H	3.5195490	-0.7480410	2.0987880
H	4.8210540	-2.6342690	1.1217860
H	4.7581970	-3.0351790	-1.3260480
H	3.4063720	-1.5732580	-2.8071270
H	2.1013530	0.3073480	-1.8362610
C	-2.6272480	-0.2362910	0.0784460
C	-2.7649860	-1.5890100	0.4258690
C	-3.7628200	0.4622820	-0.3595880
C	-3.9943850	-2.2345000	0.3141500
H	-1.8871500	-2.1164380	0.7841100
C	-4.9981730	-0.1792890	-0.4609060
H	-3.6942620	1.5189650	-0.5983760
C	-5.1168950	-1.5309100	-0.1313190
H	-4.0800890	-3.2859650	0.5768410
H	-5.8693910	0.3795660	-0.7927780
H	-6.0784480	-2.0308780	-0.2147690
H	0.7366170	1.2587700	2.5416890
H	2.1396380	2.4840590	2.3998120
H	-1.7494180	1.8949640	-1.2548170

C	-1.8059660	0.9935110	0.4878790
C	-2.6303340	0.4922620	1.5089700
C	-3.6777700	-0.3650460	1.1928690
C	-3.8823680	-0.6943550	-0.1456150
C	-3.0818190	-0.2050140	-1.1785480
C	-2.0334340	0.6466520	-0.8574150
O	1.3563590	0.1286310	1.0670590
C	2.1946860	0.3287620	0.1589640
C	2.1540540	1.3888360	-0.8101240
C	1.2706840	2.4559120	-0.9176420
O	1.0845510	3.3470140	-1.7173300
H	0.7784540	2.0059610	2.4261340
H	-2.4418710	0.7700360	2.5405610
H	-4.3287160	-0.7734910	1.9550130
H	-3.2856540	-0.4930970	-2.2018390
H	-1.3938890	1.0456320	-1.6376450
C	3.3407290	-0.6509490	0.0462480
C	3.1833420	-1.9120620	0.6416630
C	4.5570210	-0.3541860	-0.5878220
C	4.2015670	-2.8607910	0.5844380
H	2.2476680	-2.1266440	1.1472110
C	5.5824770	-1.2994590	-0.6364260
H	4.7202940	0.6268270	-1.0228320
C	5.4060320	-2.5572950	-0.0565390
H	4.0589540	-3.8368360	1.0412020
H	6.5218290	-1.0502730	-1.1230060
H	6.2038380	-3.2942330	-0.0984780
H	2.8574370	1.3586720	-1.6339100
H	-0.2232600	3.5677650	2.1960730
N	-4.9997490	-1.5999780	-0.4863040
O	-5.1676720	-1.8747980	-1.6723200
O	-5.6924900	-2.0209860	0.4378170

Compound **8a**

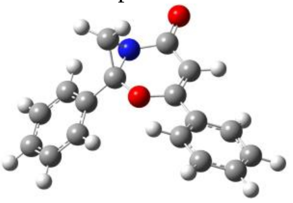

E = -860.78937450, **H (0K)** = -860.530587,
H (353K) = -860.508041, **G (353K)** = -860.587771 au.
Imaginary frequency = 0.

C	-1.1916200	0.5807250	0.6096890
N	-1.3668110	1.9485990	0.1756030
C	-1.5403880	1.6433410	1.6065640
C	-2.1767640	-0.4492780	0.1352550
C	-1.8965640	-1.8165000	0.2476200
C	-2.8388240	-2.7616930	-0.1607330
C	-4.0669760	-2.3523860	-0.6825150
C	-4.3476090	-0.9890380	-0.7982300
C	-3.4085810	-0.0412850	-0.3926570
O	0.1266270	0.0487630	0.6821030
C	1.1234850	0.6782590	0.0016090
C	0.9710180	1.9272680	-0.5116810
C	-0.2352840	2.7022610	-0.2686150
O	-0.3265820	3.9091210	-0.4189320
H	-0.7518360	1.9555260	2.2896130
H	-0.9402420	-2.1396180	0.6439110
H	-2.6088850	-3.8201140	-0.0726250
H	-4.7986480	-3.0899410	-1.0008540

Compound **8b**

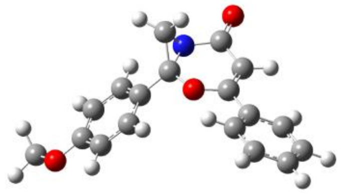

E = -975.31314693, **H (0K)** = -975.021603,
H (353K) = -974.995594, **G (353K)** = -975.083462 au.
Imaginary frequency = 0.

C	-0.2915890	1.0809070	0.7161930
N	-0.1598450	2.4163910	0.1808080
C	-0.2703810	2.2571960	1.6434600
C	-1.5353240	0.3045610	0.3989180
C	-1.6352040	-1.0604320	0.7102020
C	-2.8053170	-1.7620240	0.4527780
C	-3.9093760	-1.1127940	-0.1209380
C	-3.8195220	0.2481660	-0.4375890
C	-2.6363370	0.9423790	-0.1767270
O	0.8555340	0.2343320	0.7389600
C	1.9157760	0.5489010	-0.0525760
C	2.0334070	1.7611830	-0.6561330
C	1.0788410	2.8284830	-0.4032260
O	1.2733190	4.0064690	-0.6537700
H	0.6310570	2.4046620	2.2364540
H	-0.7880320	-1.5786930	1.1466600
H	-2.8874060	-2.8189010	0.6869430
H	-4.6521570	0.7734660	-0.8909080

H	-5.2970530	-0.6605260	-1.2122950
H	-3.6155960	1.0190350	-0.4982000
C	2.3634940	-0.1196920	-0.0435330
C	2.5601860	-1.1744090	0.8643750
C	3.3657580	0.1587730	-0.9893050
C	3.7366250	-1.9207900	0.8345710
H	1.7942330	-1.3971140	1.5984690
C	4.5370480	-0.5928310	-1.0180070
H	3.2202650	0.9480940	-1.7195620
C	4.7284840	-1.6333000	-0.1047880
H	3.8780750	-2.7283610	1.5472980
H	5.2988070	-0.3704740	-1.7597010
H	5.6434710	-2.2184720	-0.1294010
H	1.8006600	2.4367050	-0.9822920
H	-2.5568390	1.7254590	1.9833270

H	-2.5631360	1.9940470	-0.4355360
C	2.9141420	-0.5360360	-0.1138580
C	2.9083520	-1.5586350	0.8502060
C	3.8844580	-0.5667510	-1.1309020
C	3.8589950	-2.5766160	0.8050220
H	2.1640430	-1.5443580	1.6381120
C	4.8290320	-1.5882000	-1.1743830
H	3.8873900	0.1955980	-1.9030690
C	4.8222080	-2.5952240	-0.2052910
H	3.8465080	-3.3564710	1.5612050
H	5.5675890	-1.6021110	-1.9707550
H	5.5604680	-3.3914070	-0.2414540
H	2.9202940	2.0143550	-1.2204820
H	-1.1952120	2.6203700	2.0845520
O	-5.0107860	-1.8886940	-0.3312800
C	-6.1608250	-1.2827480	-0.9084420
H	-5.9485070	-0.8918320	-1.9115340
H	-6.9102760	-2.0726350	-0.9795540
H	-6.5462590	-0.4715870	-0.2778530

Compound **8c**

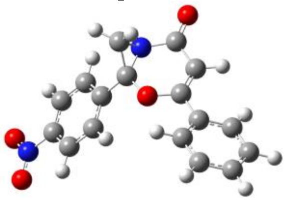

E = -1065,23396640, **H (0K)** = -1064,976297,
H (353K) = -1064,949371, **G (353K)** = -1065,041193 au.
Imaginary frequency = 0.

C	-0.0153330	1.2079350	0.6882700
N	0.2141330	2.5272540	0.1425240
C	0.0808440	2.3924210	1.6020790
C	-1.3209140	0.5355740	0.3780730
C	-1.4789570	-0.8448770	0.5622260
C	-2.7045330	-1.4537840	0.3092220
C	-3.7678840	-0.6670400	-0.1285740
C	-3.6352220	0.7071410	-0.3250490
C	-2.4056800	1.3036360	-0.0699160
O	1.0537380	0.2759400	0.7240720
C	2.1496820	0.5044680	-0.0550640
C	2.3644220	1.7002050	-0.6633400
C	1.4889020	2.8381460	-0.4371090
O	1.7664390	3.9942220	-0.7026750
H	0.9844740	2.4690260	2.2048000
H	-0.6403020	-1.4451000	0.8941400
H	-2.8431970	-2.5190910	0.4443980
H	-4.4805550	1.2849150	-0.6770770
H	-2.2727650	2.3672410	-0.2359900
C	3.0605130	-0.6540000	-0.0939190
C	2.9723110	-1.6576820	0.8862500
C	4.0295590	-0.7741560	-1.1057010
C	3.8418610	-2.7461760	0.8610120
H	2.2310600	-1.5738750	1.6728330
C	4.8926140	-1.8658560	-1.1287650
H	4.0938060	-0.0263550	-1.8893760
C	4.8042130	-2.8541890	-0.1445180
H	3.7676170	-3.5104640	1.6293040
H	5.6308250	-1.9489940	-1.9210820
H	5.4790720	-3.7051790	-0.1650830

TS **8a+6a → 9a**

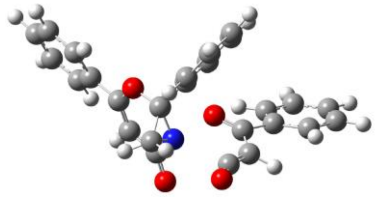

E = -1357.77811826, **H (0K)** = -1357.393226,
H (353K) = -1357.357261, **G (353K)** = -1357.469108 au.
Imaginary frequency = 1.

C	-1.1214270	1.4295390	0.5781340
O	-2.2056360	0.6587440	0.0738380
C	0.1840460	1.5979440	-1.5648000
C	-0.5224960	2.2355070	-0.5357110
H	-2.0303630	1.6392130	2.5906400
N	-0.2476650	0.8302350	1.5841300
C	-1.1740770	1.9186530	1.9807110
O	0.0886270	-0.8945780	3.0648530
O	1.2745920	-0.9280820	0.1322310
C	0.7037920	2.3476270	-2.6186100
C	-2.4535060	-0.5853910	0.5575000
C	2.7424690	0.5274670	1.3379310
O	1.7416190	2.3130260	2.7162880
C	-0.1904450	4.3688300	-1.6372560
C	-1.6884020	-1.1605440	1.5215550
C	-4.5605600	-0.4000640	-0.7699980
C	-3.8675830	-2.5838230	0.0132600
C	-5.6899000	-0.9712770	-1.3530760
H	-0.3403420	5.4445650	-1.6633230
H	-1.2692630	4.1147260	0.2108310
H	-0.6775160	2.8451160	2.2582000
C	0.5178990	3.7328000	-2.6572620
H	0.9252250	4.3134660	-3.4806070
H	1.2574180	1.8506270	-3.4104930
H	-4.3889970	0.6672560	-0.8478190
C	-4.9964980	-3.1496230	-0.5721460
C	-5.9135220	-2.3457120	-1.2548300
H	-5.1562590	-4.2217750	-0.5026830
C	-0.7112980	3.6203990	-0.5796660
H	0.3398650	0.5252560	-1.5204640

H	3.2749030	1.8811910	-1.2178430
H	-0.8177390	2.8257520	2.0341300
N	-5.0636460	-1.3036860	-0.3951640
O	-5.9904140	-0.5867110	-0.7754190
O	-5.1546370	-2.5202800	-0.2229450

H	-3.1517870	-3.2245270	0.5176040
H	-6.3960990	-0.3402600	-1.8852590
C	-3.6388410	-1.1990420	-0.0717010
C	1.9757390	1.4028300	2.0167620
C	-0.5624470	-0.4816310	2.1237300
H	-6.7932460	-2.7900300	-1.7119770
C	2.4160770	-0.5538020	0.4238760
C	3.5891240	-1.2696020	-0.2004010
C	3.3617760	-2.5564950	-0.7127960
C	4.8696690	-0.7096100	-0.3242250
C	4.3927710	-3.2757090	-1.3114920
H	2.3647930	-2.9753810	-0.6273000
C	5.9001700	-1.4247600	-0.9355750
H	5.0658890	0.2992600	0.0258710
C	5.6667290	-2.7114800	-1.4241400
H	4.2046100	-4.2761650	-1.6918560
H	6.8839930	-0.9739410	-1.0333770
H	6.4716680	-3.2700650	-1.8943370
H	3.7870530	0.6890330	1.5900110
H	-1.9321640	-2.1390920	1.9111010

TS **8c+6a → 9c**

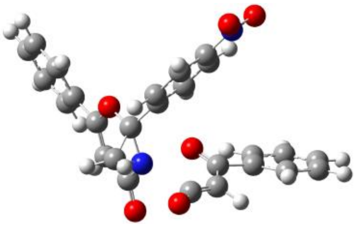

E = -1562.27981008, **H (0K)** = -1561.892260, **H (353K)** = -1561.853060, **G (353K)** = -1561.973173 au.

Imaginary frequency = 1.

C	-1.0959820	0.7771370	1.2577530
O	-2.2187240	0.5920000	0.4142320
C	0.4564890	1.4543090	-0.5978100
C	-0.1644590	1.7687280	0.6200270
H	-2.2336010	0.3831880	3.1198500
N	-0.5355310	-0.3827180	1.9495580
C	-1.2547700	0.6523950	2.7299620
O	-0.7939040	-2.5928900	2.5299470
O	0.6696180	-1.6620730	-0.1011610
C	1.2923350	2.3784000	-1.2148660
C	-2.8248000	-0.6233740	0.3252340
C	2.2356950	-1.2479580	1.6577000
O	1.4939900	-0.0140410	3.6535240
C	0.8808140	3.9558860	0.6007770
C	-2.3674510	-1.7162780	0.9888300
C	-4.6374260	0.6362970	-0.8414490
C	-4.5362170	-1.7705880	-1.0980120
C	-5.7700560	0.6715740	-1.6519600
H	1.0563470	4.9295970	1.0401060
H	-0.4440560	3.2722260	2.1432130
H	-0.6148880	1.2162080	3.4042910
C	1.4904150	3.6167340	-0.6038730
H	1.7888880	2.1539720	-2.1504840
H	-4.2365940	1.5542780	-0.4271870
C	-5.6667160	-1.7291440	-1.9087430
C	-6.2895360	-0.5091340	-2.1860920
H	-6.0567290	-2.6499360	-2.3326380
C	0.0470350	3.0199320	1.2089740

Compound **9a**

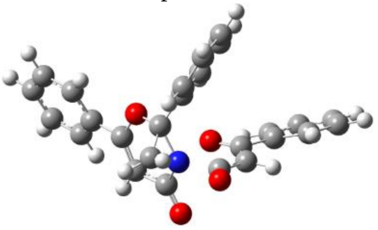

E = -1357.78356042, **H (0K)** = -1357.396435, **H (353K)** = -1357.360805, **G (353K)** = -1357.469550 au.

Imaginary frequency = 0.

C	-0.8894060	1.4586710	0.5114620
O	-1.8521430	0.6405260	-0.0954900
C	0.4165330	1.5393680	-1.6191810
C	-0.1452890	2.2269160	-0.5333650
H	-2.0011970	1.6791170	2.4251970
N	-0.1287280	0.8459170	1.6717290
C	-1.0992090	1.9558000	1.8893290
O	0.0373460	-0.9297910	3.1646190
O	0.8928480	-1.1205550	0.2463740
C	1.0742200	2.2487560	-2.6208720
C	-2.3524920	-0.4657720	0.5221070
C	2.3446530	0.4072400	1.3351170
O	1.5312420	2.2604700	2.6200060
C	0.6082560	4.3262190	-1.4725030
C	-1.7741760	-1.0027910	1.6252370
C	-4.3112840	-0.1454950	-0.9886620
C	-3.9610690	-2.3174580	0.0292240
C	-5.4596460	-0.6274480	-1.6126430
H	0.6773350	5.4084750	-1.4144300
H	-0.4980020	4.1608720	0.3635850
H	-0.6008710	2.8633960	2.2142740
C	1.1711780	3.6415510	-2.5497730
H	1.6849810	4.1911010	-3.3336810
H	1.5152260	1.7133700	-3.4566360
H	-3.9997130	0.8815210	-1.1411630
C	-5.1088260	-2.7932770	-0.5974960
C	-5.8633800	-1.9497850	-1.4176720
H	-5.4085970	-3.8272680	-0.4545960

H	0.3020520	0.4775050	-1.0416010
H	-4.0458930	-2.7208880	-0.9140050
H	-6.2472210	1.6235720	-1.8661850
C	-4.0120320	-0.5876880	-0.5472850
C	1.5711410	-0.5751980	2.6235850
C	-1.2065190	-1.6765670	1.8483410
H	-7.1709730	-0.4795700	-2.8203860
C	1.8171880	-1.7407140	0.3605010
C	2.8814340	-2.4059510	-0.4750030
C	2.4571860	-3.2835990	-1.4851950
C	4.2556720	-2.1690770	-0.3181750
C	3.3829750	-3.9277030	-2.3017010
H	1.3924290	-3.4495790	-1.6100910
C	5.1829890	-2.8035420	-1.1453330
H	4.6112770	-1.4648350	0.4276640
C	4.7500510	-3.6894850	-2.1336020
H	3.0404920	-4.6147330	-3.0708560
H	6.2431790	-2.6016650	-1.0197040
H	5.4736340	-4.1879720	-2.7730180
H	3.2498730	-1.4568340	1.9859090
H	-2.8911730	-2.6598270	0.9227750
N	2.3722630	4.5989240	-1.2542020
O	2.5358890	5.6838830	-0.6951150
O	2.8972710	4.2818090	-2.3215080

C	-0.0517410	3.6206730	-0.4654070
H	0.3503570	0.4575700	-1.6580550
H	-3.3662330	-2.9907800	0.6376520
H	-6.0395970	0.0315330	-2.2522410
C	-3.5536750	-0.9835990	-0.1519830
C	1.4278510	1.2558270	1.9417780
C	-0.5627860	-0.4917400	2.2143710
H	-6.7574090	-2.3245850	-1.9078700
C	2.0538080	-0.7078790	0.4976100
C	3.2074890	-1.4463570	-0.1351080
C	2.9647950	-2.7370370	-0.6303070
C	4.4936270	-0.9027540	-0.2785630
C	3.9819450	-3.4738380	-1.2327510
H	1.9640930	-3.1434650	-0.5291690
C	5.5111310	-1.6358710	-0.8900340
H	4.7023470	0.1056800	0.0641450
C	5.2606270	-2.9252580	-1.3641100
H	3.7790870	-4.4761980	-1.6013110
H	6.4994840	-1.1971080	-0.9995900
H	6.0552220	-3.4969810	-1.8365680
H	3.3728140	0.6627180	1.5564910
H	-2.2013880	-1.8735930	2.1029620

Compound **9c**

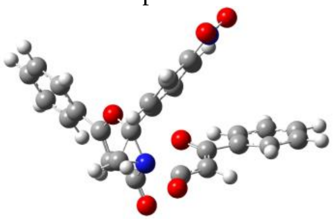

E = -1562.28345839, **H (0K)** = -1561.893934, **H (353K)** = -1561.854873, **G (353K)** = -1561.973158 au.

Imaginary frequency = 0.

C	-0.8748950	0.9205700	1.1812400
O	-1.9043560	0.7749970	0.2440690
C	0.7861560	1.3481330	-0.6406810
C	0.2286370	1.7419940	0.5848320
H	-2.1864760	0.7437500	2.9691880
N	-0.5263660	-0.2946830	1.9999680
C	-1.1657820	0.8929200	2.6326150
O	-1.1218370	-2.4589050	2.6061360
O	0.1394570	-1.7515700	-0.0848280
C	1.7756770	2.1233830	-1.2344660
C	-2.7960310	-0.2555180	0.3135750
C	1.7460690	-1.3709540	1.6169850
O	1.2862240	0.0228290	3.5158030
C	1.6472100	3.7029070	0.6222030
C	-2.5899080	-1.3355400	1.1079180
C	-4.2935250	1.2505070	-0.9939080
C	-4.7471770	-1.1314260	-0.9817310
C	-5.4054110	1.4543970	-1.8075610
H	1.9927730	4.6178250	1.0860150
H	0.2182030	3.2343070	2.1488880
H	-0.4915190	1.3869540	3.3253860
C	2.1881660	3.2901220	-0.5917650
H	2.2282780	1.8369370	-2.1752670
H	-3.6859020	2.0927170	-0.6830570

TS **9a** → **3a**

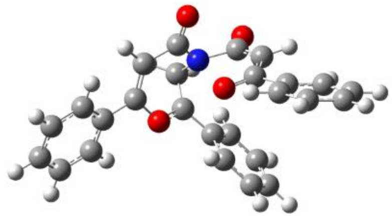

E = -1357.77528907, **H (0K)** = -1357.388757, **H (353K)** = -1357.353494, **G (353K)** = -1357.462696 au.

Imaginary frequency = 1.

C	-0.9739940	1.4826680	0.3991000
O	-1.8166920	0.6201710	-0.1909150
C	0.2837290	1.5291790	-1.7332200
C	-0.0811970	2.1643240	-0.5294120
H	-2.0240730	1.5733920	2.3221150
N	-0.1220410	0.6346110	1.8880570
C	-1.0680870	1.7574890	1.8365910
O	-0.0835810	-1.4029390	2.9767020
O	0.8995830	-1.1752940	0.1309320
C	1.1047320	2.1876400	-2.6402380
C	-2.4674190	-0.4065040	0.4760330
C	2.2761110	0.2049260	1.5109000
O	1.4729560	1.9718020	2.9076860
C	1.2161330	4.1090500	-1.1644990
C	-1.9391560	-0.9836660	1.5762360
C	-4.3687810	0.1099340	-1.0551660
C	-4.2513400	-2.0841840	-0.0337980
C	-5.5554180	-0.2522220	-1.6890610
H	1.5802030	5.1079620	-0.9457280
H	0.1315100	3.9558240	0.6779370
H	-0.5800540	2.6567610	2.2033900
C	1.5674590	3.4776340	-2.3601690
H	2.2105060	3.9878450	-3.0716630
H	1.3952460	1.6922800	-3.5614650

C	-5.8561940	-0.9211220	-1.7953260
C	-6.1908960	0.3716550	-2.2080700
H	-6.4538520	-1.7687430	-2.1172330
C	0.6574430	2.9179410	1.2090090
H	0.4558600	0.4263030	-1.1058550
H	-4.4790680	-2.1429200	-0.6949000
H	-5.6582450	2.4605530	-2.1288960
C	-3.9575250	-0.0451890	-0.5634070
C	1.0259440	-0.5586840	2.4814710
C	-1.4058660	-1.5186910	1.9073260
H	-7.0559270	0.5322050	-2.8451470
C	1.2972460	-1.9169270	0.3790050
C	2.2630040	-2.7403880	-0.4342660
C	1.7312950	-3.5628020	-1.4400510
C	3.6552150	-2.7101200	-0.2568470
C	2.5631930	-4.3487920	-2.2337670
H	0.6555430	-3.5698230	-1.5793710
C	4.4902350	-3.4896750	-1.0575800
H	4.0991840	-2.0605900	0.4905730
C	3.9474250	-4.3151270	-2.0445160
H	2.1337750	-4.9877300	-3.0011100
H	5.5664660	-3.4487410	-0.9123390
H	4.5992600	-4.9249190	-2.6646060
H	2.7438410	-1.5895670	1.9748910
H	-3.3201500	-2.1315170	1.1544250
N	3.2315440	4.1186410	-1.2209540
O	3.5804490	5.1435370	-0.6355310
O	3.6929970	3.7395930	-2.2966620

H	-3.9548100	1.1003630	-1.2092280
C	-5.4369480	-2.4398850	-0.6694470
C	-6.0946510	-1.5253440	-1.4967220
H	-5.8414360	-3.4378560	-0.5287850
C	0.3999250	3.4561740	-0.2460900
H	-0.0500690	0.5161570	-1.9221480
H	-3.7337590	-2.8155310	0.5781730
H	-6.0583270	0.4621330	-2.3343270
C	-3.7073680	-0.7993510	-0.2114510
C	1.3289540	0.9917210	2.1720790
C	-0.6471390	-0.6700420	2.1910420
H	-7.0178510	-1.8076060	-1.9946080
C	2.0309630	-0.7836560	0.5090370
C	3.2347370	-1.4135090	-0.1605470
C	3.0520670	-2.6515560	-0.7961030
C	4.5060150	-0.8206630	-0.2062750
C	4.1105030	-3.2912220	-1.4376580
H	2.0625380	-3.0958450	-0.7706260
C	5.5653100	-1.4537570	-0.8584820
H	4.6700310	0.1515150	0.2475350
C	5.3735110	-2.6937650	-1.4711950
H	3.9521840	-4.2561620	-1.9131510
H	6.5410100	-0.9752690	-0.8901310
H	6.2005420	-3.1886860	-1.9740520
H	3.2973920	0.4363800	1.7855850
H	-2.4565400	-1.8243440	2.0206350

TS **9c** → **3c**

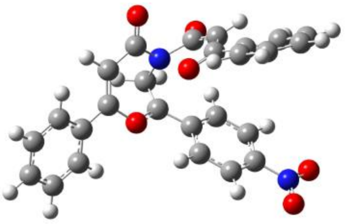

E = -1562.27203434, **H (0K)** = -1561,883014,
H (353K) = -1561,844445, **G (353K)** = -1561,962005 au.
Imaginary frequency = 1.

C	-1.0637020	0.8488370	1.1265460
O	-2.0028680	0.6776720	0.1883590
C	0.1721640	2.0257270	-0.6712190
C	0.0989110	1.6110730	0.6746450
H	-2.3398790	0.2687530	2.8012480
N	-0.6551920	-0.8292430	2.0018720
C	-1.3011520	0.3908720	2.4996270
O	-1.2537490	-3.0543430	1.8484110
O	0.2622240	-1.7028750	-0.3944670
C	1.2420650	2.7902560	-1.1122720
C	-2.9593350	-0.3266710	0.2285420
C	1.6244450	-1.6503370	1.5617070
O	0.9638510	-0.6911280	3.6505200
C	2.2183120	2.7040580	1.1231500
C	-2.7304850	-1.4959020	0.8645930
C	-4.4830810	1.3777250	-0.7700810
C	-4.9839400	-0.9725630	-1.0875510
C	-5.6277010	1.7154080	-1.4888620
H	3.0278030	2.9674780	1.7916240
H	1.1335310	1.5984090	2.5902040

Compound **3a**

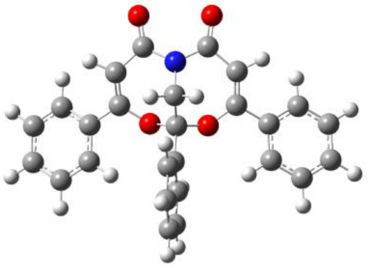

E = -1357.82759348, **H (0K)** = -1357.436984,
H (353K) = -1357.402503, **G (353K)** = -1357.508685 au.
Imaginary frequency = 0.

C	0.0119940	-0.0590990	0.4906770
O	1.1602410	0.0234190	-0.3691970
C	0.0014930	-2.5375400	0.0515560
C	-0.0515140	-1.4988050	0.9961620
H	0.9135860	0.8724110	2.1955910
N	0.0170690	2.3494860	1.0274290
C	0.0270740	1.0009980	1.5710700
O	1.3970970	4.1620400	0.5957050
O	-1.0868920	0.1270870	-0.4282220
C	-0.0565610	-3.8678190	0.4592340
C	2.2799720	0.7814160	-0.1618750
C	-2.3274320	2.0250850	0.4595380
O	-1.4123770	4.1653550	0.7999370
C	-0.2197670	-3.1577910	2.7597270
C	2.3438780	2.0211990	0.3878130
C	3.5725570	-1.3104830	-0.6467960
C	4.5669180	0.8221790	-1.2077110
C	4.7188480	-1.9605550	-1.1000120

H	-0.6995540	0.8378570	3.2866400
C	2.2473270	3.1211520	-0.2041610
H	1.3117040	3.1229780	-2.1398390
H	-3.8489660	2.1602130	-0.3674780
C	-6.1259060	-0.6285480	-1.8043180
C	-6.4532240	0.7152210	-2.0054890
H	-6.7545190	-1.4113980	-2.2179480
C	1.1388220	1.9466430	1.5642470
H	-0.6109560	1.7472210	-1.3646690
H	-4.7230130	-2.0189750	-0.9684440
H	-5.8744930	2.7615710	-1.6439420
C	-4.1529460	0.0284370	-0.5531950
C	0.7559030	-1.0887290	2.4979830
C	-1.5077070	-1.9038940	1.5624140
H	-7.3432250	0.9801780	-2.5688370
C	1.3653680	-1.8816700	0.1698140
C	2.5150720	-2.3657400	-0.6868460
C	2.2018150	-2.9420580	-1.9279220
C	3.8666740	-2.2456360	-0.3275340
C	3.2049410	-3.4037280	-2.7768880
H	1.1557340	-3.0173730	-2.2053070
C	4.8738770	-2.6979950	-1.1810750
H	4.1443480	-1.7766110	0.6108760
C	4.5469900	-3.2833210	-2.4057990
H	2.9422390	-3.8568820	-3.7295430
H	5.9153300	-2.5891360	-0.8895120
H	5.3322230	-3.6392430	-3.0677880
H	2.6034080	-1.8783850	1.9632540
H	-3.4858060	-2.2692210	0.8041170
N	3.3839460	3.9376520	-0.6689880
O	4.2612420	4.2128010	0.1477860
O	3.3832730	4.2981230	-1.8450060

H	-0.3049830	-3.3905890	3.8173050
H	-0.2050370	-1.0460370	3.1108630
H	-0.8481400	0.8790380	2.2106740
C	-0.1671810	-4.1826550	1.8175100
H	-0.2115070	-5.2203780	2.1357410
H	-0.0146730	-4.6602010	-0.2828960
H	2.7421190	-1.8869340	-0.2549960
C	5.7093580	0.1682720	-1.6638240
C	5.7917560	-1.2249560	-1.6077800
H	6.5306750	0.7478000	-2.0757670
C	-0.1632230	-1.8210120	2.3526760
H	0.0901870	-2.2932920	-1.0021560
H	4.5001620	1.9024830	-1.2881370
H	4.7731290	-3.0448900	-1.0570850
C	3.4881500	0.0917770	-0.6797240
C	-1.2387700	2.9600430	0.7876800
C	1.2456310	2.9563050	0.6878630
H	6.6820630	-1.7338930	-1.9666630
C	-2.2369500	0.8222980	-0.1664550
C	-3.4267480	0.1375330	-0.7285410
C	-3.4619130	-1.2642800	-0.8185920
C	-4.5367950	0.8720440	-1.1821250
C	-4.5899610	-1.9129330	-1.3172890
H	-2.6073500	-1.8422900	-0.4862800
C	-5.6608100	0.2201760	-1.6837880
H	-4.5087460	1.9568600	-1.1700520
C	-5.6937520	-1.1748260	-1.7493920
H	-4.6054470	-2.9980840	-1.3697740
H	-6.5063580	0.8036600	-2.0369290
H	-6.5696470	-1.6820070	-2.1444200
H	-3.3155090	2.4516800	0.5792560
H	3.3239370	2.4756210	0.4623140

Compound **3c**

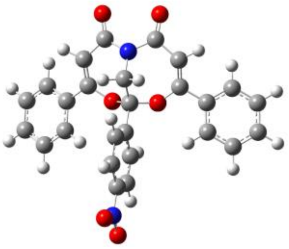

E = -1562.32797733, **H (0K)** = -1561.934928, **H (353K)** = -1561.897050, **G (353K)** = -1562.011918 au.

Imaginary frequency = 0.

C	-0.0593000	-0.4341100	0.3694600
O	-1.2238640	-0.5954300	-0.4527590
C	0.1414530	1.8930800	-0.5686920
C	0.1113700	1.0731390	0.5733910
H	-0.9868990	-0.9267590	2.2376480
N	-0.2487450	-2.6741630	1.3766130
C	-0.1306810	-1.2506530	1.6421310
O	-1.7981380	-4.4004370	1.3796230
O	1.0121870	-0.8607360	-0.4904670
C	0.2976860	3.2682410	-0.4523160
C	-2.4029410	-1.1935210	-0.0883190
C	2.0994350	-2.6679450	0.7294160
O	1.0074510	-4.6248590	1.4401130
C	0.3965140	3.0377310	1.9702770
C	-2.5588940	-2.2797010	0.7108490

TS **8a → 10a**

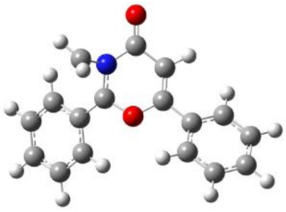

E = -860.73288218, **H (0K)** = -860.476842, **H (353K)** = -860.454157, **G (353K)** = -860.533485 au.

Imaginary frequency = 1.

C	-0.2440490	2.6230770	-0.2958280
N	-1.1883520	1.8227300	0.4077870
C	-1.9377800	2.0534510	1.5507040
C	-2.1840370	-0.4863220	0.1232320
C	-1.9195970	-1.8679690	0.1499110
C	-2.9011240	-2.7871100	-0.2252380
C	-4.1556980	-2.3520340	-0.6464380
C	-4.4236290	-0.9770540	-0.6844230
C	-3.4607130	-0.0565900	-0.2956830
O	0.1446960	-0.0898270	0.5266060
C	1.1568010	0.6087920	-0.0706800
C	0.9543630	1.8504640	-0.5977180
C	-1.1248750	0.4658830	0.4686960
O	-0.4111850	3.8142160	-0.4864470
H	-1.5224460	1.7501030	2.5026380
H	-0.9415390	-2.2215330	0.4554920
H	-2.6741530	-3.8493810	-0.1908660

C	-3.5275460	0.8522240	-1.0036940
C	-4.6970260	-1.2625880	-1.1104500
C	-4.6231230	1.4846610	-1.5877920
H	0.4963580	3.5016420	2.9434550
H	0.2191290	1.0567650	2.7404590
H	0.7694270	-1.0787020	2.2349880
C	0.4236770	3.8218050	0.8226810
H	0.3226010	3.9104200	-1.3237710
H	-2.6494880	1.4295280	-0.7372700
C	-5.7887100	-0.6276190	-1.6982100
C	-5.7578920	0.7485600	-1.9351320
H	-6.6589580	-1.2116140	-1.9837400
C	0.2395380	1.6576800	1.8384220
H	0.0405170	1.4437310	-1.5504610
H	-4.7176780	-2.3376380	-0.9635470
H	-4.5896610	2.5546730	-1.7729190
C	-3.5563490	-0.5281060	-0.7421800
C	0.9399430	-3.4283040	1.2273860
C	-1.5389460	-3.2247290	1.1974620
H	-6.6085950	1.2420790	-2.3966300
C	2.1045900	-1.5990170	-0.1076500
C	3.3379290	-1.1388460	-0.7897390
C	3.5005780	0.2132980	-1.1356870
C	4.3678970	-2.0441480	-1.1020970
C	4.6740980	0.6521730	-1.7455560
H	2.7102820	0.9214470	-0.9156090
C	5.5379960	-1.6024780	-1.7148550
H	4.2407620	-3.1012000	-0.8920060
C	5.6978620	-0.2521650	-2.0348490
H	4.7880040	1.7031190	-1.9962730
H	6.3200110	-2.3170540	-1.9551670
H	6.6095650	0.0905390	-2.5160690
H	3.0484180	-3.1524750	0.9228350
H	-3.5726930	-2.6192090	0.8837700
N	0.5898500	5.2768820	0.9558170
O	0.6959120	5.7414770	2.0908010
O	0.6134790	5.9474500	-0.0763410

H	-4.9180790	-3.0679050	-0.9399060
H	-5.3955660	-0.6212490	-1.0157710
H	-3.6923330	1.0033990	-0.3110930
C	2.4340950	-0.1253440	-0.0536420
C	2.6319610	-1.1828980	0.8511650
C	3.4742350	0.2185090	-0.9349920
C	3.8459080	-1.8660860	0.8825400
H	1.8375330	-1.4575820	1.5358430
C	4.6831480	-0.4708090	-0.9023660
H	3.3323250	1.0096320	-1.6640080
C	4.8753710	-1.5132840	0.0080630
H	3.9868420	-2.6758770	1.5927530
H	5.4742660	-0.1976940	-1.5946800
H	5.8197260	-2.0496740	0.0313140
H	1.7772400	2.3912350	-1.0463850
H	-2.8597220	2.6242430	1.5050700

TS **8b → 10b**

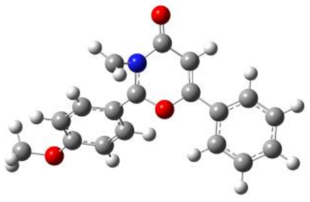

E = -975.25677262, **H (0K)** = -974.967636, **H (353K)** = -974.941699, **G (353K)** = -975.028169 au.
Imaginary frequency = 1.

C	1.0445930	2.7176920	-0.6299020
N	-0.0017000	2.3045550	0.2283910
C	-0.6494880	2.8798540	1.3113290
C	-1.5333430	0.2791950	0.3379900
C	-1.4966100	-0.9886720	-0.2876560
C	-2.6617610	-1.6783240	-0.5814420
C	-3.9136340	-1.1262450	-0.2654920
C	-3.9704500	0.1193160	0.3727650
C	-2.7904640	0.7895490	0.6911720
O	0.8331320	0.1695190	0.6556300
C	1.9330530	0.4837830	-0.0964250
C	1.9864120	1.6235800	-0.8428210
C	-0.2739680	1.0101440	0.5512430

TS **8c → 10c**

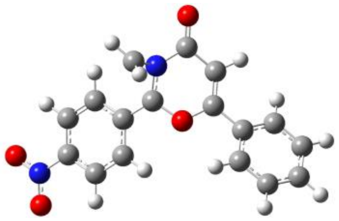

E = -1065.23878013, **H (0K)** = -1064.980120, **H (353K)** = -1064.954143, **G (353K)** = -1065.041663 au.
Imaginary frequency = 1.

C	1.4352330	2.7971250	-0.4006840
N	0.3203640	2.3553640	0.3767120
C	-0.2389690	2.8264160	1.5540570
C	-1.3349110	0.4618620	0.3318690
C	-1.5275210	-0.9210190	0.5346190
C	-2.7665310	-1.5080520	0.3107380
C	-3.8246430	-0.7175670	-0.1332020
C	-3.6591930	0.6559340	-0.3570180
C	-2.4291660	1.2376270	-0.1187640
O	1.0230650	0.1317540	0.5699180
C	2.1678010	0.4601310	-0.1091810

O	1.1605890	3.8654610	-1.0258810
H	-0.2087370	2.7415980	2.2908310
H	-0.5432120	-1.4225300	-0.5678520
H	-2.6285250	-2.6465380	-1.0713650
H	-4.9191220	0.5552580	0.6636160
H	-2.8362500	1.7195040	1.2473210
C	2.9873320	-0.5425310	-0.0048990
C	2.6938250	-1.8187410	0.5077450
C	4.3003390	-0.2680290	-0.4278980
C	3.6840580	-2.7966000	0.5778000
H	1.6889580	-2.0396840	0.8486640
C	5.2849610	-1.2492240	-0.3585450
H	4.5592100	0.7201210	-0.7936840
C	4.9807410	-2.5179840	0.1422630
H	3.4403500	-3.7787880	0.9727180
H	6.2946240	-1.0195960	-0.6867070
H	5.7516970	-3.2813360	0.1979320
H	2.8452510	1.8303270	-1.4672760
H	-1.3814540	3.6672510	1.1542510
O	-4.9972230	-1.8812770	-0.5952230
C	-6.2921820	-1.3617140	-0.3152420
H	-6.4630820	-0.4110180	-0.8354310
H	-6.9983840	-2.1080630	-0.6821150
H	-6.4418850	-1.2183040	0.7622210

C	2.3244160	1.6814080	-0.6952020
C	-0.0133330	1.0447280	0.5277360
O	1.6271830	3.9715220	-0.6493420
H	0.1432440	2.4458250	2.4926630
H	-0.6990120	-1.5367630	0.8631850
H	-2.9199740	-2.5679080	0.4717060
H	-4.4953470	1.2429610	-0.7156820
H	-2.3026960	2.3018050	-0.2842420
C	3.1547240	-0.6319440	-0.1057270
C	3.0754430	-1.6564100	0.8537670
C	4.1908910	-0.6666530	-1.0557650
C	4.0205590	-2.6797120	0.8707480
H	2.2812830	-1.6406380	1.5915990
C	5.1288950	-1.6947630	-1.0369380
H	4.2509030	0.0955120	-1.8258350
C	5.0497140	-2.7028250	-0.0723940
H	3.9520490	-3.4602620	1.6229070
H	5.9189680	-1.7127450	-1.7818700
H	5.7831010	-3.5039810	-0.0604200
H	3.2474860	1.9228930	-1.2053380
H	-0.9624010	3.6356710	1.5405150
N	-5.1243760	-1.3287020	-0.3725290
O	-6.0449860	-0.6011290	-0.7586560
O	-5.2415070	-2.5429870	-0.1782200

Compound **10a**

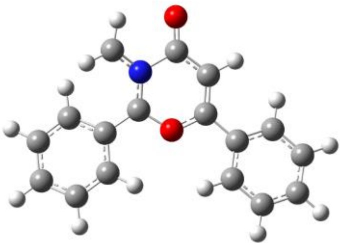

E = -860.76741252, **H (0K)** = -860.509791,
H (353K) = -860.486716, **G (353K)** = -860.566591 au.
Imaginary frequency = 0.

C	-1.1189700	0.4915540	0.1300050
N	-1.2424040	1.8457080	0.3128610
C	-2.3368180	2.5093300	0.6833930
C	-2.1914510	-0.4800850	0.0271420
C	-1.9144030	-1.8496640	0.2602120
C	-2.8967440	-2.8197620	0.0990610
C	-4.1859330	-2.4698630	-0.3113640
C	-4.4717810	-1.1263090	-0.5632140
C	-3.5024590	-0.1434040	-0.3871130
O	0.1330050	-0.0636460	0.1280590
C	1.2554140	0.6477270	-0.0266850
C	1.1684860	2.0094310	-0.1663090
C	-0.0557570	2.7099600	-0.0315360
O	-0.2411720	3.9121560	-0.1034260
H	-3.1892860	1.9685760	1.0562340
H	-0.9189480	-2.1470950	0.5679050
H	-2.6518260	-3.8599420	0.2978760
H	-4.9530610	-3.2294500	-0.4302930
H	-5.4639350	-0.8337410	-0.8969050
H	-3.7575900	0.8864180	-0.6067150
C	2.4812740	-0.1590570	-0.0284170
C	2.4729310	-1.4788070	0.4588530
C	3.6895340	0.3743900	-0.5139000
C	3.6398760	-2.2383010	0.4632620

Compound **10b**

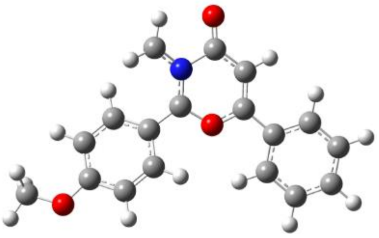

E = -975.29166489, **H (0K)** = -975.001303,
H (353K) = -974.974734, **G (353K)** = -975.062359 au.
Imaginary frequency = 0.

C	0.2593580	0.9749520	-0.1083030
N	0.0266950	2.3221770	-0.1999870
C	0.9175310	3.2748340	-0.4866640
C	1.5489340	0.3081740	-0.1411490
C	1.6307100	-1.0340580	-0.5913460
C	2.8231040	-1.7356970	-0.5598570
C	3.9945740	-1.1332970	-0.0733490
C	3.9381530	0.1886650	0.3838820
C	2.7369890	0.8942810	0.3416720
O	-0.7982720	0.1098830	-0.1102660
C	-2.0698620	0.4871540	0.0609900
C	-2.3481000	1.8186420	0.2401960
C	-1.3537980	2.8227780	0.1405550
O	-1.4971370	4.0278370	0.2606520
H	1.8982070	3.0022750	-0.8352310
H	0.7417560	-1.5219370	-0.9750660
H	2.8749630	-2.7595350	-0.9174940
H	4.8205950	0.6824430	0.7743090
H	2.7266270	1.9075700	0.7262590
C	-3.0256090	-0.6250850	0.0629970
C	-2.5853260	-1.9487820	0.2505360
C	-4.4004490	-0.3879220	-0.1190960
C	-3.4973560	-3.0006600	0.2605210
H	-1.5294910	-2.1447040	0.4019570

H	1.5524760	-1.9010940	0.8461100
C	4.8531660	-0.3896860	-0.5063470
H	3.7179960	1.3808450	-0.9183780
C	4.8350400	-1.6982180	-0.0173020
H	3.6160930	-3.2541470	0.8475260
H	5.7756140	0.0368490	-0.8899560
H	5.7444370	-2.2922930	-0.0129020
H	2.0520450	2.6179380	-0.2991330
H	-2.2902750	3.5836930	0.6330950

C	-5.3075430	-1.4440970	-0.1049780
H	-4.7594460	0.6213800	-0.2931200
C	-4.8613870	-2.7543980	0.0847730
H	-3.1418860	-4.0161400	0.4113800
H	-6.3651350	-1.2435660	-0.2512340
H	-5.5709230	-3.5769160	0.0937710
H	-3.3536700	2.1597310	0.4432610
H	0.5730050	4.2917010	-0.4152760
O	5.1171320	-1.9057760	-0.0955830
C	6.3285230	-1.3469920	0.3978990
H	6.2412120	-1.0711890	1.4564560
H	7.0834880	-2.1271230	0.2885610
H	6.6293730	-0.4657360	-0.1827090

Compound **10c**

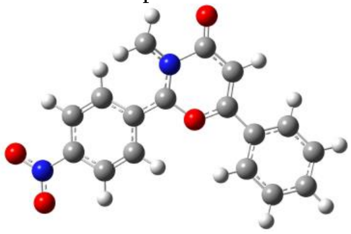

E = -1065.27553352, **H (0K)** = -1065.015164,
H (353K) = -1064.988845, **G (353K)** = -1065.076842 au.
Imaginary frequency = 0.

C	-0.0163780	1.0678270	0.2389740
N	0.3292330	2.3955460	0.3529930
C	-0.4411800	3.3849380	0.7838530
C	-1.3337750	0.5068400	0.1666140
C	-1.4904800	-0.9063750	0.2694500
C	-2.7279790	-1.5069590	0.1469870
C	-3.8580570	-0.7199310	-0.1042750
C	-3.7408610	0.6662800	-0.2433730
C	-2.5058090	1.2710570	-0.0932020
O	0.9957640	0.1375710	0.2361490
C	2.2663060	0.4429540	-0.0535170
C	2.6079330	1.7427970	-0.3192380
C	1.6925880	2.8145720	-0.1601960
O	1.8856550	4.0031510	-0.3209640
H	-1.3664520	3.1663910	1.2894420
H	-0.6214370	-1.5275640	0.4429160
H	-2.8370760	-2.5802520	0.2426690
H	-4.6221830	1.2562390	-0.4626330
H	-2.4460200	2.3441210	-0.2191540
C	3.1719940	-0.7121850	-0.0417110
C	2.8156810	-1.8910860	0.6377980
C	4.4102620	-0.6563370	-0.7065600
C	3.6799020	-2.9826840	0.6539700
H	1.8703780	-1.9411320	1.1669030
C	5.2689880	-1.7517310	-0.6876290
H	4.6931770	0.2327630	-1.2607260
C	4.9086240	-2.9175670	-0.0071390
H	3.3946880	-3.8844210	1.1879780
H	6.2186030	-1.6971480	-1.2118010
H	5.5808230	-3.7706680	0.0058530
H	3.6258530	2.0158260	-0.5600130
H	-0.0706870	4.3869690	0.6402450
N	-5.1577270	-1.3460920	-0.2236660
O	-6.1405790	-0.6201650	-0.4230180
O	-5.2241850	-2.5782780	-0.1236440

Compound **11a**

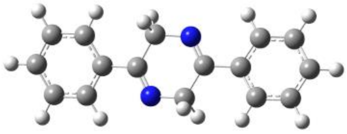

E = -727.62293181, **H (0K)** = -727.360948,
H (353K) = -727.340258, **G (353K)** = -727.415859 au.
Imaginary frequency = 0.

C	-1.3883350	-0.0602420	0.1583240
N	-0.8028130	-1.1977950	0.1511980
C	0.6419520	-1.2547340	0.2941260
C	1.3883560	0.0603060	0.1586020
N	0.8028310	1.1978550	0.1511910
C	-0.6419560	1.2548100	0.2938720
H	1.0190870	-1.9738260	-0.4450360
H	0.8673580	-1.7059080	1.2730810
H	-0.8675330	1.7060740	1.2727480
H	-1.0189570	1.9738300	-0.4454260
C	-2.8733530	-0.0221200	0.0202930
C	-3.5774830	-1.2027600	-0.2756950
C	-3.6007960	1.1672820	0.1880880
C	-4.9624720	-1.1922340	-0.4036500
H	-3.0133790	-2.1203750	-0.4023800
C	-4.9915170	1.1762590	0.0635660
H	-3.0927230	2.0965310	0.4251710
C	-5.6766450	-0.0011950	-0.2346230
H	-5.4892820	-2.1137940	-0.6369300
H	-5.5368470	2.1061120	0.2002840
H	-6.7586740	0.0065740	-0.3349100
C	2.8733610	0.0221400	0.0204410
C	3.5775110	1.2027620	-0.2755730
C	3.6007700	-1.1673020	0.1880950
C	4.9624880	1.1921880	-0.4036490
H	3.0134320	2.1204060	-0.4021630
C	4.9914810	-1.1763270	0.0634570
H	3.0926770	-2.0965440	0.4251640
C	5.6766300	0.0011130	-0.2347320
H	5.4893140	2.1137370	-0.6369360
H	5.5367840	-2.1062110	0.2000790
H	6.7586500	-0.0066950	-0.3351170

TS **11a+6a → 12a’**

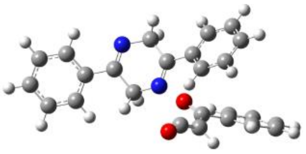

E = -1224.61141850, **H (0K)** = -1224.223399,
H (353K) = -1224.189202, **G (353K)** = -1224.297735 au.
Imaginary frequency = 1.

C	-2.8965120	0.0955550	-0.4458190
N	-0.6340740	0.4647340	0.5555480
C	-1.7784320	-0.4273720	0.4368030
C	-4.2692580	-0.4533460	-0.2614370
C	-5.3602590	0.1706240	-0.8918980
C	-6.6487580	-0.3318740	-0.7430610
C	-6.8745640	-1.4732320	0.0336710
C	-5.8000080	-2.1060120	0.6581170
C	-4.5072800	-1.5987840	0.5149840
O	0.9529260	-1.5360160	-0.5065430
C	1.8676980	-1.3918360	0.3104260
C	1.7051850	-0.8875890	1.6669980
C	0.6578370	-0.2874830	2.2700980
O	0.0374670	0.1057510	3.1867230
H	-1.3967620	-1.3806020	0.0461970
H	-5.1732660	1.0523550	-1.4952590
H	-7.4814260	0.1665870	-1.2322620
H	-7.8814890	-1.8651520	0.1492910
H	-5.9638920	-2.9964850	1.2587270
H	-3.6863850	-2.1147350	1.0029200
C	3.2633260	-1.8109690	-0.0761040
C	3.4044020	-2.7057400	-1.1479100
C	4.4163760	-1.3251180	0.5581450
C	4.6665120	-3.1230190	-1.5624520
H	2.5068230	-3.0634570	-1.6415520
C	5.6819800	-1.7342550	0.1362210
H	4.3323710	-0.6017970	1.3635400
C	5.8097820	-2.6386720	-0.9197890
H	4.7614960	-3.8247190	-2.3868470
H	6.5676060	-1.3426050	0.6292440
H	6.7956480	-2.9612290	-1.2440660
H	2.4966260	-1.0801110	2.3872580
H	-2.1470890	-0.6213170	1.4501410
C	-1.3569110	1.4992220	-1.5328080
C	-0.4312420	1.3586670	-0.3359240
N	-2.7044450	0.9879220	-1.3430180
C	0.7089780	2.3028820	-0.2146330
C	1.1316610	2.7550180	1.0462020
C	2.1884430	3.6562990	1.1555030
C	2.8429810	4.1148580	0.0093110
C	2.4288630	3.6736900	-1.2488340
C	1.3626020	2.7820520	-1.3615860
H	-0.9050070	0.9711110	-2.3876620
H	-1.4248640	2.5513570	-1.8306170
H	0.6110890	2.4129040	1.9342430
H	2.4976610	4.0064070	2.1365580
H	3.6678060	4.8168300	0.0962660
H	2.9343640	4.0241390	-2.1443050
H	1.0547890	2.4426440	-2.3462920

Compound **12a’**

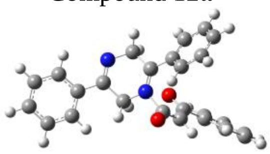

E = -1224.62433645, **H (0K)** = -1224.233457,
H (353K) = -1224.199524, **G (353K)** = -1224.306875 au.
Imaginary frequency = 0.

C	-1.8746194	-0.1325492	-0.0036212
N	0.3929207	0.1657127	0.9446517
C	-0.7208114	-0.7685453	0.7461891
C	-3.2423613	-0.6771331	0.1962720
C	-4.3447337	0.0072182	-0.3466653
C	-5.6340563	-0.4875967	-0.1850228
C	-5.8483702	-1.6794871	0.5153933
C	-4.7624987	-2.3711495	1.0514767
C	-3.4681172	-1.8728526	0.8969270
O	1.9426263	-1.3216184	-0.6633295
C	2.6974296	-1.4829355	0.3238645
C	2.4162993	-1.0435452	1.6614909
C	1.3371339	-0.2764776	2.0941939
O	0.9659386	0.0788052	3.2103479
H	-0.3126159	-1.6172684	0.1816332
H	-4.1675439	0.9282366	-0.8910593
H	-6.4759905	0.0560211	-0.6046904
H	-6.8563624	-2.0651039	0.6407552
H	-4.9185095	-3.3001241	1.5923045
H	-2.6406319	-2.4361616	1.3162834
C	3.9934061	-2.2290506	0.0947572
C	4.1010085	-3.0073279	-1.0680909
C	5.0964632	-2.1597585	0.9597054
C	5.2669759	-3.7156400	-1.3496048
H	3.2501526	-3.0386233	-1.7405556
C	6.2694112	-2.8600757	0.6744483
H	5.0544530	-1.5384040	1.8486395
C	6.3566483	-3.6449873	-0.4771168
H	5.3290402	-4.3215505	-2.2501050
H	7.1172413	-2.7887786	1.3509008
H	7.2690894	-4.1937604	-0.6958529
H	3.0700705	-1.3543191	2.4673667
H	-1.0173355	-1.1090689	1.7395674
C	-0.3437875	1.3302030	-1.0191560
C	0.6030946	1.1570880	0.1465790
N	-1.6961848	0.8429428	-0.8117711
C	1.7086882	2.1156518	0.3190836
C	2.0625067	2.6165432	1.5850388
C	3.0747933	3.5668786	1.6969422
C	3.7505466	4.0160152	0.5604481
C	3.4042168	3.5213528	-0.6994467
C	2.3781662	2.5890350	-0.8243234
H	0.1011995	0.7899581	-1.8706549
H	-0.3882115	2.3853380	-1.3004732
H	1.5390214	2.2694727	2.4686070
H	3.3356228	3.9558119	2.6767151
H	4.5436596	4.7523573	0.6549738
H	3.9308192	3.8632762	-1.5853687
H	2.1204889	2.2051414	-1.8064194

TS **12a’ → 12a**

Compound **12a**

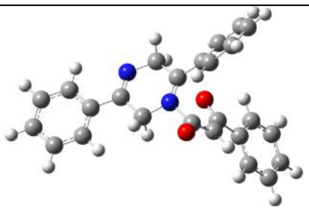

E = -1224.62375805, **H (0K)** = -1224.233051,
H (353K) = -1224.200237, **G (353K)** = -1224.303628 au.
Imaginary frequency = 1.

C	-1.3800244	0.2576602	-0.2599079
N	0.7805406	0.7785932	0.8694521
C	-0.4389634	-0.0325398	0.8891141
C	-2.7505884	-0.3191768	-0.1864199
C	-3.6790704	-0.0275768	-1.2015339
C	-4.9633534	-0.5590518	-1.1608119
C	-5.3463714	-1.3964048	-0.1075839
C	-4.4335964	-1.6963658	0.9030611
C	-3.1451574	-1.1608858	0.8661411
O	2.5916796	-0.2608038	-0.8566389
C	3.1504326	-0.7156008	0.1838791
C	2.8409996	-0.3524948	1.5271471
C	1.7422056	0.3910422	1.9872941
O	1.3808506	0.6767772	3.1240991
H	-0.1431824	-1.0904258	0.8651231
H	-3.3707374	0.6200252	-2.0147419
H	-5.6697254	-0.3214698	-1.9515169
H	-6.3500354	-1.8114748	-0.0772709
H	-4.7204344	-2.3475528	1.7238341
H	-2.4547534	-1.4144878	1.6641511
C	4.2554016	-1.7168078	-0.0343629
C	4.8951596	-1.7361478	-1.2837879
C	4.6531266	-2.6469498	0.9388941
C	5.9203146	-2.6419638	-1.5465529
H	4.5729316	-1.0267028	-2.0383529
C	5.6737176	-3.5602978	0.6737981
H	4.1489046	-2.6773758	1.8995421
C	6.3143006	-3.5575718	-0.5671289
H	6.4124736	-2.6359518	-2.5157389
H	5.9638716	-4.2791008	1.4355781
H	7.1113246	-4.2676818	-0.7715419
H	3.4856096	-0.7133678	2.3186471
H	-0.9142714	0.1476932	1.8573831
C	0.2810626	1.5027942	-1.3957599
C	1.1761886	1.4473432	-0.1771309
N	-1.0459244	0.9410842	-1.2866449
C	2.2794046	2.4311382	-0.0943509
C	2.4530136	3.1982222	1.0698531
C	3.4221226	4.1998902	1.1087811
C	4.2229106	4.4460612	-0.0070789
C	4.0502956	3.6890992	-1.1698859
C	3.0791726	2.6936792	-1.2195999
H	0.8141476	0.9947712	-2.2102199
H	0.1828396	2.5500932	-1.7053849
H	1.8279136	3.0151152	1.9372591
H	3.5480906	4.7873642	2.0134711
H	4.9790896	5.2254012	0.0267001
H	4.6764786	3.8728282	-2.0381039
H	2.9662096	2.0892372	-2.1125349

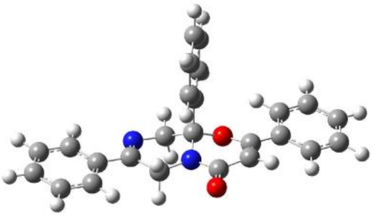

E = -1224.65380902, **H (0K)** = -1224.260581,
H (353K) = -1224.228071, **G (353K)** = -1224.329740 au.
Imaginary frequency = 0.

C	-2.5323700	-0.4922900	-0.6314840
N	-0.3177030	-1.0559180	0.3546820
C	-1.7587360	-1.2825610	0.4050820
C	-4.0189370	-0.4799400	-0.5060610
C	-4.7760190	0.3495660	-1.3521380
C	-6.1638510	0.3777120	-1.2640870
C	-6.8271110	-0.4257980	-0.3309980
C	-6.0883540	-1.2560120	0.5116480
C	-4.6951560	-1.2819990	0.4275930
O	1.5808090	-0.3354120	-0.8645430
C	2.4059200	-0.8404520	0.0986430
C	1.8915410	-1.3968770	1.2203330
C	0.4548770	-1.6452590	1.3429520
O	-0.0487670	-2.3669480	2.2054910
H	-1.9533060	-2.3537450	0.2608510
H	-4.2518000	0.9673000	-2.0728630
H	-6.7319580	1.0285040	-1.9234100
H	-7.9113670	-0.4034950	-0.2626720
H	-6.5932720	-1.8868760	1.2379150
H	-4.1471440	-1.9415340	1.0928730
C	3.8354520	-0.7342160	-0.2428670
C	4.2698080	0.1990970	-1.1991180
C	4.7881180	-1.5616620	0.3780330
C	5.6233030	0.3138370	-1.5113180
H	3.5426340	0.8367830	-1.6881210
C	6.1393520	-1.4409070	0.0668150
H	4.4677080	-2.3149150	1.0907420
C	6.5626710	-0.5018580	-0.8781860
H	5.9443040	1.0438800	-2.2491720
H	6.8625330	-2.0888910	0.5539070
H	7.6173620	-0.4122720	-1.1231630
H	2.5233360	-1.7775810	2.0107730
H	-2.1321710	-1.0476910	1.4094690
C	-0.5551080	0.0931960	-1.7661680
C	0.2380360	0.0206400	-0.4478320
N	-1.9958260	0.1368810	-1.6087730
C	0.2943340	1.3722290	0.2801290
C	-0.2590220	1.5553520	1.5510440
C	-0.2078350	2.8042920	2.1760160
C	0.4013710	3.8840550	1.5390990
C	0.9626360	3.7094730	0.2711430
C	0.9070150	2.4650460	-0.3523830
H	-0.2834410	-0.7882590	-2.3616950
H	-0.2374190	0.9739960	-2.3307770
H	-0.7262200	0.7243050	2.0683940
H	-0.6422040	2.9264850	3.1643640
H	0.4419310	4.8547150	2.0256030
H	1.4443510	4.5432670	-0.2322950
H	1.3529140	2.3401720	-1.3350110

TS **12a** + **6a** → **4a’**

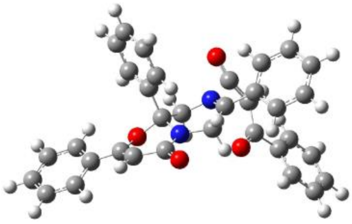

E = -1721.63934117, **H (0K)** = -1721.120293,
H (353K) = -1721.074022, **G (353K)** = -1721.210788 au.
Imaginary frequency = 1.

C	-1.5878380	0.2408860	-0.0200920
N	-1.2134220	0.4685710	1.3632170
O	-2.4980580	-0.8828600	-0.0974820
C	-3.5252710	-0.8947720	0.8021190
C	-3.4422110	-0.1812130	1.9498510
C	-2.1827860	0.4450700	2.3531600
C	0.0979530	1.0070390	1.7063790
O	-1.9516950	0.8638270	3.4883870
C	1.0003140	1.2181310	0.5091380
N	0.8091530	0.6320510	-0.6130750
O	2.3464120	-1.4504220	0.3107480
C	3.2277590	-1.4050380	-0.5534790
C	3.1766900	-0.6168240	-1.7750640
C	2.2483840	0.2209530	-2.2882560
C	-0.3386870	-0.2395260	-0.7785760
O	1.7773170	0.8704130	-3.1468110
H	-4.2540100	-0.1522790	2.6633870
H	0.6103270	0.3083460	2.3840620
H	-0.0245690	1.9412880	2.2635910
H	4.0157130	-0.6802820	-2.4624820
H	-0.5687410	-0.3126410	-1.8446890
H	-0.0697810	-1.2362190	-0.4119460
C	-4.6465260	-1.7592190	0.3945500
C	-4.8203020	-2.1081310	-0.9553450
C	-5.5589510	-2.2507620	1.3450690
C	-5.8900730	-2.9118570	-1.3453330
H	-4.1178460	-1.7406770	-1.6943490
C	-6.6284090	-3.0496320	0.9509360
H	-5.4177740	-2.0243620	2.3970420
C	-6.7990810	-3.3822070	-0.3959660
H	-6.0143060	-3.1694150	-2.3934880
H	-7.3232430	-3.4235570	1.6975940
H	-7.6317250	-4.0098860	-0.7011780
C	4.4784520	-2.2285680	-0.3476000
C	4.7674690	-2.6375280	0.9638450
C	5.3376970	-2.6289410	-1.3821000
C	5.8976410	-3.4025780	1.2394380
H	4.0879990	-2.3406650	1.7555860
C	6.4627970	-3.4081340	-1.1091670
H	5.1223760	-2.3660820	-2.4131870
C	6.7504720	-3.7903240	0.2021500
H	6.1132600	-3.7002460	2.2621440
H	7.1117320	-3.7196990	-1.9231600
H	7.6304170	-4.3918700	0.4139600
C	-2.2489680	1.4564010	-0.6888350
C	-2.7565780	1.3288110	-1.9907950
C	-2.3464590	2.6945800	-0.0469200
C	-3.3462100	2.4145670	-2.6342790

Compound **4a’**

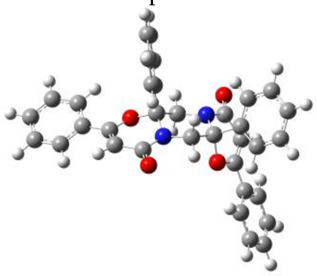

E = -1721.67603233, **H (0K)** = -1721.151436,
H (353K) = -1721.107114, **G (353K)** = -1721.235681 au.
Imaginary frequency = 0.

C	-1.6215848	0.8466533	-0.5076735
N	-0.9424018	0.5222743	0.7436215
O	-2.6645618	-0.1251267	-0.7767925
C	-3.4239568	-0.5673847	0.2637225
C	-2.9902488	-0.4530927	1.5396455
C	-1.6542918	0.0517153	1.8444775
C	0.3143592	1.2193503	0.9838675
O	-1.1653268	0.0437383	2.9744335
C	1.3260512	1.1208233	-0.1652685
N	0.6803712	1.2186303	-1.4539685
O	2.0033882	-0.1713377	-0.0251295
C	2.8442242	-0.4589177	-1.0521465
C	2.7502652	0.2106323	-2.2362495
C	1.5504572	0.9673013	-2.5629915
C	-0.6242778	0.5665783	-1.6417505
O	1.2085572	1.2554683	-3.7031895
H	-3.5936748	-0.7737267	2.3773135
H	0.7571032	0.8009913	1.8874025
H	0.1320512	2.2850043	1.1651175
H	3.4506532	0.0320673	-3.0405755
H	-1.0157738	0.9023353	-2.5999325
H	-0.5172838	-0.5257627	-1.6790145
C	-4.6953088	-1.1740797	-0.1695045
C	-5.2541088	-0.8326087	-1.4123955
C	-5.3683008	-2.0976697	0.6492805
C	-6.4659018	-1.3892407	-1.8173995
H	-4.7387528	-0.1225727	-2.0490385
C	-6.5793858	-2.6498267	0.2413455
H	-4.9310078	-2.3998747	1.5956675
C	-7.1331518	-2.2968877	-0.9925785
H	-6.8902088	-1.1117267	-2.7782595
H	-7.0865138	-3.3655287	0.8822065
H	-8.0763228	-2.7323807	-1.3107285
C	3.8068192	-1.5302007	-0.7382645
C	3.9780272	-1.9574527	0.5902715
C	4.5606192	-2.1507227	-1.7511075
C	4.8901992	-2.9651517	0.8976075
H	3.3907712	-1.4982477	1.3769945
C	5.4749762	-3.1520927	-1.4389075
H	4.4210132	-1.8626517	-2.7877495
C	5.6447202	-3.5625337	-0.1134345
H	5.0115022	-3.2827417	1.9293685
H	6.0491872	-3.6208667	-2.2329625
H	6.3560672	-4.3477047	0.1270455
C	-2.2593778	2.2442503	-0.5273775
C	-2.7301618	2.7790683	-1.7366295

H	-2.6987060	0.3723640	-2.5026890
C	-2.9343130	3.7855880	-0.6928820
H	-1.9742580	2.8163080	0.9649490
C	-3.4347780	3.6498140	-1.9867090
H	-3.7363230	2.2966460	-3.6415000
H	-3.0027980	4.7394650	-0.1772600
H	-3.8925330	4.4979000	-2.4884050
C	2.1673210	2.1170920	0.6940330
C	2.7848760	2.2546200	1.9482770
C	2.6540170	2.8686120	-0.3886530
C	3.8772270	3.1058670	2.1088560
H	2.4310880	1.6816000	2.7997910
C	3.7391140	3.7260410	-0.2221690
H	2.1622850	2.7906730	-1.3521320
C	4.3565830	3.8444790	1.0253710
H	4.3531550	3.1910850	3.0815890
H	4.0988910	4.3075090	-1.0664420
H	5.2034870	4.5128980	1.1537630

C	-2.4523238	2.9806573	0.6486055
C	-3.3636688	4.0200093	-1.7703085
H	-2.6145298	2.2247743	-2.6626785
C	-3.0854758	4.2252623	0.6154385
H	-2.1117498	2.5919653	1.6021405
C	-3.5407308	4.7502393	-0.5929905
H	-3.7176428	4.4162223	-2.7179865
H	-3.2215878	4.7804433	1.5392955
H	-4.0310818	5.7194223	-0.6190705
C	2.3566822	2.2475323	0.0307995
C	3.4152922	2.0825333	0.9328905
C	2.1822482	3.4879123	-0.5947495
C	4.2990822	3.1322353	1.1847495
H	3.5581212	1.1297973	1.4320315
C	3.0673792	4.5370993	-0.3405555
H	1.3553622	3.6270953	-1.2829445
C	4.1297922	4.3626773	0.5472045
H	5.1207192	2.9861433	1.8806725
H	2.9243522	5.4909653	-0.8409235
H	4.8192822	5.1792813	0.7431595

TS **4a' → 4a**

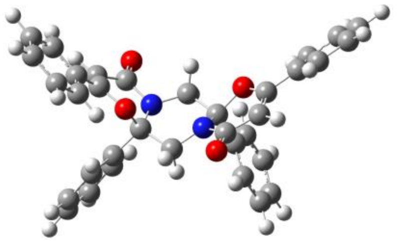

E = -1721.67465751, **H (0K)** = -1721.150104, **H (353K)** = -1721.106720, **G (353K)** = -1721.232039 au.

Imaginary frequency = 1.

C	0.9309810	0.2688409	0.4527782
N	0.1659280	-0.1418691	-0.7156938
O	1.9412420	-0.7309801	0.7610422
C	2.6199830	-1.3037781	-0.2707548
C	2.1189910	-1.2861601	-1.5274168
C	0.7938290	-0.7445871	-1.8074508
C	-1.0545900	0.6122179	-0.9425898
O	0.2425080	-0.8260531	-2.9045468
C	-2.0722850	0.4589949	0.1873152
N	-1.4216270	0.4186859	1.4774362
O	-2.7790410	-0.7933831	-0.0582668
C	-3.6288790	-1.1494791	0.9439332
C	-3.4822810	-0.6168981	2.1864402
C	-2.2253060	0.0279359	2.5596152
C	0.0170150	0.1925319	1.7036002
O	-1.8139910	0.1015059	3.7158582
H	2.6574290	-1.7133431	-2.3617018
H	-1.4948180	0.2592649	-1.8746638
H	-0.8334600	1.6802759	-1.0534778
H	-4.1664520	-0.8522751	2.9898162
H	0.3521410	0.8948629	2.4660192
H	0.1513620	-0.8147301	2.1088262
C	3.8831150	-1.9357631	0.1500362
C	4.5232250	-1.5218971	1.3301932
C	4.4676530	-2.9569201	-0.6195398
C	5.7282120	-2.1036381	1.7199432
H	4.0764530	-0.7372831	1.9299452
C	5.6724620	-3.5334821	-0.2275228

Compound **4a**

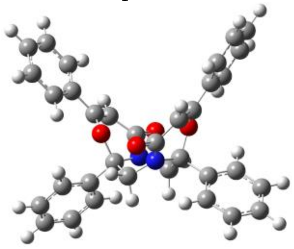

E = -1721.69098009, **H (0K)** = -1721.166429, **H (353K)** = -1721.122108, **G (353K)** = -1721.252353 au.

Imaginary frequency = 0.

C	-1.6781915	-2.0089092	0.4224115
N	-0.8503835	-1.9466442	-0.7708735
O	-2.0661845	-0.6736752	0.8244945
C	-2.5696565	0.1158228	-0.1679385
C	-2.2725775	-0.1462482	-1.4654225
C	-1.2079505	-1.0889202	-1.8080905
C	0.4630375	-2.5649082	-0.7479395
O	-0.6165545	-1.0882142	-2.8865485
C	1.3256775	-2.0002892	0.4016785
N	0.4938315	-1.9123502	1.5903265
O	1.7172875	-0.6713142	-0.0293255
C	2.1982595	0.1464668	0.9501765
C	1.8983625	-0.0886472	2.2522435
C	0.8363225	-1.0273892	2.6094865
C	-0.8116575	-2.5474202	1.5799695
O	0.2356755	-1.0057842	3.6830035
H	-2.6504785	0.4646748	-2.2727605
H	0.9420095	-2.3527152	-1.7037585
H	0.3895245	-3.6520222	-0.6368505
H	2.2885205	0.5321308	3.0464675
H	-0.7250405	-3.6351632	1.4865475
H	-1.2935875	-2.3250362	2.5319655
C	-3.3823805	1.2358648	0.3427165
C	-3.5637215	1.3974228	1.7274955
C	-3.9814865	2.1629478	-0.5294545
C	-4.3242875	2.4549418	2.2237845
H	-3.0969515	0.6976278	2.4104535

H	3.9664970	-3.3144881	-1.5134128
C	6.3078230	-3.1081721	0.9426092
H	6.2156250	-1.7701521	2.6317952
H	6.1106980	-4.3242901	-0.8297188
H	7.2458480	-3.5627231	1.2490722
C	-4.6447660	-2.1338561	0.5299012
C	-4.8428650	-2.4143741	-0.8333538
C	-5.4256590	-2.8156771	1.4807722
C	-5.8062440	-3.3387821	-1.2334828
H	-4.2353080	-1.9083081	-1.5746398
C	-6.3919110	-3.7318321	1.0766572
H	-5.2675120	-2.6421041	2.5400502
C	-6.5872850	-3.9965921	-0.2820678
H	-5.9466650	-3.5435191	-2.2911198
H	-6.9869340	-4.2488771	1.8241402
H	-7.3389470	-4.7161211	-0.5946278
C	1.6649030	1.6121199	0.2727962
C	2.3035660	2.2052709	1.3735522
C	1.7877250	2.2262159	-0.9796728
C	3.0275730	3.3869299	1.2288382
H	2.2537960	1.7370139	2.3520322
C	2.5090270	3.4139699	-1.1247688
H	1.3248780	1.7858259	-1.8559788
C	3.1282160	4.0000369	-0.0222908
H	3.5135450	3.8281629	2.0946152
H	2.5860320	3.8752819	-2.1052948
H	3.6882400	4.9241259	-0.1351278
C	-3.0789260	1.6215489	0.0828742
C	-4.0996180	1.5775219	-0.8756518
C	-2.9187400	2.7795799	0.8515242
C	-4.9583660	2.6638589	-1.0451568
H	-4.2325690	0.6882159	-1.4829628
C	-3.7775740	3.8668299	0.6800342
H	-2.1251030	2.8266919	1.5898802
C	-4.8011570	3.8122939	-0.2668208
H	-5.7510710	2.6106629	-1.7864678
H	-3.6458400	4.7552779	1.2916322
H	-5.4707520	4.6578619	-0.3982378

C	-4.7477755	3.2112008	-0.0297555
H	-3.8533295	2.0674548	-1.6025265
C	-4.9221025	3.3629548	1.3490985
H	-4.4495325	2.5670098	3.2971215
H	-5.2074575	3.9145658	-0.7183405
H	-5.5178915	4.1848848	1.7362965
C	3.0065015	1.2643588	0.4307835
C	3.4927005	1.2344068	-0.8873865
C	3.3006155	2.3801548	1.2352595
C	4.2666595	2.2835148	-1.3803005
H	3.2586595	0.3875038	-1.5213675
C	4.0795565	3.4222358	0.7416605
H	2.9041215	2.4424878	2.2433365
C	4.5671815	3.3777648	-0.5677335
H	4.6354945	2.2443958	-2.4014235
H	4.2968735	4.2768818	1.3760945
H	5.1706015	4.1951368	-0.9527685
C	-2.9375125	-2.8668062	0.2526845
C	-3.9686015	-2.7724922	1.1979735
C	-3.0444205	-3.8035402	-0.7803895
C	-5.0907275	-3.5946992	1.1033895
H	-3.9000815	-2.0427682	1.9988955
C	-4.1678945	-4.6280032	-0.8731945
H	-2.2546115	-3.8797422	-1.5208205
C	-5.1932155	-4.5263142	0.0674645
H	-5.8862205	-3.5056872	1.8381875
H	-4.2414435	-5.3453012	-1.6860535
H	-6.0682025	-5.1662702	-0.0065905
C	2.5853345	-2.8526582	0.5934235
C	3.5779635	-2.8422622	-0.3971825
C	2.7358845	-3.6975202	1.6976685
C	4.7047365	-3.6544542	-0.2781195
H	3.4742375	-2.1888252	-1.2581745
C	3.8649575	-4.5112522	1.8162775
H	1.9748865	-3.7121882	2.4710725
C	4.8516455	-4.4920452	0.8303725
H	5.4687625	-3.6315942	-1.0503635
H	3.9724095	-5.1563232	2.6840025
H	5.7304495	-5.1241042	0.9241045

TS **12a** + **6a** → **5a**

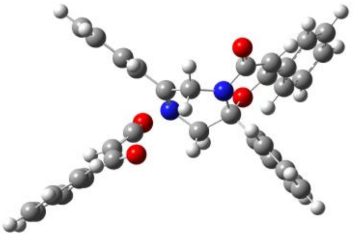

E = -1721.63946394, **H (0K)** = -1721.120277, **H (353K)** = -1721.074203, **G (353K)** = -1721.210613 au.

Imaginary frequency = 1.

C	1.4949700	0.7007660	-0.3756250
N	1.2312480	-0.0188560	-1.6013290
O	2.4522840	-0.0379110	0.4369070
C	3.5733920	-0.4400610	-0.2267930
C	3.5668490	-0.5531000	-1.5794210
C	2.3011600	-0.5567720	-2.3129240
C	-0.1301370	-0.4524450	-1.9078750
O	2.1549510	-1.0701640	-3.4214580
C	-0.7697690	-1.0447910	-0.6619870

Compound **5a**

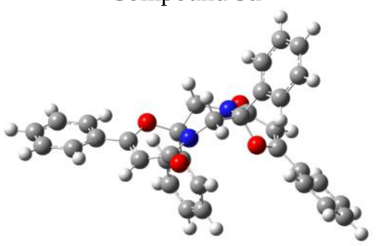

E = -1721.68755909, **H (0K)** = -1721.162957, **H (353K)** = -1721.118733, **G (353K)** = -1721.246710 au.

Imaginary frequency = 0.

C	2.5628342	0.8833408	-0.0035996
N	1.9085452	0.5230958	-1.2480266
O	3.2736082	-0.2659352	0.5287894
C	4.0819612	-0.9064592	-0.3660466
C	3.8746362	-0.7686032	-1.6998986
C	2.6236312	-0.2006802	-2.2021986
C	0.4724692	0.6990768	-1.3790606
O	2.1848072	-0.4008282	-3.3326176

N	-0.5877030	-0.4587800	0.4650730	C	-0.2480178	-0.0578492	-0.2455436
O	-2.8092660	1.0607240	-0.1968700	N	0.3435132	0.3553658	1.0154204
C	-3.5241230	0.7243960	0.7534250	O	-1.6307248	0.3802388	-0.3182236
C	-3.0871210	-0.0138820	1.9274750	C	-2.3821028	0.0599798	0.7766034
C	-1.8904780	-0.5093790	2.3154110	C	-1.7839738	-0.1993732	1.9671744
C	0.2016130	0.7636800	0.4611730	C	-0.3810108	0.1576708	2.1874714
O	-1.1445820	-0.9745310	3.0931720	C	1.4928252	1.2520048	1.0351514
H	4.4499820	-0.8442330	-2.1304340	O	0.1018792	0.3701498	3.2991844
H	-0.0538550	-1.1649010	-2.7279230	H	4.5202572	-1.2412722	-2.4266696
H	-0.7505500	0.3933030	-2.2407020	H	0.1842522	0.3044528	-2.3525626
H	-3.8244220	-0.2343200	2.6947810	H	0.1871282	1.7567428	-1.3333136
H	0.4842800	1.0128830	1.4872760	H	-2.3574008	-0.4835312	2.8384944
H	-0.4346360	1.5699580	0.0792570	H	1.9295242	1.2047158	2.0326844
C	-4.9878120	1.0990860	0.7155220	H	1.1848002	2.2898208	0.8528844
C	-5.5435160	1.3874410	-0.5409490	C	-3.8318318	0.0651248	0.5130354
C	-5.8028400	1.2060780	1.8526810	C	-4.3117828	0.0187588	-0.8070576
C	-6.8834260	1.7455530	-0.6626330	C	-4.7599908	0.1242878	1.5684534
H	-4.9023710	1.3222030	-1.4135350	C	-5.6818418	0.0145468	-1.0628836
C	-7.1420830	1.5798570	1.7334860	H	-3.6057808	-0.0159732	-1.6284296
H	-5.3949220	1.0319790	2.8436290	C	-6.1272348	0.1130328	1.3091554
C	-7.6881810	1.8426870	0.4760730	H	-4.4119518	0.1979928	2.5936374
H	-7.3011510	1.9526350	-1.6442820	C	-6.5940768	0.0571988	-0.0073376
H	-7.7568220	1.6697680	2.6249660	H	-6.0363208	-0.0247192	-2.0890996
H	-8.7329040	2.1274640	0.3843100	H	-6.8305738	0.1597558	2.1357414
C	4.7049860	-0.7461760	0.6673490	H	-7.6619508	0.0536768	-0.2073616
C	4.6805590	-0.3285840	2.0090720	C	5.1169072	-1.7371822	0.2749654
C	5.8219710	-1.4649350	0.2040120	C	5.4110092	-1.5757542	1.6399804
C	5.7506820	-0.6070010	2.8575970	C	5.8210692	-2.7100362	-0.4578156
H	3.8188250	0.2121700	2.3820010	C	6.3935482	-2.3539352	2.2496404
C	6.8917780	-1.7344220	1.0521430	H	4.8653432	-0.8404022	2.2194134
H	5.8477850	-1.8297260	-0.8175360	C	6.8066822	-3.4794172	0.1527944
C	6.8614680	-1.3056750	2.3823640	H	5.5848892	-2.8787412	-1.5032866
H	5.7160700	-0.2754420	3.8916310	C	7.0980112	-3.3041292	1.5088194
H	7.7465000	-2.2905370	0.6775880	H	6.6092742	-2.2153982	3.3053464
H	7.6956100	-1.5217380	3.0441390	H	7.3408002	-4.2262082	-0.4278236
C	2.0364180	2.1184190	-0.6058070	H	7.8642172	-3.9099352	1.9845354
C	2.6281050	2.8179150	0.4552060	C	3.5513162	2.0499128	-0.1524846
C	1.8718840	2.7638120	-1.8361490	C	4.4655982	2.3165318	0.8759824
C	3.0596380	4.1327780	0.2831090	C	3.5026582	2.9061048	-1.2573916
H	2.7633540	2.3296700	1.4152100	C	5.3252622	3.4112628	0.7921904
C	2.3036050	4.0805050	-2.0075380	H	4.5138822	1.6609168	1.7398614
H	1.4135130	2.2338140	-2.6647520	C	4.3631322	4.0029068	-1.3401866
C	2.8993090	4.7685530	-0.9500390	H	2.7995542	2.7108488	-2.0606886
H	3.5233050	4.6594870	1.1127880	C	5.2767602	4.2582838	-0.3173116
H	2.1765760	4.5650320	-2.9717730	H	6.0335732	3.6012698	1.5939154
H	3.2369210	5.7924880	-1.0848300	H	4.3197592	4.6538678	-2.2090656
C	-1.5932010	-2.2692870	-0.7609870	H	5.9473272	5.1106408	-0.3833826
C	-2.2862140	-2.5732260	-1.9443490	C	-0.2014778	-1.5793982	-0.4550516
C	-1.6832220	-3.1542710	0.3272210	C	-0.9607478	-2.1501382	-1.4868496
C	-3.0685160	-3.7239360	-2.0296780	C	0.6170492	-2.4062152	0.3202684
H	-2.2355970	-1.9005350	-2.7953410	C	-0.9137268	-3.5217652	-1.7272196
C	-2.4534370	-4.3105710	0.2332420	H	-1.5963068	-1.5207172	-2.1024996
H	-1.1269280	-2.9389350	1.2331390	C	0.6659722	-3.7812662	0.0772634
C	-3.1516320	-4.5966770	-0.9431180	H	1.2235642	-1.9755962	1.1085054
H	-3.6097900	-3.9397280	-2.9464100	C	-0.1000088	-4.3434062	-0.9430456
H	-2.5043980	-4.9930510	1.0770300	H	-1.5111768	-3.9484852	-2.5281916
H	-3.7523610	-5.4992020	-1.0145790	H	1.3051512	-4.4102272	0.6908694
				H	-0.0621788	-5.4130732	-1.1305426
TS **2a** → **13a**				Compound **13a**			

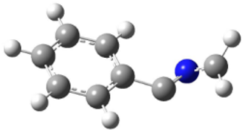

E = -363.67285241, **H (0K)** = -363.548525,
H (353K) = -363.537541, **G (353K)** = -363.588471 au.
Imaginary frequency = 1.

C	3.2774650	-0.0679890	0.4675510
C	1.5644580	-0.5291150	-0.3117240
N	2.4141310	0.4655190	-0.4302380
C	0.1423530	-0.2196850	-0.1411270
C	-0.3604840	1.0976430	-0.0717000
C	-1.7259970	1.3216080	0.0658810
C	-2.6075900	0.2359770	0.1433200
C	-2.1252430	-1.0742370	0.0811910
C	-0.7574570	-1.2988150	-0.0590570
H	3.7682070	0.6160390	1.1628120
H	3.5062630	-1.1255710	0.5307060
H	0.3378290	1.9266240	-0.1302060
H	-2.1106250	2.3367420	0.1160800
H	-3.6740360	0.4144780	0.2542660
H	-2.8140930	-1.9120110	0.1419150
H	-0.3574960	-2.3072480	-0.1099150

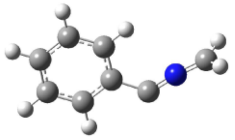

E = -363.73616872, **H (0K)** = -363.610476,
H (353K) = -363.598785, **G (353K)** = -363.651456 au.
Imaginary frequency = 0.

C	-3.5641120	0.3967220	0.0000060
C	-1.4250120	-0.9032210	-0.0000400
N	-2.4371050	-0.2078880	-0.0000280
C	-0.0493760	-0.3837140	0.0000940
C	0.2470610	0.9930820	0.0000480
C	1.5687840	1.4286400	-0.0000130
C	2.6127490	0.4962580	-0.0000130
C	2.3317290	-0.8717020	-0.0000330
C	1.0072900	-1.3082770	0.0000170
H	-4.0458890	0.6438640	0.9418510
H	-4.0458220	0.6439620	-0.9418720
H	-0.5685030	1.7116840	-0.0001290
H	1.7902060	2.4926650	-0.0000210
H	3.1424220	-1.5949660	-0.0000960
H	0.7685030	-2.3675630	0.0000200
H	3.6441350	0.8388420	0.0000510

TS **2b → 13b**

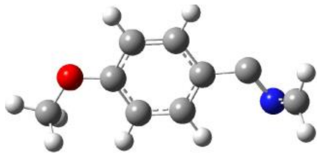

E = -478.19987285, **H (0K)** = -478.042647,
H (353K) = -478.028292, **G (353K)** = -478.087011 au.
Imaginary frequency = 1.

C	4.1474510	-0.3377450	0.5410070
C	2.5491770	0.3812580	-0.2705070
N	3.2504220	-0.7220160	-0.4019080
C	1.1015790	0.2966770	-0.1576840
C	0.3623570	1.4964990	-0.0622600
C	-1.0184500	1.4845250	0.0254160
C	-1.7076710	0.2575480	0.0225430
C	-0.9948440	-0.9523650	-0.0681600
C	0.3911210	-0.9223370	-0.1532480
H	4.5072480	-1.0989570	1.2367470
H	4.5369410	0.6693270	0.6301940
H	0.9059050	2.4367270	-0.0616550
H	-1.5942450	2.4017550	0.0968470
H	-1.5144380	-1.9037100	-0.0693800
H	0.9503650	-1.8504730	-0.2203070
O	-3.0562500	0.3448320	0.1140030
C	-3.8260160	-0.8557730	0.1135110
H	-4.8667230	-0.5387360	0.1905750
H	-3.6832400	-1.4192670	-0.8159860
H	-3.5729970	-1.4909470	0.9705940

Compound **13b**

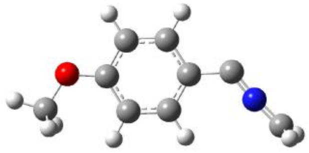

E = -478.26156455, **H (0K)** = -478.103107 ,
H (353K) = -478.088021, **G (353K)** = -478.148452 au.
Imaginary frequency = 0.

C	-4.3281410	-0.9668500	0.0000950
C	-2.4898470	0.7338730	-0.0001850
N	-3.3434730	-0.1498620	-0.0001880
C	-1.0430570	0.5184230	-0.0001290
C	-0.1924460	1.6409460	0.0000550
C	1.1859740	1.4959100	0.0001330
C	1.7560510	0.2115820	0.0000120
C	0.9263870	-0.9225290	-0.0002340
C	-0.4553450	-0.7592180	-0.0003330
H	-4.7531300	-1.3049250	-0.9412170
H	-4.7531210	-1.3042660	0.9416540
H	-0.6398460	2.6303950	0.0001670
H	1.8469230	2.3568900	0.0003020
H	1.3478280	-1.9212330	-0.0003770
H	-1.0964720	-1.6371680	-0.0005560
O	3.1138320	0.1701400	0.0000600
C	3.7586530	-1.0997380	0.0004510
H	4.8288890	-0.8885370	0.0008480
H	3.5012430	-1.6787200	0.8957600
H	3.5019530	-1.6789130	-0.8949400

TS **2c → 13c**

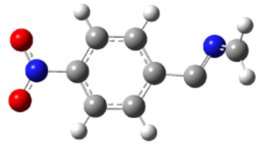

E = -568.17256743, **H (0K)** = -568.046006,
H (353K) = -568.031598, **G (353K)** = -568.092486 au.

Compound **13c**

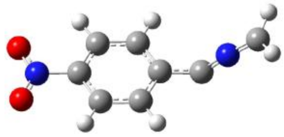

E = -568.23868497, **H (0K)** = -568.110683,
H (353K) = -568.095540, **G (353K)** = -568.159259 au.

Imaginary frequency = 1.

C	-4.4182900	-0.2381360	0.5281620
C	-2.7868330	0.5434700	-0.1848580
N	-3.5573040	-0.4779820	-0.4867720
C	-1.3347700	0.3093230	-0.1241720
C	-0.7579660	-0.9783460	-0.1166920
C	0.6205150	-1.1348070	-0.0517830
C	1.4177540	0.0107890	-0.0031610
C	0.8855200	1.2991660	-0.0105910
C	-0.4977800	1.4385970	-0.0584990
H	-4.8175510	-1.1017980	1.0628690
H	-4.7184330	0.7557680	0.8413990
H	-1.4069430	-1.8462610	-0.1638200
H	1.0852040	-2.1129270	-0.0456700
H	1.5462480	2.1557350	0.0312110
H	-0.9534990	2.4233150	-0.0501820
N	2.8818820	-0.1491900	0.0632570
O	3.5697100	0.8710320	0.0924540
O	3.3332940	-1.2940260	0.0848420

Imaginary frequency = 0.

C	-4.7905210	-0.0907710	0.6992090
C	-2.6934570	0.0944260	-0.6306930
N	-3.6773870	-0.0038350	0.0740880
C	-1.2768270	0.0609050	-0.3738640
C	-0.5918920	-1.1733580	-0.3148320
C	0.7822440	-1.2031490	-0.1346200
C	1.4837420	0.0000280	-0.0199680
C	0.8298120	1.2333590	-0.0880810
C	-0.5431100	1.2638840	-0.2761550
H	-5.2415740	-1.0651600	0.8519250
H	-5.2628580	0.8099840	1.0756740
H	-1.1455430	-2.1018570	-0.4076490
H	1.3187290	-2.1420180	-0.0752960
H	1.4025630	2.1478410	0.0036420
H	-1.0590950	2.2158780	-0.3445070
N	2.9266600	-0.0322610	0.1726080
O	3.5239150	1.0440550	0.2747780
O	3.4814500	-1.1345480	0.2256430

Compound **14a**

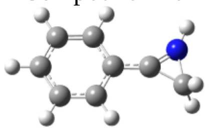

E = -364,15046235, **H (0K)** = -364,010570,
H (353K) = -363,998908, **G (353K)** = -364,050900 au.
Imaginary frequency = 0.

C	2.8457850	-0.7076810	0.0003530
C	1.5464200	0.0242790	-0.0004690
C	0.1349960	0.0224270	-0.0001390
C	-0.5347290	-1.2235400	-0.0000890
C	-1.9218640	-1.2499730	0.0000780
C	-2.6383920	-0.0470000	0.0001260
C	-1.9789720	1.1917890	0.0000610
C	-0.5946050	1.2365120	-0.0000510
H	3.2119400	-1.1400510	-0.9276600
H	3.2104350	-1.1396470	0.9291770
H	0.0404630	-2.1441410	-0.0002180
H	-2.4486100	-2.1980230	0.0001330
H	-3.7238980	-0.0719770	0.0001960
H	-2.5527280	2.1122790	0.0000720
H	-0.0725450	2.1885100	-0.0001150
N	2.5982940	0.7471600	-0.0006350
H	2.9950530	1.6820600	0.0036350

Compound **14c**

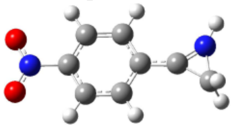

E = -568,63686683, **H (0K)** = -568,494582,
H (353K) = -568,479738, **G (353K)** = -568,540228 au.
Imaginary frequency = 0.

C	4.0102640	-0.7345940	0.0005820
C	2.7202730	0.0125900	-0.0003000
C	1.3001940	0.0293780	-0.0001500
C	0.6195010	-1.2075080	-0.0002920
C	-0.7690690	-1.2248160	-0.0002160
C	-1.4432390	-0.0055380	0.0000090
C	-0.7963470	1.2318030	0.0001530
C	0.5894710	1.2516280	0.0000620
H	4.3730970	-1.1670900	-0.9283830
H	4.3719620	-1.1661180	0.9304660
H	1.1794090	-2.1369680	-0.0005060
H	-1.3285650	-2.1514230	-0.0003370
H	-1.3790670	2.1439380	0.0003360
H	1.1179820	2.1993890	0.0001710
N	3.7731590	0.7259670	-0.0006810
H	4.1752080	1.6601660	0.0020420
N	-2.9293170	-0.0213070	0.0001030
O	-3.4991500	1.0646110	0.0002620
O	-3.4762510	-1.1186320	-0.0001170

TS **2a** + **14a** → **15a**

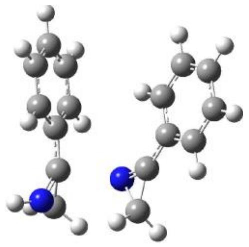

E = -727.91117808, **H (0K)** = -727.642511,
H (353K) = -727.619872, **G (353K)** = -727.701120 au.
Imaginary frequency = 1.

C	-2.9530670	-2.2672360	-0.5418550
C	-2.2305900	-1.1343690	0.0626050

Compound **15a**

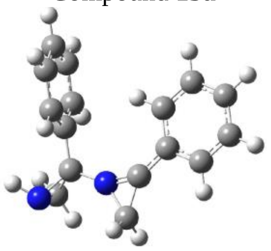

E = -727.94328161, **H (0K)** = -727.670524,
H (353K) = -727.648776, **G (353K)** = -727.725900 au.
Imaginary frequency = 0.

C	-2.5699750	-2.1509450	-0.9135600

C	-2.1170470	0.2927740	0.0889520
C	-1.8997540	0.9765250	1.3026770
C	-1.8383700	2.3644360	1.3039290
C	-1.9883030	3.0715030	0.1044840
C	-2.1953100	2.3956030	-1.1038930
C	-2.2539100	1.0069500	-1.1183010
H	-2.4156300	-3.0184830	-1.1102380
H	-4.0068380	-2.1222070	-0.7748940
H	-1.7836870	0.4227250	2.2295690
H	-1.6768540	2.8989550	2.2343770
H	-1.9429370	4.1564120	0.1121000
H	-2.3105150	2.9532390	-2.0276300
H	-2.4047030	0.4661250	-2.0473650
N	-2.5164750	-2.1282970	0.8672360
H	-3.0004650	-2.1539350	1.7603160
C	0.6543510	-2.9361500	-0.0414800
C	1.0901000	-1.5545370	-0.2380960
N	-0.1321830	-1.7298530	-0.5408700
C	2.1313220	-0.5674050	-0.1439050
C	3.4071690	-0.9594080	0.2984060
C	4.4251690	-0.0153300	0.3966910
C	4.1749800	1.3160690	0.0521760
C	2.9067730	1.7101730	-0.3920960
C	1.8835740	0.7751500	-0.4917580
H	0.4305880	-3.2901520	0.9655390
H	0.8789100	-3.7053240	-0.7782450
H	3.5856540	-1.9980710	0.5599630
H	5.4113690	-0.3141990	0.7378070
H	4.9714170	2.0508200	0.1263870
H	2.7236980	2.7457750	-0.6618760
H	0.8980760	1.0668830	-0.8415830

C	-1.6723080	-1.2762170	-0.1013010
C	-1.8525580	0.2066420	0.0067690
C	-1.7822720	0.8217870	1.2657010
C	-1.9395330	2.2027660	1.3767280
C	-2.1634150	2.9751810	0.2334540
C	-2.2315930	2.3663030	-1.0217270
C	-2.0753170	0.9837540	-1.1371680
H	-2.1668560	-3.0212490	-1.4238010
H	-3.3988570	-1.6576740	-1.4157750
H	-1.6135970	0.2203560	2.1553470
H	-1.8922560	2.6745540	2.3534920
H	-2.2895450	4.0501680	0.3220130
H	-2.4106830	2.9648160	-1.9098170
H	-2.1288630	0.5083720	-2.1125800
N	-2.5593450	-2.2330090	0.5521210
H	-3.3510880	-1.7402820	0.9662130
C	0.5753730	-2.8755030	0.2598430
C	0.9596400	-1.4495300	0.0590140
N	-0.2897350	-1.7009140	0.0154210
C	1.9883310	-0.4748670	-0.0227450
C	3.3179450	-0.9182530	0.1581160
C	4.3607820	-0.0043520	0.0932260
C	4.0848580	1.3453140	-0.1524330
C	2.7680480	1.7895680	-0.3364740
C	1.7141370	0.8900580	-0.2737720
H	0.5565840	-3.2882400	1.2659620
H	0.6844100	-3.5793670	-0.5629670
H	3.5106710	-1.9696870	0.3463700
H	5.3842130	-0.3365530	0.2315590
H	4.9020160	2.0587040	-0.2032750
H	2.5715550	2.8392320	-0.5285070
H	0.6929840	1.2240810	-0.4190520

TS **15a → 15a-conformer**

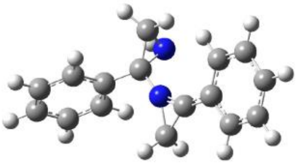

E = -727.93802996, **H (0K)** = -727.665424,
H (353K) = -727.644662, **G (353K)** = -727.718977 au.
Imaginary frequency = 1.

C	-0.6455970	2.1971220	-0.1973210
C	-1.0412880	0.7792800	-0.4028190
C	-2.3949180	0.2568870	-0.0223550
C	-3.1716490	-0.4251040	-0.9693860
C	-4.4252050	-0.9233840	-0.6153020
C	-4.9048250	-0.7484100	0.6852160
C	-4.1304380	-0.0754910	1.6328010
C	-2.8760820	0.4247550	1.2819190
H	0.3784370	2.4595360	0.0493940
H	-1.4014780	2.8502540	0.2318740
H	-2.8001540	-0.5596490	-1.9825190
H	-5.0275560	-1.4438130	-1.3537500
H	-5.8824120	-1.1342910	0.9587230
H	-4.5030010	0.0627160	2.6433210
H	-2.2733230	0.9492380	2.0184080
N	-0.8358140	1.6603510	-1.5522840
H	-1.7386510	1.9323510	-1.9434640
C	0.1720770	-1.7086020	-0.5281870
C	1.1824170	-0.6662390	-0.2039220

Compound **15a-conformer**

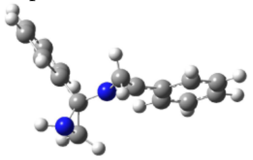

E = -727.94048392, **H (0K)** = -727.667141,
H (353K) = -727.645542, **G (353K)** = -727.722136 au.
Imaginary frequency = 0.

C	0.8956940	-1.2435190	2.1475590
C	1.1560510	-0.9999030	0.7021950
C	2.1590480	-0.0101440	0.1872150
C	3.0703440	-0.4013070	-0.8050890
C	3.9966460	0.5150250	-1.3015040
C	4.0159720	1.8241150	-0.8133860
C	3.1089230	2.2172020	0.1730750
C	2.1790120	1.3039790	0.6708560
H	-0.1044700	-1.4969860	2.4888220
H	1.5041640	-0.6866490	2.8556620
H	3.0630320	-1.4222700	-1.1785610
H	4.7055490	0.2068580	-2.0640730
H	4.7405480	2.5355290	-1.1983600
H	3.1270830	3.2325770	0.5574220
H	1.4761950	1.6088880	1.4415360
N	1.5354630	-2.2655880	1.3002320
H	2.5450660	-2.2975810	1.4433190
C	-0.4475800	-1.9197730	-1.4083770
C	-1.1599840	-0.9118900	-0.5762450

N	-0.0231020	-0.2574340	-0.3109560
C	2.5496540	-0.3634380	0.0232470
C	3.4614880	-1.4428350	-0.0295640
C	4.8148650	-1.2106750	0.1735530
C	5.2638590	0.0889730	0.4315580
C	4.3650800	1.1637450	0.4887800
C	3.0110710	0.9471290	0.2865190
H	-0.1975900	-2.3642320	0.2569410
H	0.0955930	-2.0667310	-1.5532410
H	3.0945050	-2.4440710	-0.2315080
H	5.5196810	-2.0343190	0.1317310
H	6.3229140	0.2689360	0.5897360
H	4.7289180	2.1658880	0.6896270
H	2.3134820	1.7755010	0.3289970

N	0.0301030	-1.1837860	-0.2110350
C	-2.3262390	-0.1503350	-0.3068710
C	-3.4596650	-0.3517710	-1.1246410
C	-4.6189410	0.3708250	-0.8752350
C	-4.6503010	1.2910530	0.1785430
C	-3.5255630	1.4987890	0.9898480
C	-2.3615130	0.7833730	0.7544840
H	-0.0271080	-1.6173530	-2.3653880
H	-0.6627160	-2.9766910	-1.2688950
H	-3.4150850	-1.0711550	-1.9362320
H	-5.4963060	0.2218990	-1.4956370
H	-5.5582530	1.8550560	0.3699700
H	-3.5655520	2.2194140	1.7999540
H	-1.4825210	0.9397620	1.3715170

TS **15a-conformer → 16a**

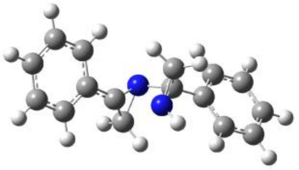

E = -727.90728565, **H (0K)** = -727.635485, **H (353K)** = -727.615058, **G (353K)** = -727.688062 au.Imaginary frequency = 1.

C	0.2264710	0.8491190	1.5037500
C	0.8893730	0.2684710	0.3051680
C	2.3664490	0.1461110	0.1454810
C	3.0777520	-0.9340300	0.6790230
C	4.4631030	-1.0072900	0.5174630
C	5.1382750	-0.0106070	-0.1865770
C	4.4277590	1.0637370	-0.7305220
C	3.0472000	1.1453730	-0.5656200
H	-0.6638510	1.4669090	1.4191230
H	0.8681690	1.0765470	2.3501110
H	2.5644990	-1.7261050	1.2183250
H	5.0089250	-1.8472650	0.9361880
H	6.2148830	-0.0702480	-0.3147420
H	4.9502080	1.8398660	-1.2815280
H	2.4950650	1.9796460	-0.9887220
N	0.0466790	-0.5811490	1.1969340
H	0.4599010	-1.2461530	1.8534740
C	-0.1244120	-1.1067790	-1.4213600
C	-1.0603770	-0.4143220	-0.5529200
N	0.0844340	0.3053970	-0.9188610
C	-2.4310730	-0.1616380	-0.2890730
C	-3.3400940	-1.2451760	-0.2500070
C	-4.6822570	-1.0042270	-0.0005790
C	-5.1310840	0.3102070	0.1912480
C	-4.2421160	1.3904700	0.1332870
C	-2.8923340	1.1631180	-0.0980080
H	-0.3513600	-1.1615600	-2.4822150
H	0.4963710	-1.9020170	-1.0177770
H	-2.9774570	-2.2564520	-0.4051990
H	-5.3853900	-1.8297160	0.0369300
H	-6.1855670	0.4938040	0.3742240
H	-4.6081490	2.4040450	0.2593570
H	-2.1998380	1.9937190	-0.1885830

Compound **16a**

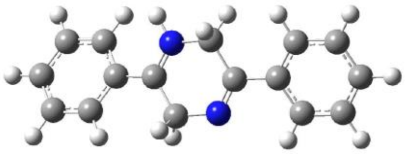

E = -728.04681665, **H (0K)** = -727.770821, **H (353K)** = -727.749947, **G (353K)** = -727.824725 au. Imaginary frequency = 0.

C	0.7005440	1.2184960	0.5432480
C	1.4085320	-0.0851000	0.2067480
C	2.8773490	-0.0341710	0.0196480
C	3.6167220	1.1350140	0.2682540
C	5.0006450	1.1469630	0.0959430
C	5.6624560	-0.0041390	-0.3317630
C	4.9354280	-1.1728800	-0.5823350
C	3.5568990	-1.1906240	-0.4061020
H	1.0422450	2.0359140	-0.0969140
H	0.8990230	1.5049920	1.5836230
H	3.1324280	2.0452030	0.6087360
H	5.5591730	2.0561950	0.2964850
H	6.7397190	0.0071840	-0.4690940
H	5.4469160	-2.0709190	-0.9158330
H	2.9857630	-2.0923820	-0.5959630
N	-0.7517820	1.1082280	0.3643600
H	-1.2619290	1.9879900	0.3635790
C	-0.6390750	-1.2871350	0.3015710
C	-1.4236690	-0.0022040	0.2367270
N	0.7886680	-1.1979670	0.0971900
C	-2.8651820	0.0131040	0.0214230
C	-3.6322770	-1.1114380	0.3904760
C	-5.0128590	-1.0996150	0.2249700
C	-5.6417940	0.0179530	-0.3299170
C	-4.8884340	1.1297700	-0.7222720
C	-3.5107820	1.1330560	-0.5464810
H	-1.0543350	-1.9827930	-0.4355080
H	-0.8250080	-1.7518770	1.2831260
H	-3.1579050	-1.9823780	0.8300660
H	-5.5978030	-1.9624130	0.5260490
H	-6.7186030	0.0199130	-0.4677350
H	-5.3754440	1.9865390	-1.1763340
H	-2.9394650	1.9847130	-0.9059690

TS **2c + 14c → 15c**

Compound **15c**

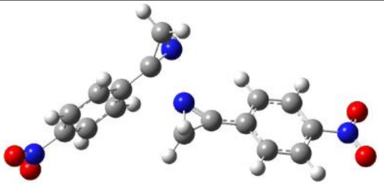

E = -1136.89454703, **H (0K)** = -1136.620781,
H (353K) = -1136.591633, **G (353K)** = -1136.690005 au.
Imaginary frequency = 1.

C	-0.8990090	3.2679120	0.5144070
C	-1.3428120	1.9565170	0.0044600
C	-2.4728930	1.0740510	-0.1253090
C	-2.7030130	0.3908140	-1.3347060
C	-3.8158490	-0.4302260	-1.4595320
C	-4.6761290	-0.5520630	-0.3685150
C	-4.4695580	0.1098940	0.8404460
C	-3.3520870	0.9266370	0.9632860
H	-0.0974580	3.3284860	1.2424940
H	-1.6183800	4.0849540	0.4929090
H	-2.0184090	0.5051440	-2.1690820
H	-4.0267130	-0.9684810	-2.3748770
H	-5.1712920	-0.0214740	1.6542420
H	-3.1554830	1.4506490	1.8926920
N	-0.5267380	2.6224880	-0.7672030
H	-0.5733010	2.8820740	-1.7489150
C	-0.0403020	-0.6373180	2.1434710
C	1.0188670	-0.1295690	1.2674590
N	-0.0455820	0.5549850	1.1955210
C	2.3603610	-0.2431500	0.7482540
C	3.1899560	-1.2731660	1.2223200
C	4.4887270	-1.3973610	0.7396920
C	4.9318740	-0.4826560	-0.2122600
C	4.1293780	0.5494000	-0.7005220
C	2.8345530	0.6682130	-0.2148400
H	-0.0333460	-0.4093250	3.2074250
H	-0.6187250	-1.5091510	1.8412940
H	2.8149500	-1.9691610	1.9653060
H	5.1518150	-2.1794850	1.0868820
H	4.5259510	1.2338050	-1.4396020
H	2.1873170	1.4622780	-0.5732840
N	6.3122510	-0.6081800	-0.7281200
O	6.6752570	0.2042730	-1.5755700
O	7.0052110	-1.5157450	-0.2763550
N	-5.8642040	-1.4281720	-0.5011910
O	-6.6190190	-1.5019060	0.4636080
O	-6.0101250	-2.0198380	-1.5665830

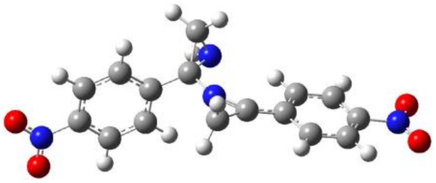

E = -1136.89454703, **H (0K)** = -1136.620781,
H (353K) = -1136.591633, **G (353K)** = -1136.690005 au.
Imaginary frequency = 0.

C	-0.7006570	2.7312240	0.0822440
C	-0.9738700	1.2605950	0.0995330
C	-2.3504710	0.6762460	-0.0261410
C	-2.5367470	-0.4772620	-0.8034130
C	-3.8029040	-1.0398980	-0.9312820
C	-4.8686300	-0.4345390	-0.2687410
C	-4.7110640	0.7054310	0.5136510
C	-3.4380810	1.2590200	0.6356050
H	0.0935570	3.1360490	0.7027540
H	-1.5502530	3.3885810	-0.0837490
H	-1.6956350	-0.9311190	-1.3200310
H	-3.9732930	-1.9251230	-1.5309130
H	-5.5681940	1.1404510	1.0119740
H	-3.2963760	2.1446600	1.2466990
N	-0.2514100	1.8579750	-1.0117810
H	-0.8503310	1.9281430	-1.8353390
C	0.0232240	-0.3825260	2.1411090
C	1.0597810	-0.0533710	1.1218120
N	-0.0983550	0.4330820	0.9087510
C	2.4025030	-0.1454470	0.6563300
C	3.3192530	-0.9001110	1.4195120
C	4.6365130	-1.0107740	0.9957210
C	5.0080920	-0.3623450	-0.1809870
C	4.1227520	0.3918330	-0.9524030
C	2.8044620	0.5017290	-0.5343120
H	0.0169380	0.1546220	3.0873830
H	-0.4568340	-1.3582980	2.1198540
H	2.9957360	-1.3900720	2.3319110
H	5.3683060	-1.5811080	1.5532600
H	4.4728840	0.8728980	-1.8568610
H	2.0799020	1.0760630	-1.1036060
N	6.4161080	-0.4810760	-0.6359560
O	6.7236510	0.1076220	-1.6675030
O	7.1722740	-1.1593310	0.0518900
N	-6.2160480	-1.0258950	-0.4011830
O	-7.1406480	-0.4656310	0.1823640
O	-6.3244530	-2.0405580	-1.0856980

TS **15c → 15c-conformer**

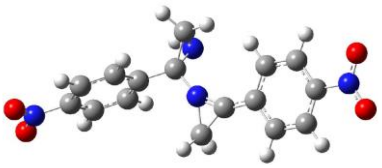

E = -1136.92062455, **H (0K)** = -1136.643764,
H (353K) = -1136.616169, **G (353K)** = -1136.709387 au.
Imaginary frequency = 1.

C	-0.5121830	2.4377220	0.0527800
C	-1.0147480	1.1327680	-0.4572580
C	-2.4128730	0.6528680	-0.1884920

Compound **15c-conformer**

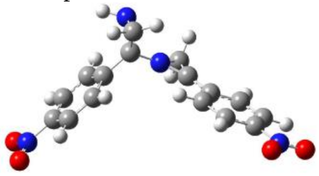

E = -1136.92291750, **H (0K)** = -1136.645212,
H (353K) = -1136.616824, **G (353K)** = -1136.712372 au.
Imaginary frequency = 0.

C	-0.7216220	2.2266370	2.1137630
C	-1.1328120	1.8154460	0.7421610
C	-2.2189830	0.8197400	0.4522540

C	-3.2534030	0.3128140	-1.2575780
C	-4.5457710	-0.1452700	-1.0149480
C	-4.9729740	-0.2593160	0.3056730
C	-4.1581580	0.0686720	1.3859810
C	-2.8675650	0.5255000	1.1304200
H	0.5228620	2.5594000	0.3557940
H	-1.2245970	3.0393780	0.6124700
H	-2.9051950	0.4122610	-2.2816430
H	-5.2158910	-0.4081820	-1.8236620
H	-4.5349670	-0.0321950	2.3959900
H	-2.2177150	0.7894990	1.9592100
N	-0.7186180	2.2198710	-1.3835600
H	-1.5812280	2.6498210	-1.7201820
C	-0.0233950	-1.3909900	-1.0765480
C	1.0665360	-0.5178580	-0.5680770
N	-0.0921490	0.0130940	-0.5944840
C	2.4569670	-0.3878220	-0.2803750
C	3.2642230	-1.5223920	-0.5154840
C	4.6278210	-1.4664140	-0.2594480
C	5.1585910	-0.2750630	0.2289090
C	4.3864370	0.8617220	0.4733440
C	3.0251600	0.8052000	0.2174730
H	-0.4574070	-2.1428750	-0.4217780
H	-0.1194270	-1.5441190	-2.1494410
H	2.8185850	-2.4348730	-0.8969940
H	5.2741880	-2.3178810	-0.4300740
H	4.8558020	1.7602900	0.8532580
H	2.4093370	1.6770950	0.3988140
N	6.6165310	-0.2102750	0.5021810
O	7.0550780	0.8434900	0.9515150
O	7.2768360	-1.2145400	0.2584390
N	-6.3422600	-0.7468440	0.5703280
O	-6.6959090	-0.8342220	1.7437540
O	-7.0381120	-1.0361500	-0.4001160

C	-3.1820120	1.1200770	-0.5235600
C	-4.1922520	0.2082110	-0.8147790
C	-4.2188020	-0.9988650	-0.1199120
C	-3.2752710	-1.3232350	0.8510370
C	-2.2669400	-0.4041330	1.1315950
H	0.3157810	2.4729250	2.3232640
H	-1.2763980	1.7961360	2.9438190
H	-3.1521410	2.0710930	-1.0480690
H	-4.9508010	0.4191690	-1.5580050
H	-3.3384460	-2.2721330	1.3686920
H	-1.5230440	-0.6378160	1.8869920
N	-1.4036250	3.1599760	1.2014890
H	-2.3913540	3.2748760	1.4297730
C	0.2808090	2.4327430	-1.6056340
C	1.0342500	1.4828720	-0.7433190
N	-0.1069230	1.8293120	-0.3010580
C	2.2053590	0.7078080	-0.4996300
C	3.2514420	0.7749520	-1.4430510
C	4.4144500	0.0449880	-1.2326630
C	4.5027150	-0.7392960	-0.0847060
C	3.4805760	-0.8299600	0.8618460
C	2.3205730	-0.1001340	0.6530740
H	-0.2383210	2.0572730	-2.4846510
H	0.5399470	3.4886150	-1.5860780
H	3.1454230	1.3966510	-2.3257530
H	5.2395850	0.0736040	-1.9327760
H	3.6085250	-1.4598690	1.7328860
H	1.5099660	-0.1516820	1.3717460
N	5.7454970	-1.5185980	0.1454270
O	5.7948260	-2.2057050	1.1604970
O	6.6331420	-1.4178850	-0.6945600
N	-5.2903770	-1.9695160	-0.4229730
O	-5.2952930	-3.0237140	0.2080510
O	-6.1063770	-1.6607180	-1.2881000

TS **15c-conformer** → **16c**

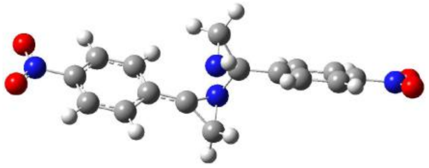

E = -1136.89023293, **H (0K)** = -1136.614051, **H (353K)** = -1136.586824, **G (353K)** = -1136.678177 au.

Imaginary frequency = 1.

C	0.1165600	0.6674610	1.5299260
C	0.8551270	0.1843350	0.3319290
C	2.3433740	0.1312390	0.2387880
C	3.0716520	-0.9493970	0.7488850
C	4.4622300	-0.9658320	0.6546070
C	5.0995500	0.1039120	0.0357420
C	4.3970860	1.1872540	-0.4894010
C	3.0108190	1.1969380	-0.3841090
H	-0.7857330	1.2660370	1.4345570
H	0.7074240	0.8647640	2.4200010
H	2.5701630	-1.7912740	1.2162170
H	5.0456730	-1.7912200	1.0424780
H	4.9335980	1.9991870	-0.9639230
H	2.4467560	2.0316520	-0.7878670
N	0.0025550	-0.7452620	1.1287450
H	0.4065900	-1.4338940	1.7669350

Compound **16c**

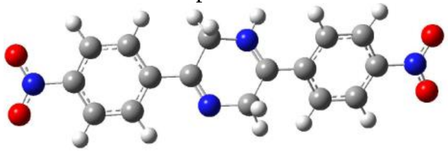

E = -1137.03202821, **H (0K)** = -1136.751330, **H (353K)** = -1136.723800, **G (353K)** = -1136.815948 au.

Imaginary frequency = 0.

C	-0.6792520	1.4207120	0.2881850
C	-1.4019170	0.0867280	0.4523650
C	-2.8661470	0.0497370	0.2108090
C	-3.5249160	-1.1937780	0.1904540
C	-4.8949880	-1.2644080	-0.0203190
C	-5.6058770	-0.0788580	-0.2052330
C	-4.9883960	1.1670020	-0.1823000
C	-3.6127730	1.2251680	0.0239020
H	-0.8005370	2.0351110	1.1895690
H	-1.0583690	1.9898600	-0.5624660
H	-2.9486630	-2.0991160	0.3401880
H	-5.4152280	-2.2136980	-0.0441930
H	-5.5774470	2.0642670	-0.3224020
H	-3.1432190	2.2027550	0.0511180
N	0.7547100	1.2017990	0.0533640
H	1.2483450	1.9315370	-0.4563740

C	-0.0271450	-1.1093630	-1.5433380	C	0.6270790	-0.9506030	1.0848350
C	-1.0233400	-0.4852010	-0.6919250	C	1.4136270	0.1415900	0.4234300
N	0.1208220	0.2676870	-0.9303170	N	-0.7913760	-0.9793910	0.7998470
C	-2.4122570	-0.2848230	-0.4526900	C	2.8590200	0.0560850	0.2059350
C	-3.2868990	-1.3934030	-0.5097120	C	3.6514270	1.2199340	0.3002440
C	-4.6431120	-1.2115140	-0.2879190	C	5.0232060	1.1529170	0.0956520
C	-5.1061720	0.0806370	-0.0336490	C	5.5903760	-0.0817530	-0.2107480
C	-4.2732210	1.1977340	0.0035420	C	4.8387460	-1.2487140	-0.3078890
C	-2.9122100	1.0137330	-0.2009320	C	3.4672290	-1.1798920	-0.0889840
H	-0.1760370	-1.0902970	-2.6191570	H	0.7754990	-0.8500570	2.1727060
H	0.5698520	-1.9274460	-1.1477590	H	1.0625940	-1.9212490	0.8314860
H	-2.8939350	-2.3833870	-0.7152570	H	3.2111850	2.1701270	0.5873270
H	-5.3414590	-2.0382380	-0.3165440	H	5.6509890	2.0310630	0.1788250
H	-4.6939980	2.1788710	0.1835160	H	5.3238450	-2.1854980	-0.5517450
H	-2.2441020	1.8680740	-0.2201060	H	2.8776100	-2.0836740	-0.1897170
N	-6.5595590	0.2751500	0.1977700	N	-7.0623370	-0.1456830	-0.4314360
O	-6.9453930	1.4209780	0.4047620	O	-7.6597850	0.9148840	-0.6045120
O	-7.2691310	-0.7249890	0.1693000	O	-7.5862290	-1.2573670	-0.4334460
N	6.5715210	0.0877690	-0.0732380	N	7.0529430	-0.1549470	-0.4369470
O	7.1089170	1.0381960	-0.6367480	O	7.5265790	-1.2570700	-0.6939740
O	7.1650540	-0.8757480	0.4061530	O	7.6865790	0.8926700	-0.3530850

.
